# Supplementary figures and images for: A method for detecting the quality of cotton seeds based on an improved ResNet50 model (part 2 of 2)
Source: PLoS One. 2023 Feb 15;18(2):e0273057. doi: 10.1371/journal.pone.0273057 (PMC9931132; doi:10.1371/journal.pone.0273057)

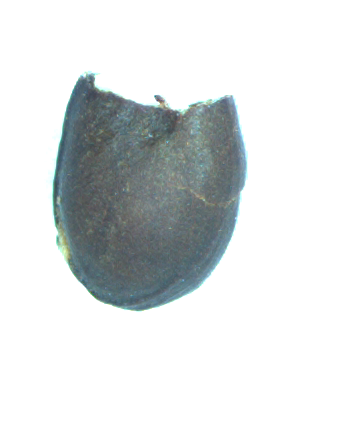

Supplement: S1 Data — (ZIP) [file pone.0273057.s001.zip › Supporting Information/Broken cotton seed/Image_54.bmp]

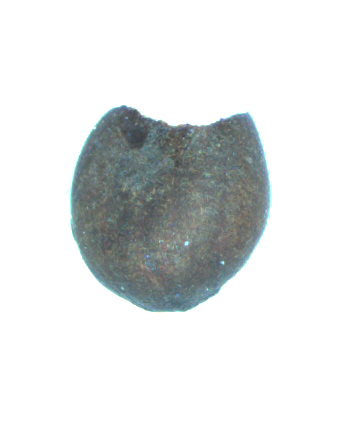

Supplement: S1 Data — (ZIP) [file pone.0273057.s001.zip › Supporting Information/Broken cotton seed/Image_55.bmp]

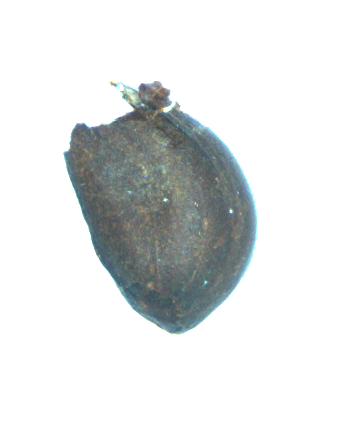

Supplement: S1 Data — (ZIP) [file pone.0273057.s001.zip › Supporting Information/Broken cotton seed/Image_56.bmp]

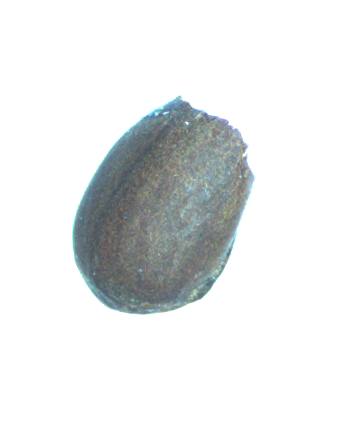

Supplement: S1 Data — (ZIP) [file pone.0273057.s001.zip › Supporting Information/Broken cotton seed/Image_57.bmp]

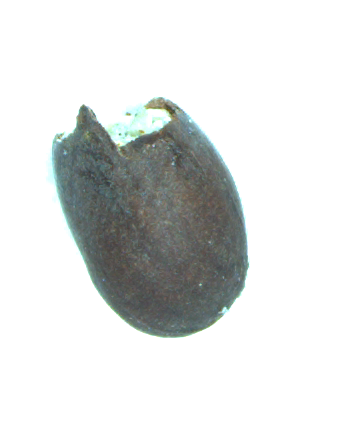

Supplement: S1 Data — (ZIP) [file pone.0273057.s001.zip › Supporting Information/Broken cotton seed/Image_58.bmp]

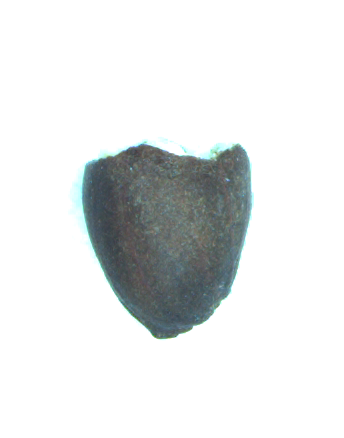

Supplement: S1 Data — (ZIP) [file pone.0273057.s001.zip › Supporting Information/Broken cotton seed/Image_59.bmp]

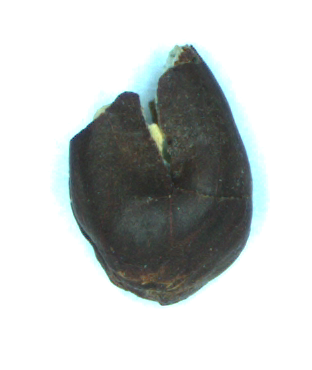

Supplement: S1 Data — (ZIP) [file pone.0273057.s001.zip › Supporting Information/Broken cotton seed/Image_6.bmp]

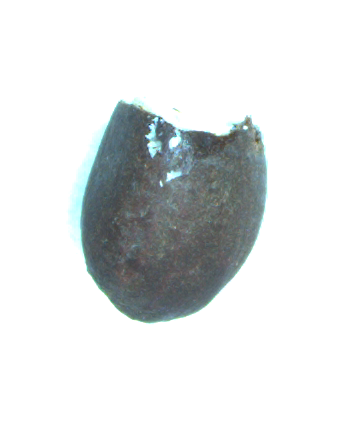

Supplement: S1 Data — (ZIP) [file pone.0273057.s001.zip › Supporting Information/Broken cotton seed/Image_60.bmp]

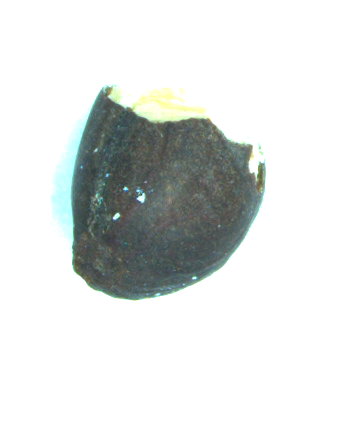

Supplement: S1 Data — (ZIP) [file pone.0273057.s001.zip › Supporting Information/Broken cotton seed/Image_61.bmp]

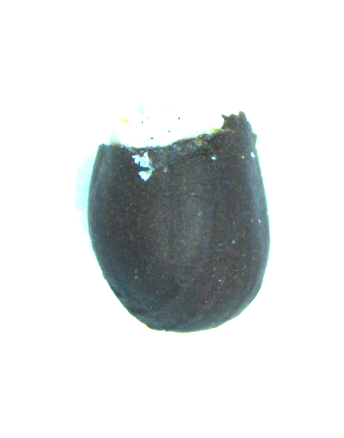

Supplement: S1 Data — (ZIP) [file pone.0273057.s001.zip › Supporting Information/Broken cotton seed/Image_62.bmp]

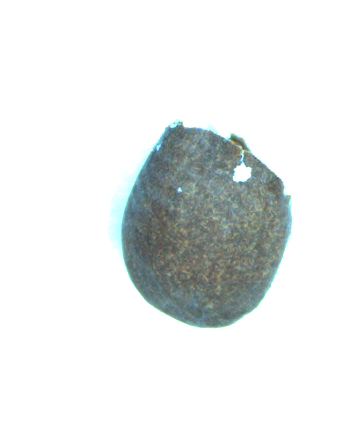

Supplement: S1 Data — (ZIP) [file pone.0273057.s001.zip › Supporting Information/Broken cotton seed/Image_63.bmp]

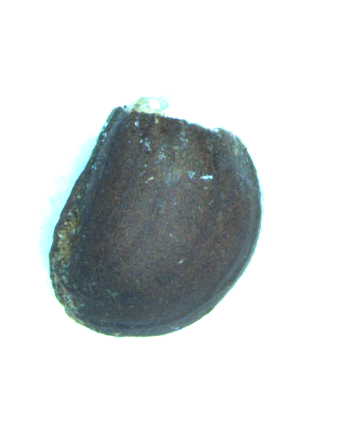

Supplement: S1 Data — (ZIP) [file pone.0273057.s001.zip › Supporting Information/Broken cotton seed/Image_64.bmp]

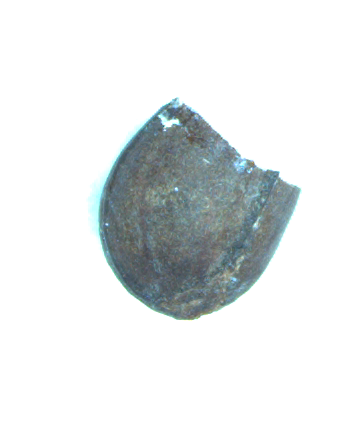

Supplement: S1 Data — (ZIP) [file pone.0273057.s001.zip › Supporting Information/Broken cotton seed/Image_65.bmp]

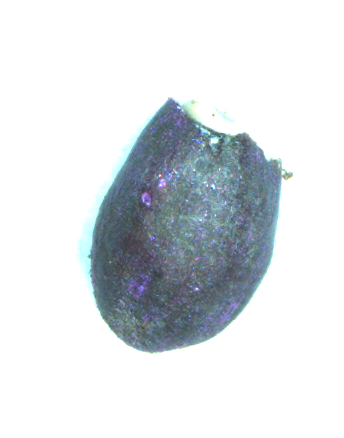

Supplement: S1 Data — (ZIP) [file pone.0273057.s001.zip › Supporting Information/Broken cotton seed/Image_66.bmp]

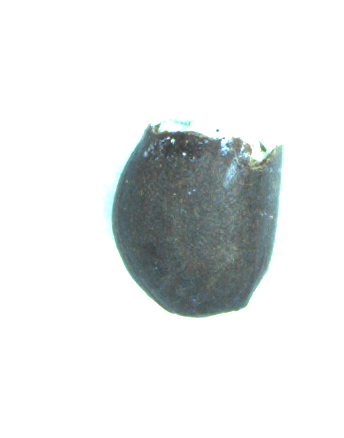

Supplement: S1 Data — (ZIP) [file pone.0273057.s001.zip › Supporting Information/Broken cotton seed/Image_67.bmp]

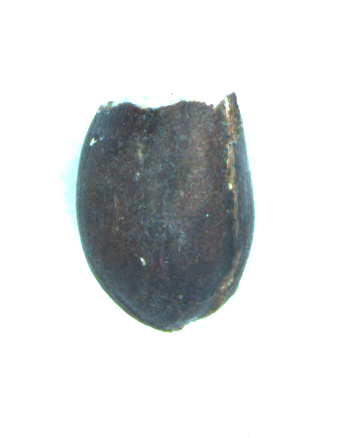

Supplement: S1 Data — (ZIP) [file pone.0273057.s001.zip › Supporting Information/Broken cotton seed/Image_68.bmp]

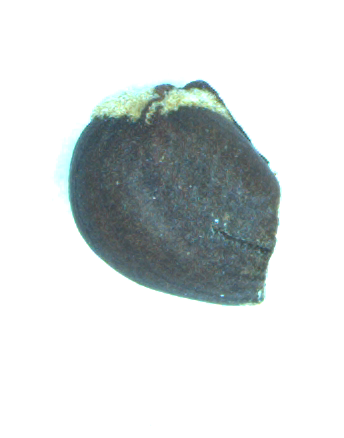

Supplement: S1 Data — (ZIP) [file pone.0273057.s001.zip › Supporting Information/Broken cotton seed/Image_69.bmp]

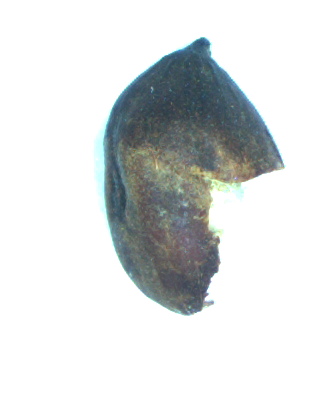

Supplement: S1 Data — (ZIP) [file pone.0273057.s001.zip › Supporting Information/Broken cotton seed/Image_7.bmp]

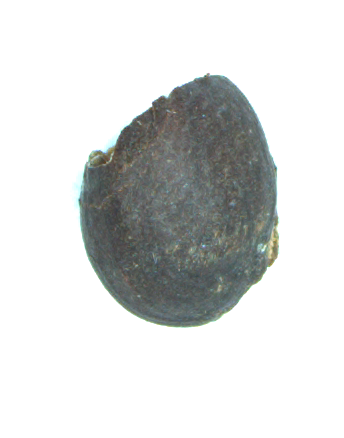

Supplement: S1 Data — (ZIP) [file pone.0273057.s001.zip › Supporting Information/Broken cotton seed/Image_70.bmp]

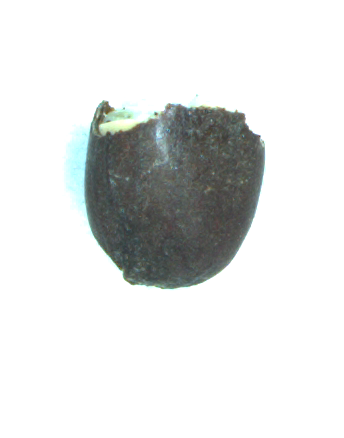

Supplement: S1 Data — (ZIP) [file pone.0273057.s001.zip › Supporting Information/Broken cotton seed/Image_71.bmp]

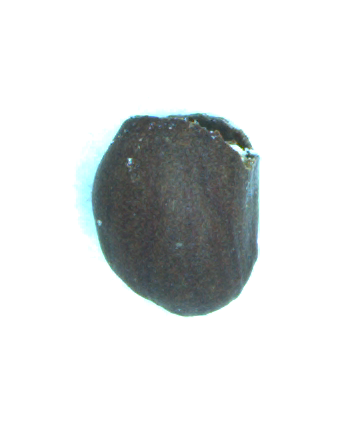

Supplement: S1 Data — (ZIP) [file pone.0273057.s001.zip › Supporting Information/Broken cotton seed/Image_72.bmp]

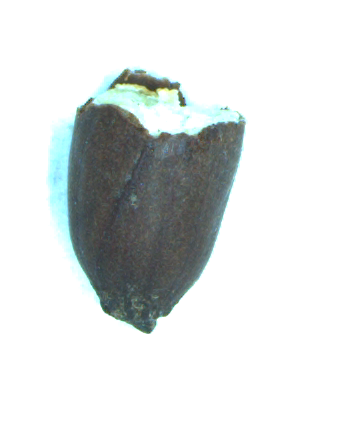

Supplement: S1 Data — (ZIP) [file pone.0273057.s001.zip › Supporting Information/Broken cotton seed/Image_73.bmp]

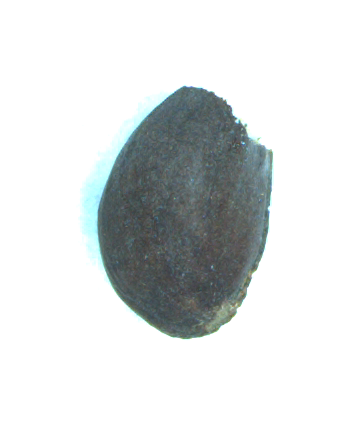

Supplement: S1 Data — (ZIP) [file pone.0273057.s001.zip › Supporting Information/Broken cotton seed/Image_74.bmp]

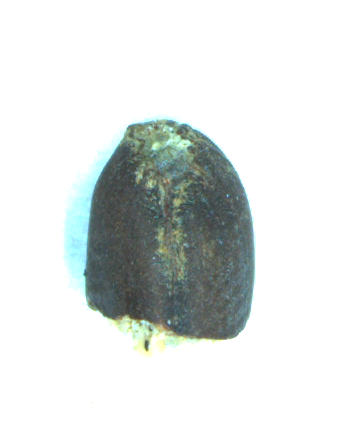

Supplement: S1 Data — (ZIP) [file pone.0273057.s001.zip › Supporting Information/Broken cotton seed/Image_75.bmp]

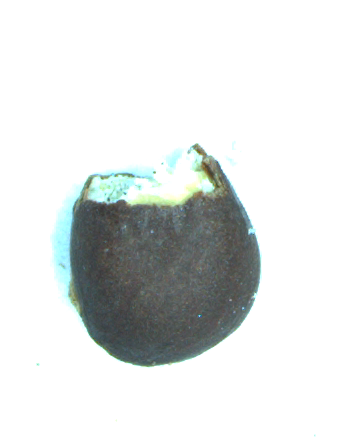

Supplement: S1 Data — (ZIP) [file pone.0273057.s001.zip › Supporting Information/Broken cotton seed/Image_76.bmp]

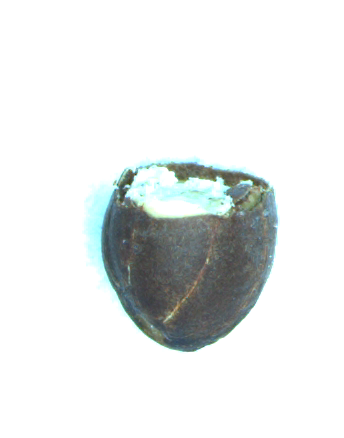

Supplement: S1 Data — (ZIP) [file pone.0273057.s001.zip › Supporting Information/Broken cotton seed/Image_77.bmp]

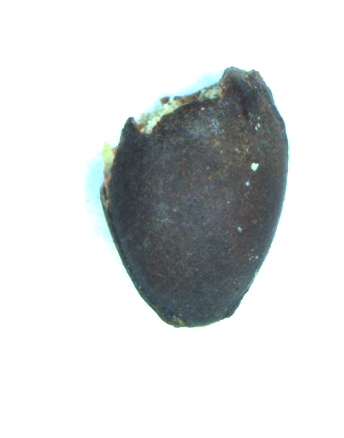

Supplement: S1 Data — (ZIP) [file pone.0273057.s001.zip › Supporting Information/Broken cotton seed/Image_78.bmp]

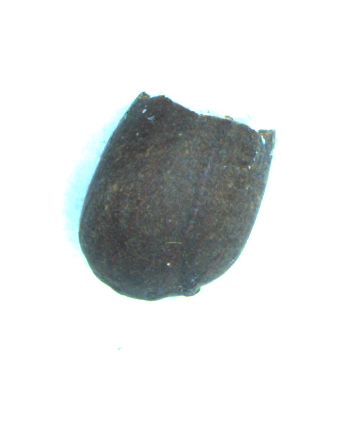

Supplement: S1 Data — (ZIP) [file pone.0273057.s001.zip › Supporting Information/Broken cotton seed/Image_79.bmp]

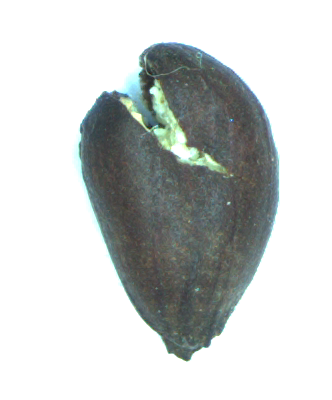

Supplement: S1 Data — (ZIP) [file pone.0273057.s001.zip › Supporting Information/Broken cotton seed/Image_8.bmp]

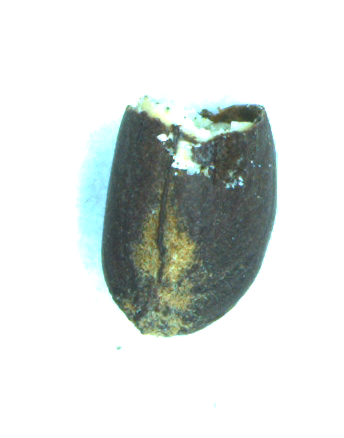

Supplement: S1 Data — (ZIP) [file pone.0273057.s001.zip › Supporting Information/Broken cotton seed/Image_80.bmp]

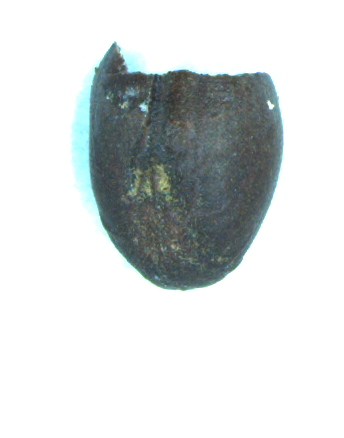

Supplement: S1 Data — (ZIP) [file pone.0273057.s001.zip › Supporting Information/Broken cotton seed/Image_81.bmp]

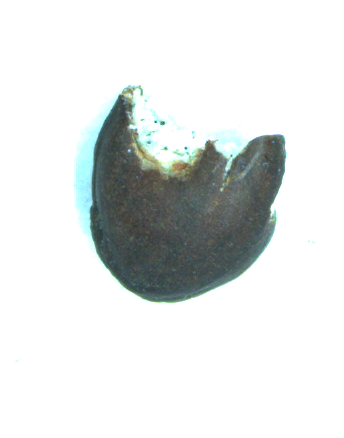

Supplement: S1 Data — (ZIP) [file pone.0273057.s001.zip › Supporting Information/Broken cotton seed/Image_82.bmp]

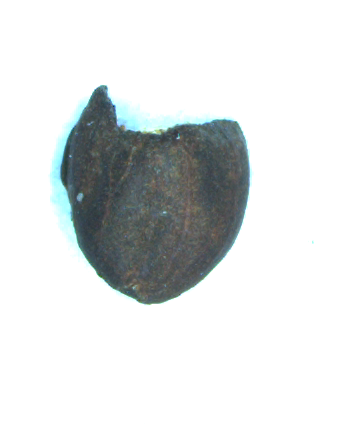

Supplement: S1 Data — (ZIP) [file pone.0273057.s001.zip › Supporting Information/Broken cotton seed/Image_83.bmp]

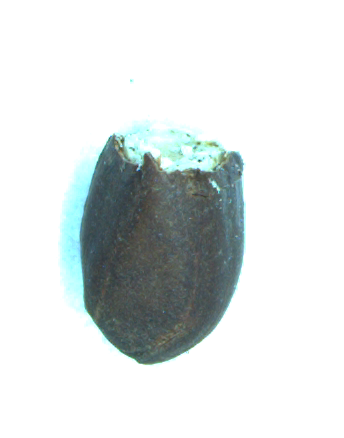

Supplement: S1 Data — (ZIP) [file pone.0273057.s001.zip › Supporting Information/Broken cotton seed/Image_84.bmp]

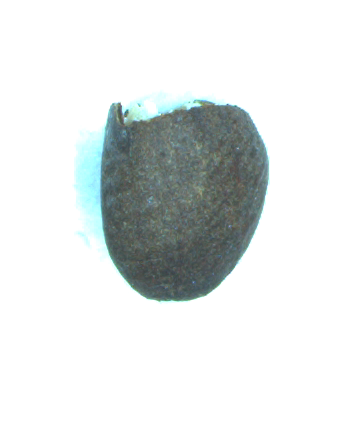

Supplement: S1 Data — (ZIP) [file pone.0273057.s001.zip › Supporting Information/Broken cotton seed/Image_85.bmp]

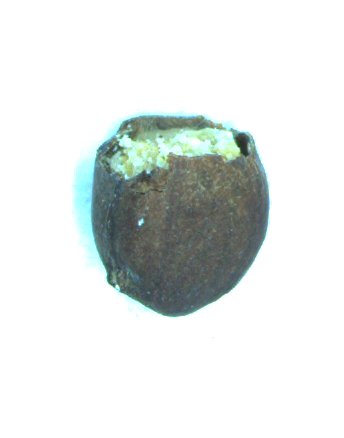

Supplement: S1 Data — (ZIP) [file pone.0273057.s001.zip › Supporting Information/Broken cotton seed/Image_86.bmp]

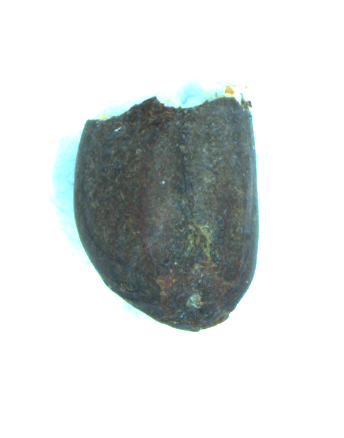

Supplement: S1 Data — (ZIP) [file pone.0273057.s001.zip › Supporting Information/Broken cotton seed/Image_87.bmp]

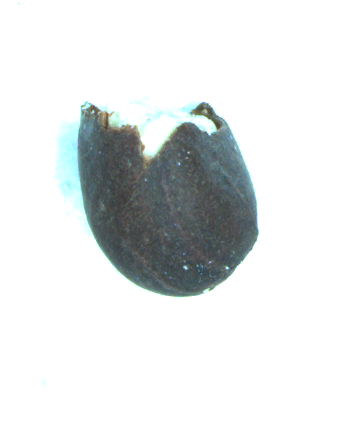

Supplement: S1 Data — (ZIP) [file pone.0273057.s001.zip › Supporting Information/Broken cotton seed/Image_88.bmp]

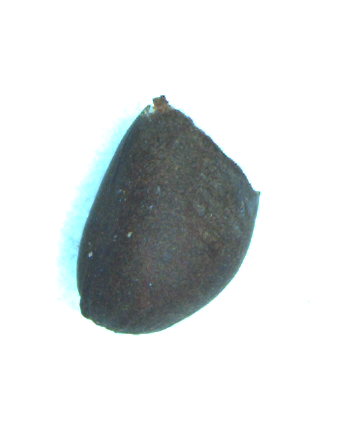

Supplement: S1 Data — (ZIP) [file pone.0273057.s001.zip › Supporting Information/Broken cotton seed/Image_89.bmp]

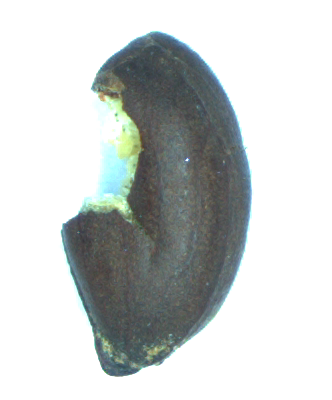

Supplement: S1 Data — (ZIP) [file pone.0273057.s001.zip › Supporting Information/Broken cotton seed/Image_9.bmp]

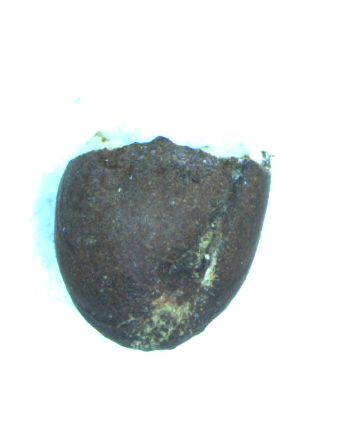

Supplement: S1 Data — (ZIP) [file pone.0273057.s001.zip › Supporting Information/Broken cotton seed/Image_90.bmp]

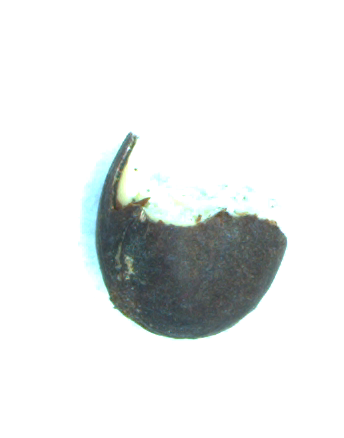

Supplement: S1 Data — (ZIP) [file pone.0273057.s001.zip › Supporting Information/Broken cotton seed/Image_91.bmp]

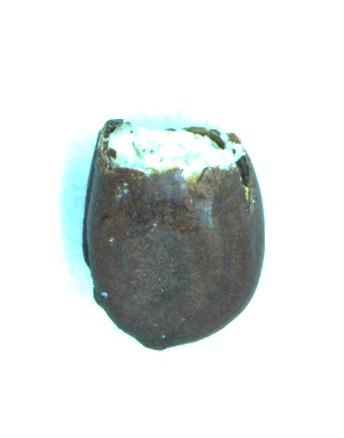

Supplement: S1 Data — (ZIP) [file pone.0273057.s001.zip › Supporting Information/Broken cotton seed/Image_92.bmp]

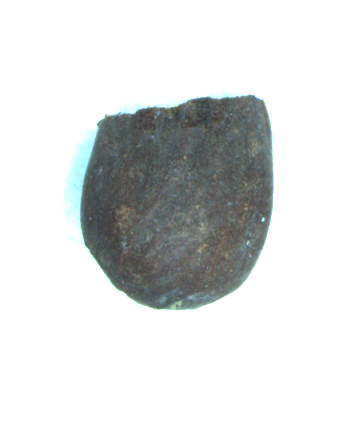

Supplement: S1 Data — (ZIP) [file pone.0273057.s001.zip › Supporting Information/Broken cotton seed/Image_93.bmp]

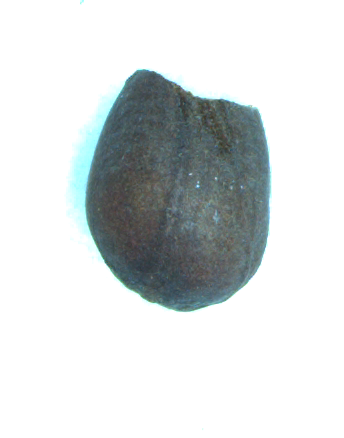

Supplement: S1 Data — (ZIP) [file pone.0273057.s001.zip › Supporting Information/Broken cotton seed/Image_94.bmp]

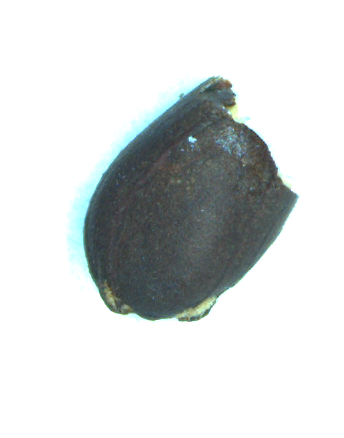

Supplement: S1 Data — (ZIP) [file pone.0273057.s001.zip › Supporting Information/Broken cotton seed/Image_95.bmp]

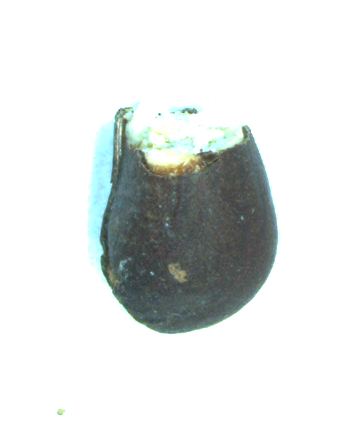

Supplement: S1 Data — (ZIP) [file pone.0273057.s001.zip › Supporting Information/Broken cotton seed/Image_96.bmp]

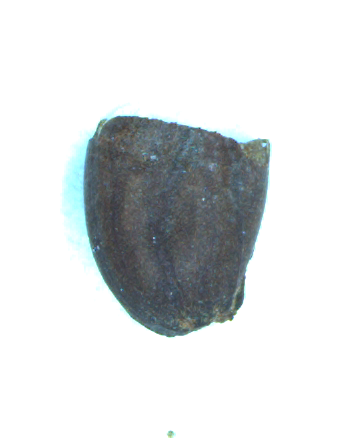

Supplement: S1 Data — (ZIP) [file pone.0273057.s001.zip › Supporting Information/Broken cotton seed/Image_97.bmp]

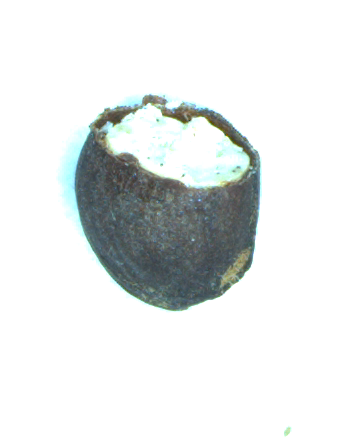

Supplement: S1 Data — (ZIP) [file pone.0273057.s001.zip › Supporting Information/Broken cotton seed/Image_98.bmp]

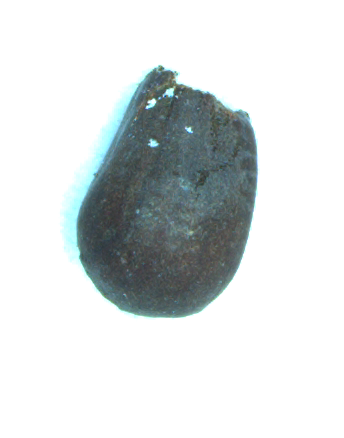

Supplement: S1 Data — (ZIP) [file pone.0273057.s001.zip › Supporting Information/Broken cotton seed/Image_99.bmp]

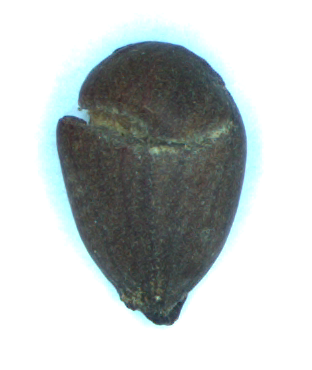

Supplement: S1 Data — (ZIP) [file pone.0273057.s001.zip › Supporting Information/Cracked cotton seed/Image_1000.bmp]

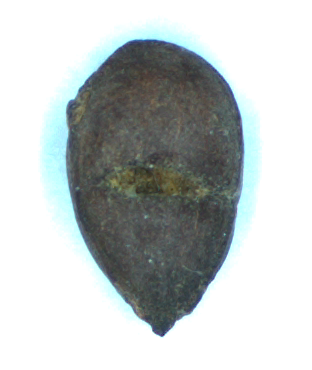

Supplement: S1 Data — (ZIP) [file pone.0273057.s001.zip › Supporting Information/Cracked cotton seed/Image_1001.bmp]

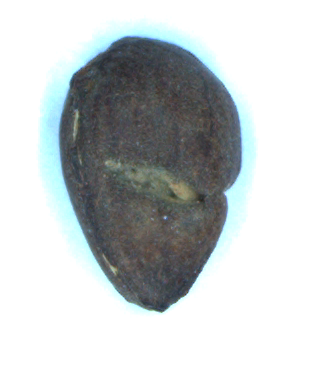

Supplement: S1 Data — (ZIP) [file pone.0273057.s001.zip › Supporting Information/Cracked cotton seed/Image_1002.bmp]

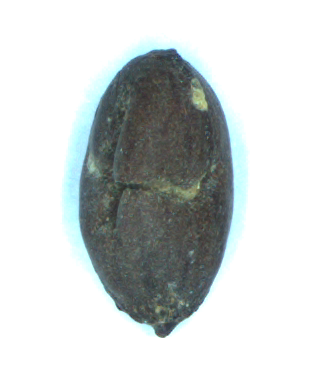

Supplement: S1 Data — (ZIP) [file pone.0273057.s001.zip › Supporting Information/Cracked cotton seed/Image_1003.bmp]

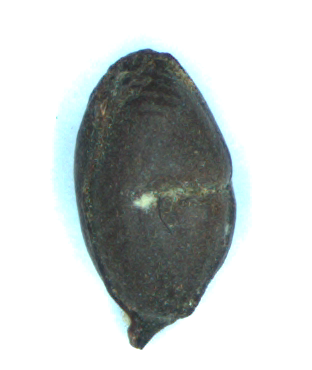

Supplement: S1 Data — (ZIP) [file pone.0273057.s001.zip › Supporting Information/Cracked cotton seed/Image_1004.bmp]

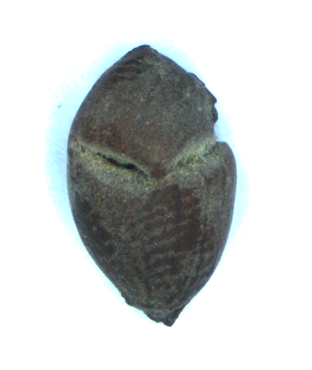

Supplement: S1 Data — (ZIP) [file pone.0273057.s001.zip › Supporting Information/Cracked cotton seed/Image_1005.bmp]

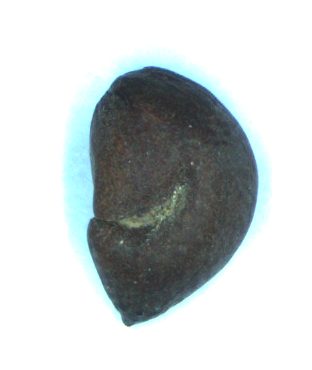

Supplement: S1 Data — (ZIP) [file pone.0273057.s001.zip › Supporting Information/Cracked cotton seed/Image_1006.bmp]

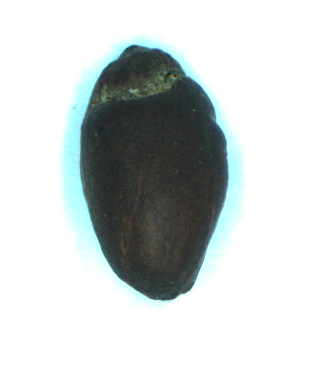

Supplement: S1 Data — (ZIP) [file pone.0273057.s001.zip › Supporting Information/Cracked cotton seed/Image_1007.bmp]

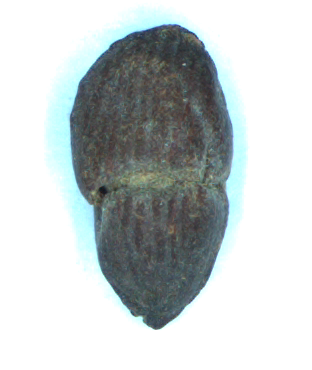

Supplement: S1 Data — (ZIP) [file pone.0273057.s001.zip › Supporting Information/Cracked cotton seed/Image_1008.bmp]

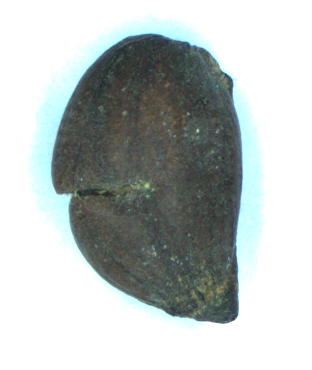

Supplement: S1 Data — (ZIP) [file pone.0273057.s001.zip › Supporting Information/Cracked cotton seed/Image_1009.bmp]

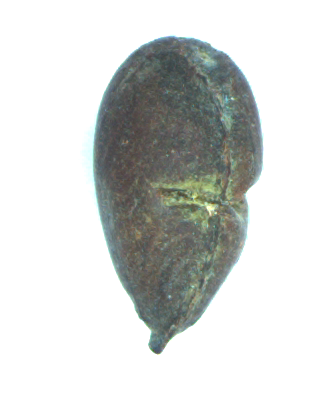

Supplement: S1 Data — (ZIP) [file pone.0273057.s001.zip › Supporting Information/Cracked cotton seed/Image_1010.bmp]

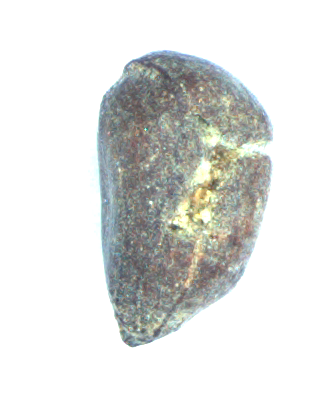

Supplement: S1 Data — (ZIP) [file pone.0273057.s001.zip › Supporting Information/Cracked cotton seed/Image_1011.bmp]

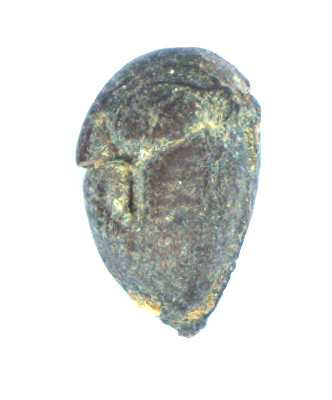

Supplement: S1 Data — (ZIP) [file pone.0273057.s001.zip › Supporting Information/Cracked cotton seed/Image_1012.bmp]

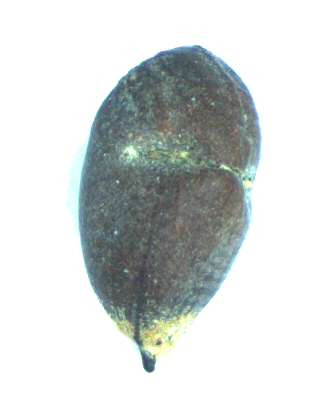

Supplement: S1 Data — (ZIP) [file pone.0273057.s001.zip › Supporting Information/Cracked cotton seed/Image_1013.bmp]

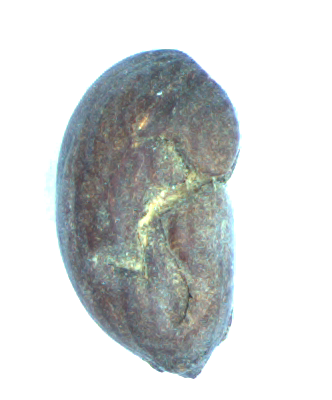

Supplement: S1 Data — (ZIP) [file pone.0273057.s001.zip › Supporting Information/Cracked cotton seed/Image_1014.bmp]

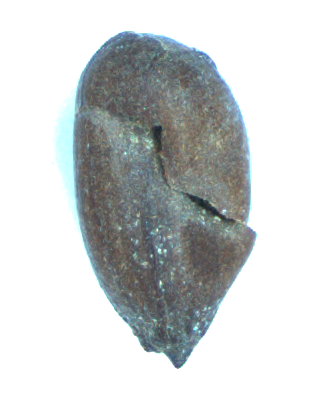

Supplement: S1 Data — (ZIP) [file pone.0273057.s001.zip › Supporting Information/Cracked cotton seed/Image_1015.bmp]

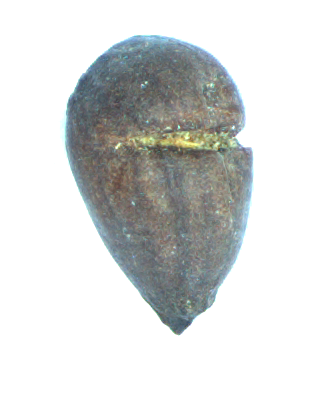

Supplement: S1 Data — (ZIP) [file pone.0273057.s001.zip › Supporting Information/Cracked cotton seed/Image_1016.bmp]

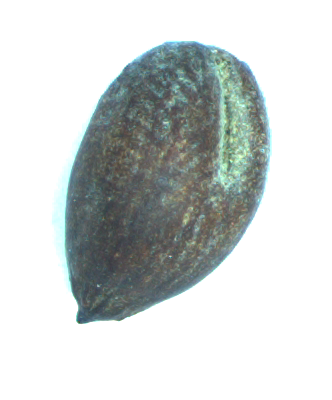

Supplement: S1 Data — (ZIP) [file pone.0273057.s001.zip › Supporting Information/Cracked cotton seed/Image_1017.bmp]

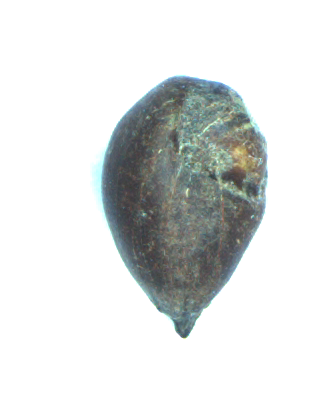

Supplement: S1 Data — (ZIP) [file pone.0273057.s001.zip › Supporting Information/Cracked cotton seed/Image_1018.bmp]

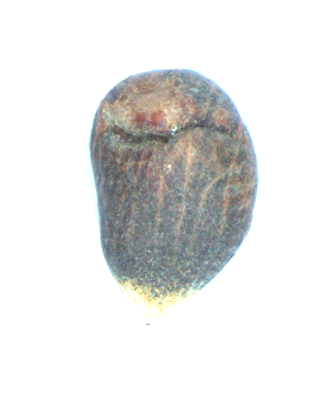

Supplement: S1 Data — (ZIP) [file pone.0273057.s001.zip › Supporting Information/Cracked cotton seed/Image_1019.bmp]

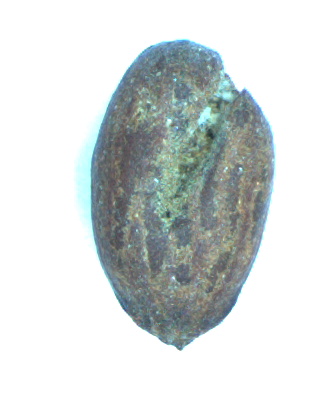

Supplement: S1 Data — (ZIP) [file pone.0273057.s001.zip › Supporting Information/Cracked cotton seed/Image_1020.bmp]

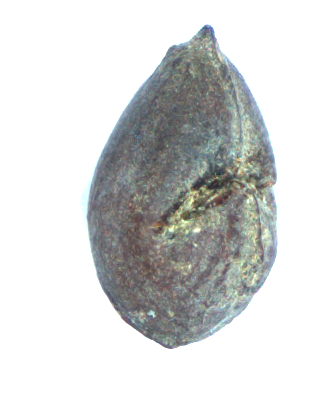

Supplement: S1 Data — (ZIP) [file pone.0273057.s001.zip › Supporting Information/Cracked cotton seed/Image_1021.bmp]

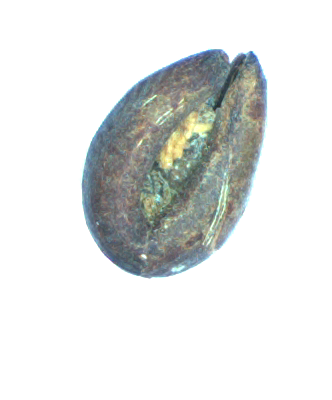

Supplement: S1 Data — (ZIP) [file pone.0273057.s001.zip › Supporting Information/Cracked cotton seed/Image_1022.bmp]

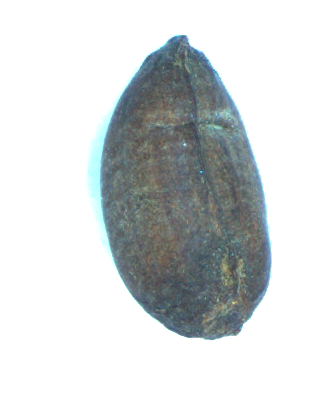

Supplement: S1 Data — (ZIP) [file pone.0273057.s001.zip › Supporting Information/Cracked cotton seed/Image_1023.bmp]

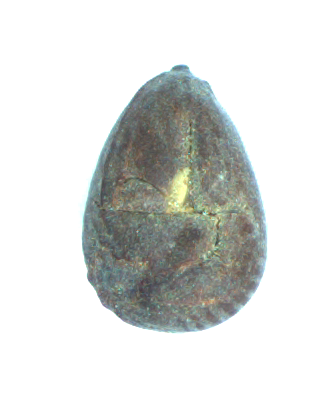

Supplement: S1 Data — (ZIP) [file pone.0273057.s001.zip › Supporting Information/Cracked cotton seed/Image_1024.bmp]

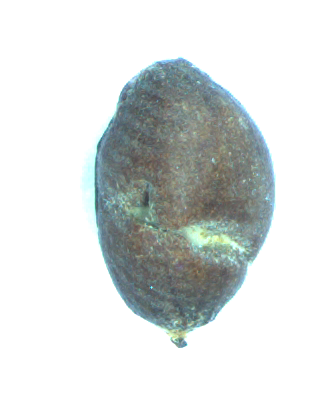

Supplement: S1 Data — (ZIP) [file pone.0273057.s001.zip › Supporting Information/Cracked cotton seed/Image_1025.bmp]

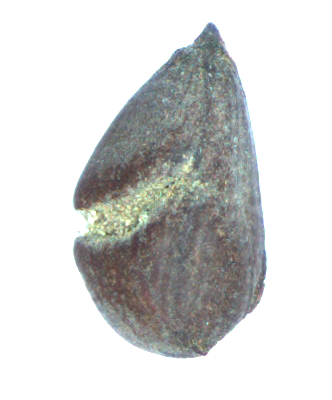

Supplement: S1 Data — (ZIP) [file pone.0273057.s001.zip › Supporting Information/Cracked cotton seed/Image_1026.bmp]

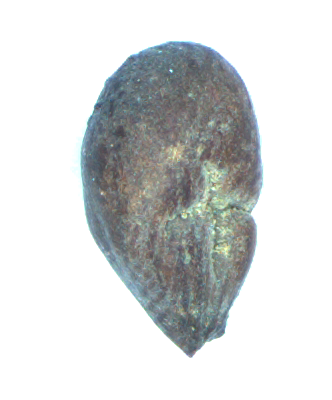

Supplement: S1 Data — (ZIP) [file pone.0273057.s001.zip › Supporting Information/Cracked cotton seed/Image_1027.bmp]

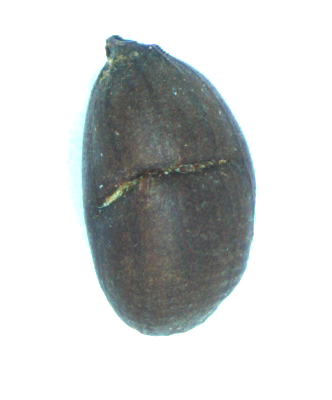

Supplement: S1 Data — (ZIP) [file pone.0273057.s001.zip › Supporting Information/Cracked cotton seed/Image_1028.bmp]

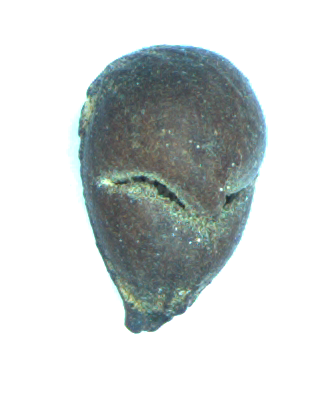

Supplement: S1 Data — (ZIP) [file pone.0273057.s001.zip › Supporting Information/Cracked cotton seed/Image_1029.bmp]

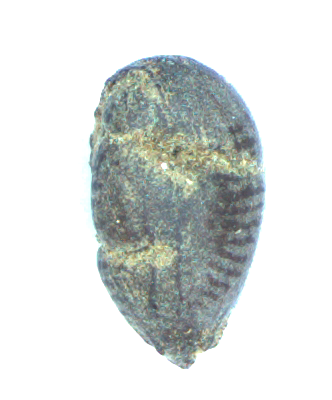

Supplement: S1 Data — (ZIP) [file pone.0273057.s001.zip › Supporting Information/Cracked cotton seed/Image_1030.bmp]

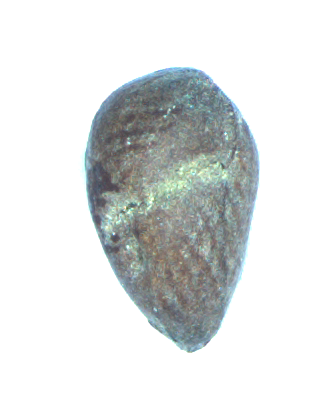

Supplement: S1 Data — (ZIP) [file pone.0273057.s001.zip › Supporting Information/Cracked cotton seed/Image_1031.bmp]

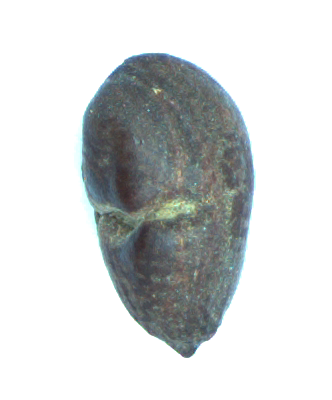

Supplement: S1 Data — (ZIP) [file pone.0273057.s001.zip › Supporting Information/Cracked cotton seed/Image_1032.bmp]

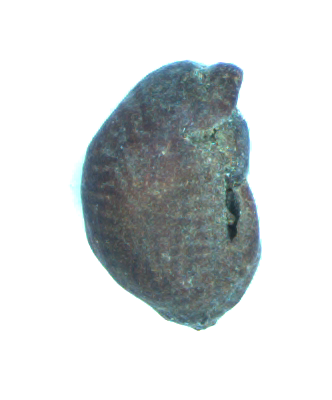

Supplement: S1 Data — (ZIP) [file pone.0273057.s001.zip › Supporting Information/Cracked cotton seed/Image_1033.bmp]

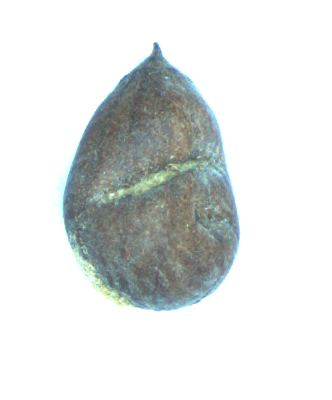

Supplement: S1 Data — (ZIP) [file pone.0273057.s001.zip › Supporting Information/Cracked cotton seed/Image_1034.bmp]

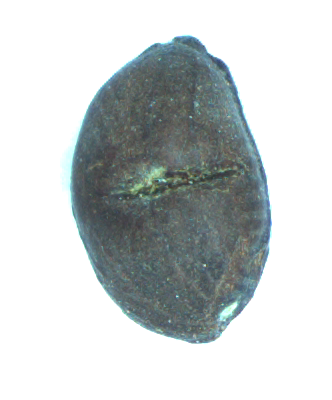

Supplement: S1 Data — (ZIP) [file pone.0273057.s001.zip › Supporting Information/Cracked cotton seed/Image_1035.bmp]

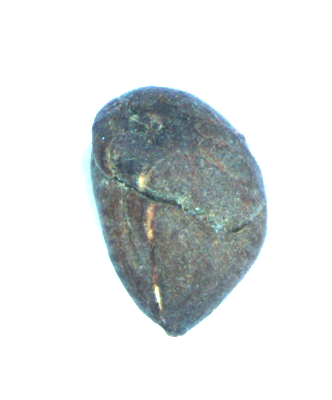

Supplement: S1 Data — (ZIP) [file pone.0273057.s001.zip › Supporting Information/Cracked cotton seed/Image_1036.bmp]

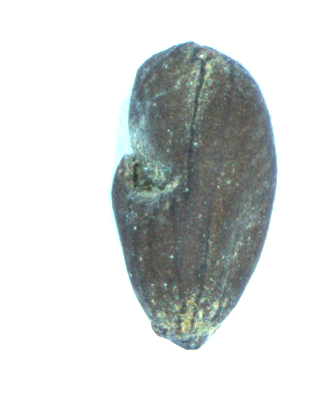

Supplement: S1 Data — (ZIP) [file pone.0273057.s001.zip › Supporting Information/Cracked cotton seed/Image_1037.bmp]

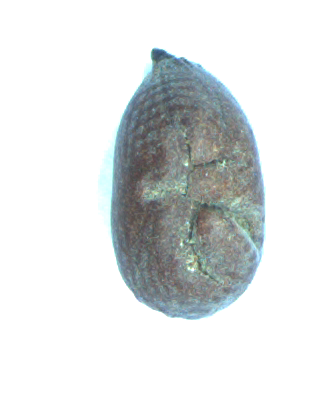

Supplement: S1 Data — (ZIP) [file pone.0273057.s001.zip › Supporting Information/Cracked cotton seed/Image_1038.bmp]

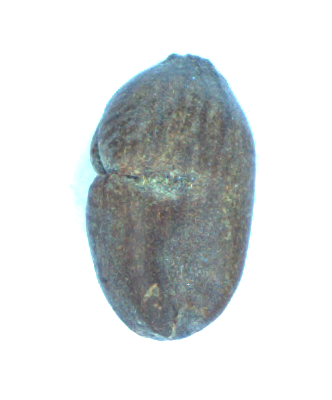

Supplement: S1 Data — (ZIP) [file pone.0273057.s001.zip › Supporting Information/Cracked cotton seed/Image_1039.bmp]

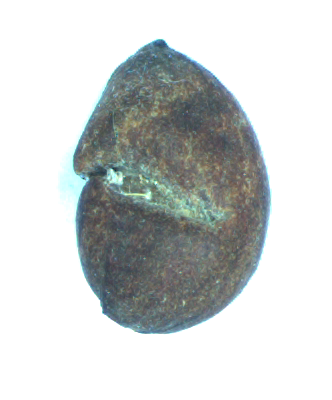

Supplement: S1 Data — (ZIP) [file pone.0273057.s001.zip › Supporting Information/Cracked cotton seed/Image_1040.bmp]

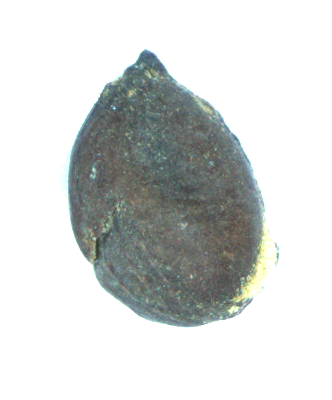

Supplement: S1 Data — (ZIP) [file pone.0273057.s001.zip › Supporting Information/Cracked cotton seed/Image_1041.bmp]

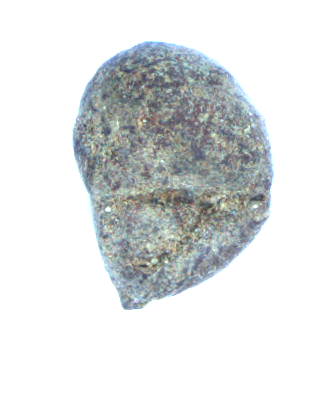

Supplement: S1 Data — (ZIP) [file pone.0273057.s001.zip › Supporting Information/Cracked cotton seed/Image_1042.bmp]

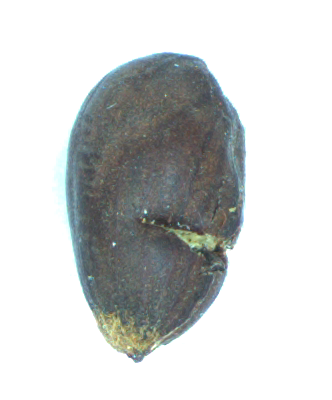

Supplement: S1 Data — (ZIP) [file pone.0273057.s001.zip › Supporting Information/Cracked cotton seed/Image_1043.bmp]

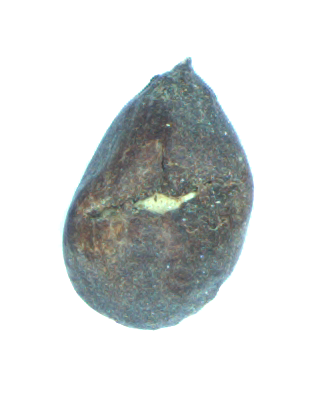

Supplement: S1 Data — (ZIP) [file pone.0273057.s001.zip › Supporting Information/Cracked cotton seed/Image_1044.bmp]

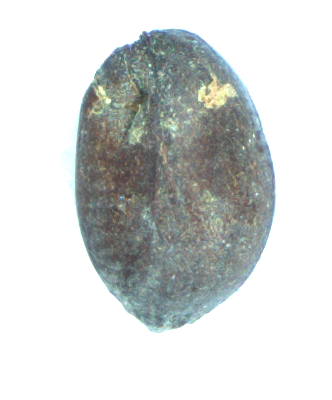

Supplement: S1 Data — (ZIP) [file pone.0273057.s001.zip › Supporting Information/Cracked cotton seed/Image_1045.bmp]

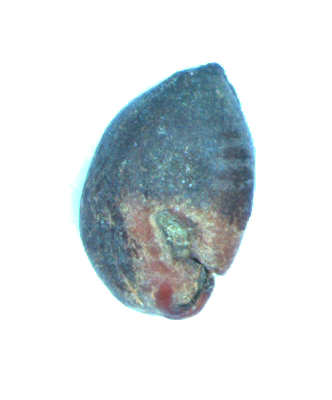

Supplement: S1 Data — (ZIP) [file pone.0273057.s001.zip › Supporting Information/Cracked cotton seed/Image_1046.bmp]

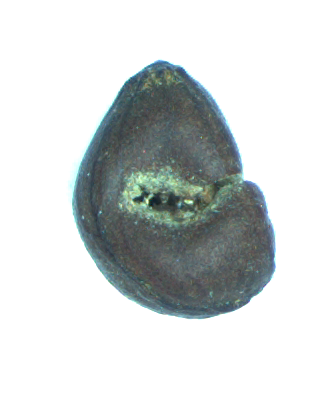

Supplement: S1 Data — (ZIP) [file pone.0273057.s001.zip › Supporting Information/Cracked cotton seed/Image_1047.bmp]

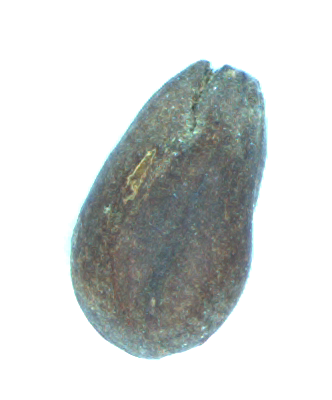

Supplement: S1 Data — (ZIP) [file pone.0273057.s001.zip › Supporting Information/Cracked cotton seed/Image_1048.bmp]

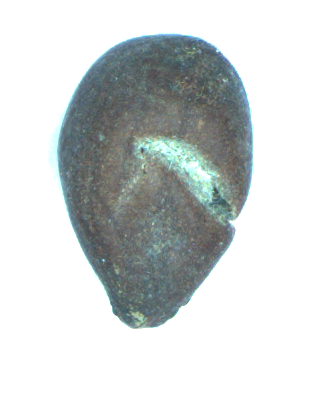

Supplement: S1 Data — (ZIP) [file pone.0273057.s001.zip › Supporting Information/Cracked cotton seed/Image_1049.bmp]
