# Supplementary figures and images for: A method for detecting the quality of cotton seeds based on an improved ResNet50 model (part 1 of 2)
Source: PLoS One. 2023 Feb 15;18(2):e0273057. doi: 10.1371/journal.pone.0273057 (PMC9931132; doi:10.1371/journal.pone.0273057)

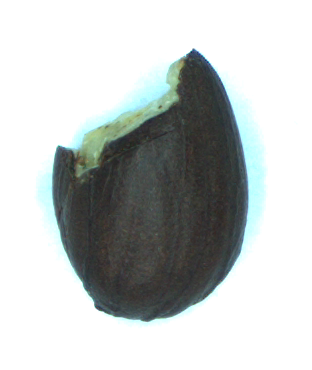

Supplement: S1 Data — (ZIP) [file pone.0273057.s001.zip › Supporting Information/Broken cotton seed/Image_1.bmp]

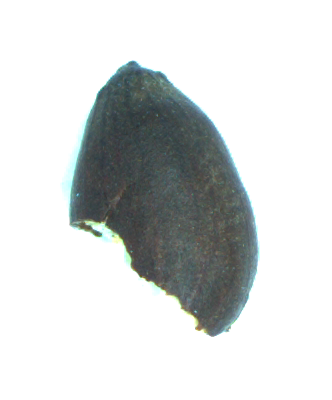

Supplement: S1 Data — (ZIP) [file pone.0273057.s001.zip › Supporting Information/Broken cotton seed/Image_10.bmp]

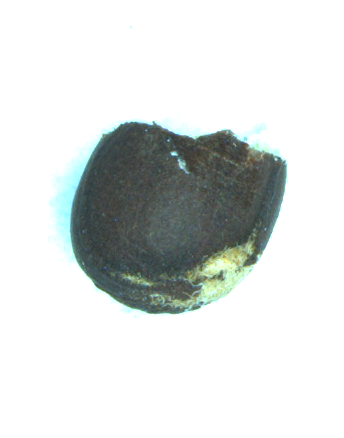

Supplement: S1 Data — (ZIP) [file pone.0273057.s001.zip › Supporting Information/Broken cotton seed/Image_100.bmp]

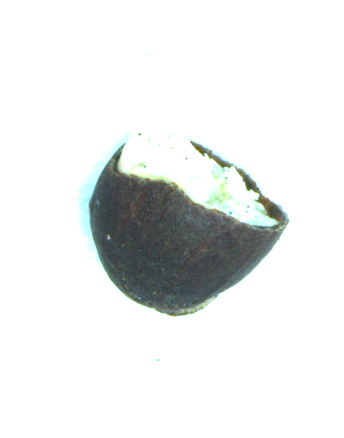

Supplement: S1 Data — (ZIP) [file pone.0273057.s001.zip › Supporting Information/Broken cotton seed/Image_101.bmp]

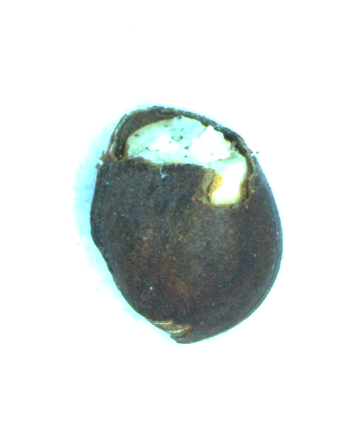

Supplement: S1 Data — (ZIP) [file pone.0273057.s001.zip › Supporting Information/Broken cotton seed/Image_102.bmp]

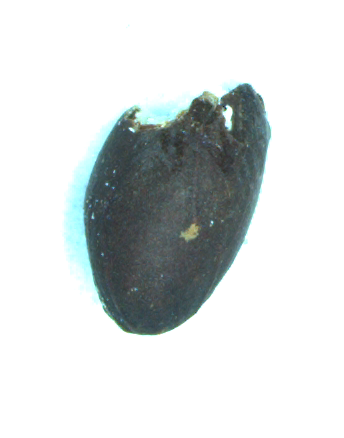

Supplement: S1 Data — (ZIP) [file pone.0273057.s001.zip › Supporting Information/Broken cotton seed/Image_103.bmp]

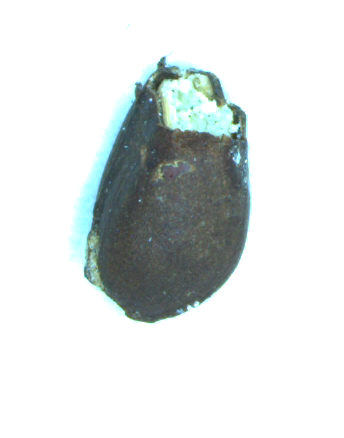

Supplement: S1 Data — (ZIP) [file pone.0273057.s001.zip › Supporting Information/Broken cotton seed/Image_104.bmp]

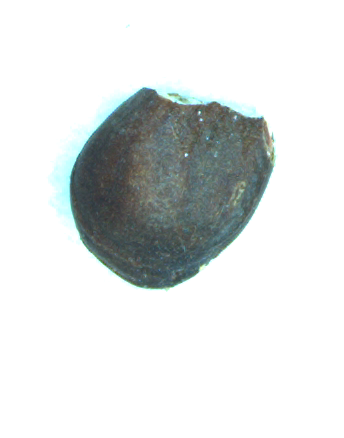

Supplement: S1 Data — (ZIP) [file pone.0273057.s001.zip › Supporting Information/Broken cotton seed/Image_105.bmp]

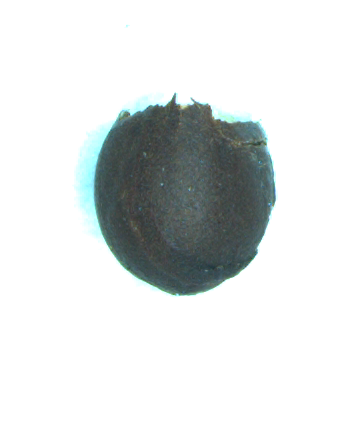

Supplement: S1 Data — (ZIP) [file pone.0273057.s001.zip › Supporting Information/Broken cotton seed/Image_106.bmp]

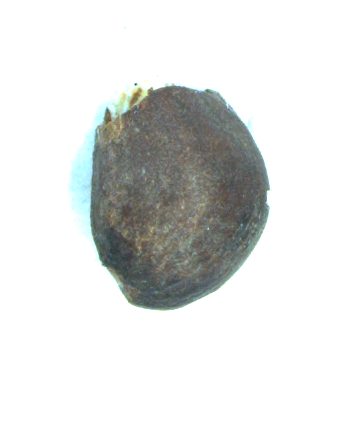

Supplement: S1 Data — (ZIP) [file pone.0273057.s001.zip › Supporting Information/Broken cotton seed/Image_107.bmp]

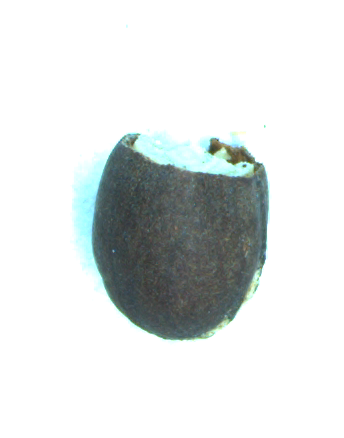

Supplement: S1 Data — (ZIP) [file pone.0273057.s001.zip › Supporting Information/Broken cotton seed/Image_108.bmp]

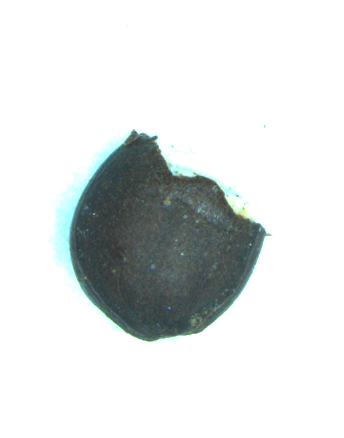

Supplement: S1 Data — (ZIP) [file pone.0273057.s001.zip › Supporting Information/Broken cotton seed/Image_109.bmp]

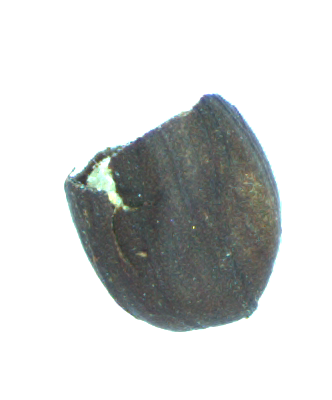

Supplement: S1 Data — (ZIP) [file pone.0273057.s001.zip › Supporting Information/Broken cotton seed/Image_11.bmp]

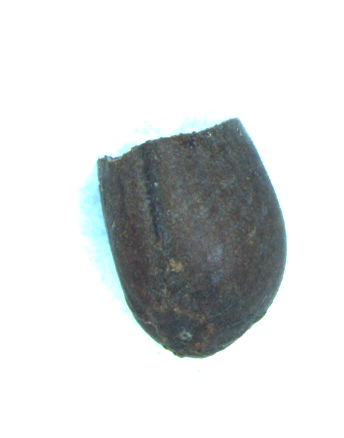

Supplement: S1 Data — (ZIP) [file pone.0273057.s001.zip › Supporting Information/Broken cotton seed/Image_110.bmp]

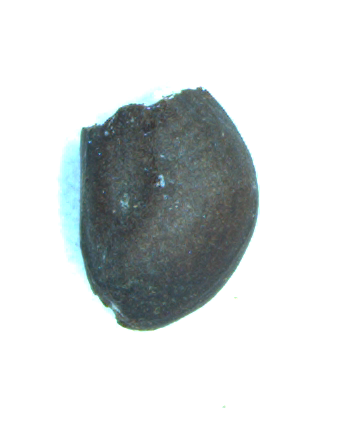

Supplement: S1 Data — (ZIP) [file pone.0273057.s001.zip › Supporting Information/Broken cotton seed/Image_111.bmp]

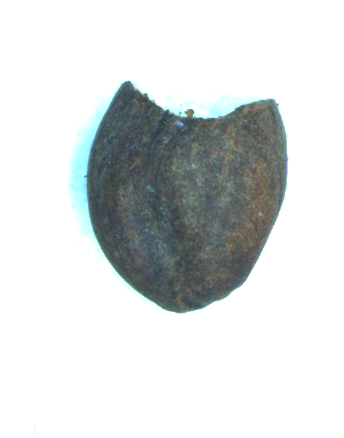

Supplement: S1 Data — (ZIP) [file pone.0273057.s001.zip › Supporting Information/Broken cotton seed/Image_112.bmp]

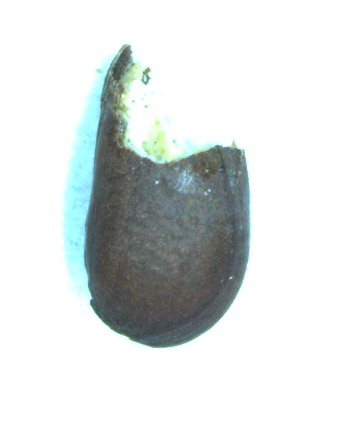

Supplement: S1 Data — (ZIP) [file pone.0273057.s001.zip › Supporting Information/Broken cotton seed/Image_113.bmp]

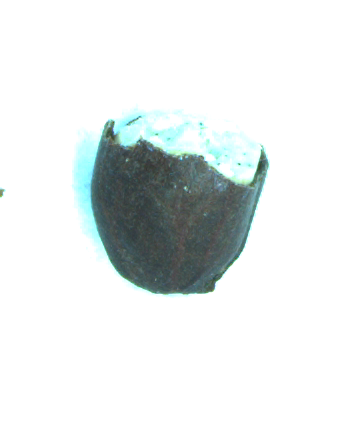

Supplement: S1 Data — (ZIP) [file pone.0273057.s001.zip › Supporting Information/Broken cotton seed/Image_114.bmp]

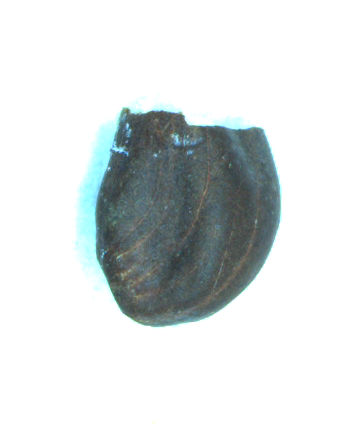

Supplement: S1 Data — (ZIP) [file pone.0273057.s001.zip › Supporting Information/Broken cotton seed/Image_115.bmp]

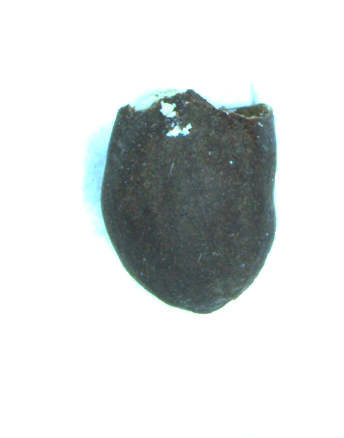

Supplement: S1 Data — (ZIP) [file pone.0273057.s001.zip › Supporting Information/Broken cotton seed/Image_116.bmp]

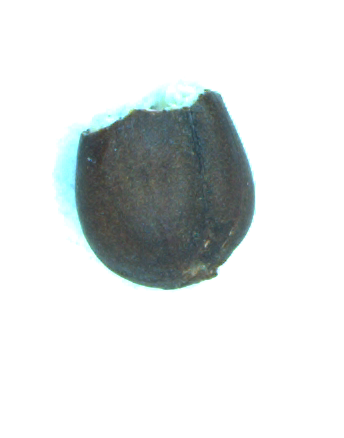

Supplement: S1 Data — (ZIP) [file pone.0273057.s001.zip › Supporting Information/Broken cotton seed/Image_117.bmp]

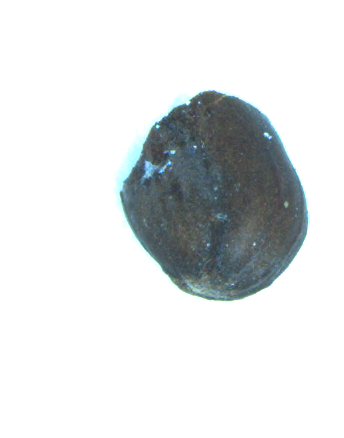

Supplement: S1 Data — (ZIP) [file pone.0273057.s001.zip › Supporting Information/Broken cotton seed/Image_118.bmp]

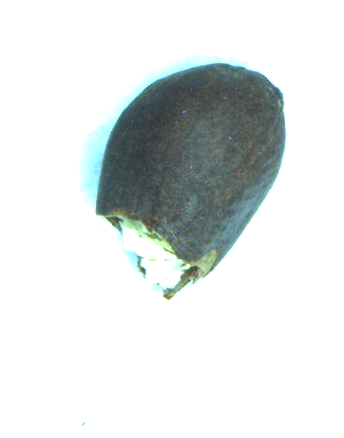

Supplement: S1 Data — (ZIP) [file pone.0273057.s001.zip › Supporting Information/Broken cotton seed/Image_119.bmp]

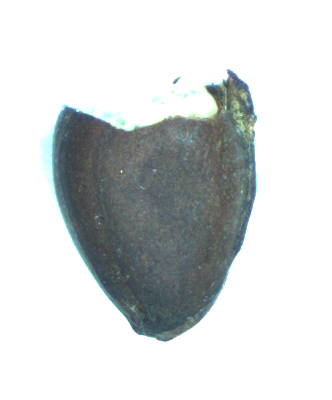

Supplement: S1 Data — (ZIP) [file pone.0273057.s001.zip › Supporting Information/Broken cotton seed/Image_12.bmp]

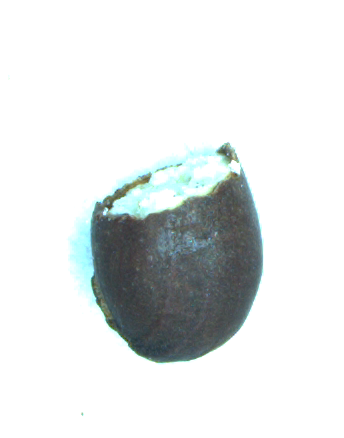

Supplement: S1 Data — (ZIP) [file pone.0273057.s001.zip › Supporting Information/Broken cotton seed/Image_120.bmp]

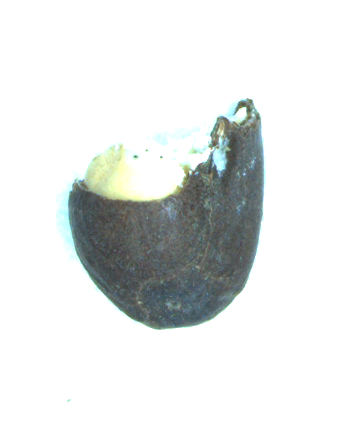

Supplement: S1 Data — (ZIP) [file pone.0273057.s001.zip › Supporting Information/Broken cotton seed/Image_121.bmp]

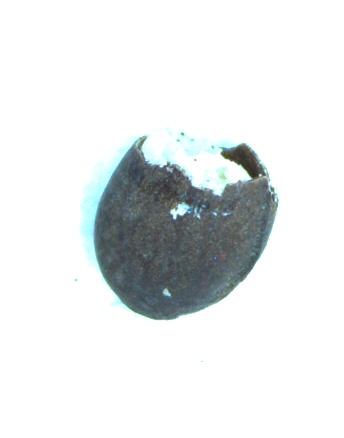

Supplement: S1 Data — (ZIP) [file pone.0273057.s001.zip › Supporting Information/Broken cotton seed/Image_122.bmp]

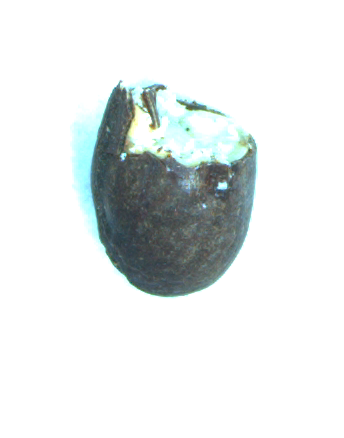

Supplement: S1 Data — (ZIP) [file pone.0273057.s001.zip › Supporting Information/Broken cotton seed/Image_123.bmp]

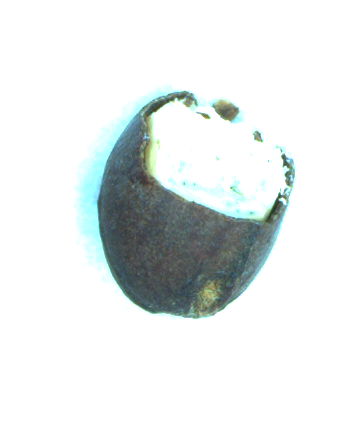

Supplement: S1 Data — (ZIP) [file pone.0273057.s001.zip › Supporting Information/Broken cotton seed/Image_124.bmp]

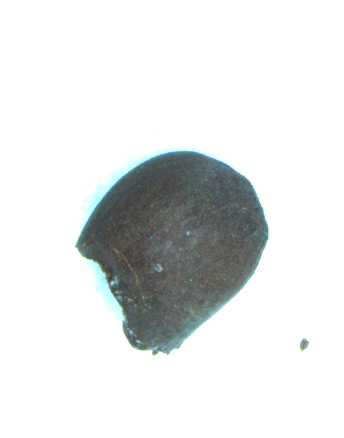

Supplement: S1 Data — (ZIP) [file pone.0273057.s001.zip › Supporting Information/Broken cotton seed/Image_125.bmp]

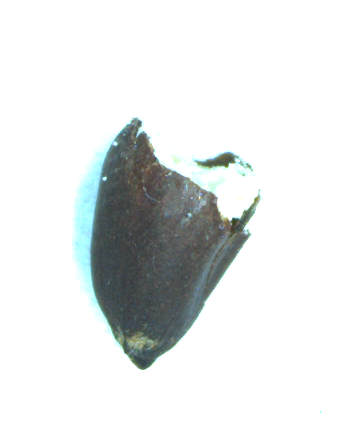

Supplement: S1 Data — (ZIP) [file pone.0273057.s001.zip › Supporting Information/Broken cotton seed/Image_126.bmp]

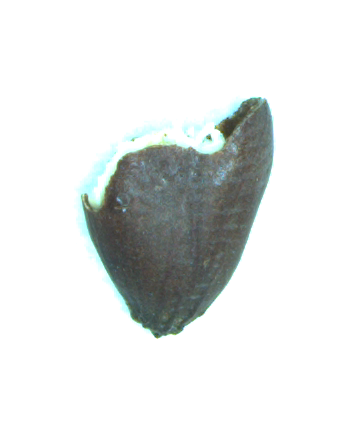

Supplement: S1 Data — (ZIP) [file pone.0273057.s001.zip › Supporting Information/Broken cotton seed/Image_127.bmp]

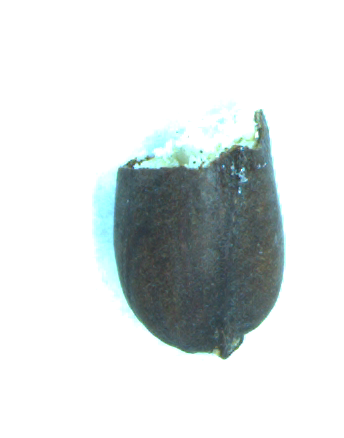

Supplement: S1 Data — (ZIP) [file pone.0273057.s001.zip › Supporting Information/Broken cotton seed/Image_128.bmp]

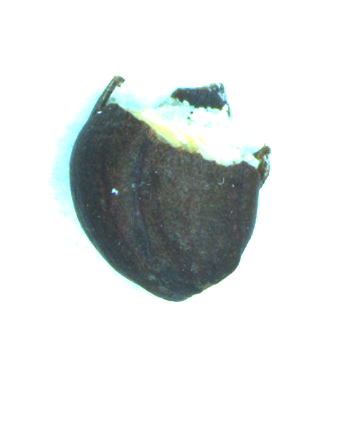

Supplement: S1 Data — (ZIP) [file pone.0273057.s001.zip › Supporting Information/Broken cotton seed/Image_129.bmp]

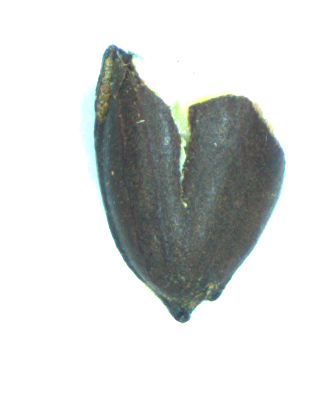

Supplement: S1 Data — (ZIP) [file pone.0273057.s001.zip › Supporting Information/Broken cotton seed/Image_13.bmp]

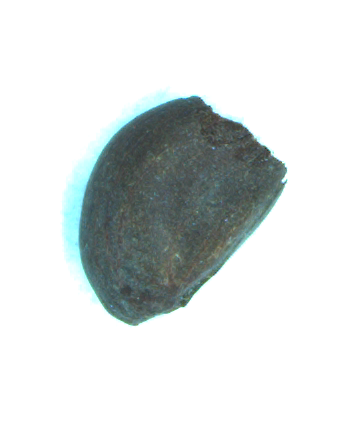

Supplement: S1 Data — (ZIP) [file pone.0273057.s001.zip › Supporting Information/Broken cotton seed/Image_130.bmp]

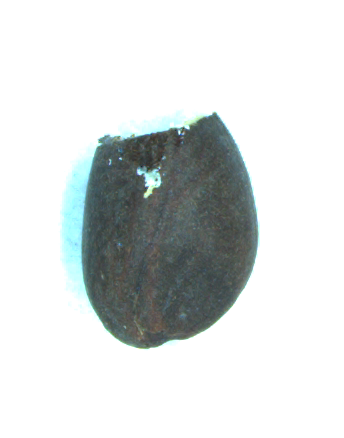

Supplement: S1 Data — (ZIP) [file pone.0273057.s001.zip › Supporting Information/Broken cotton seed/Image_131.bmp]

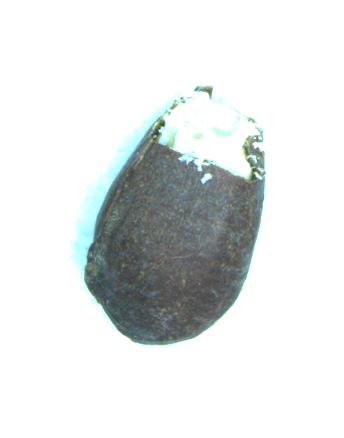

Supplement: S1 Data — (ZIP) [file pone.0273057.s001.zip › Supporting Information/Broken cotton seed/Image_132.bmp]

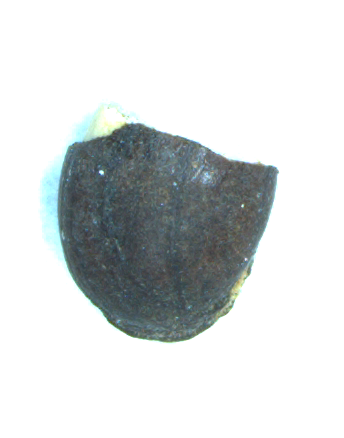

Supplement: S1 Data — (ZIP) [file pone.0273057.s001.zip › Supporting Information/Broken cotton seed/Image_133.bmp]

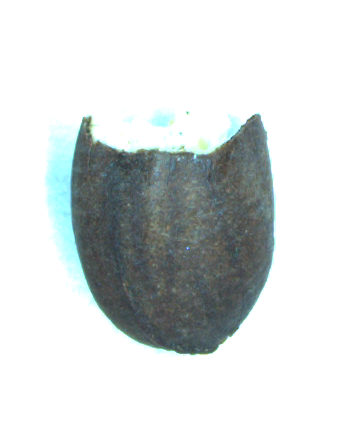

Supplement: S1 Data — (ZIP) [file pone.0273057.s001.zip › Supporting Information/Broken cotton seed/Image_134.bmp]

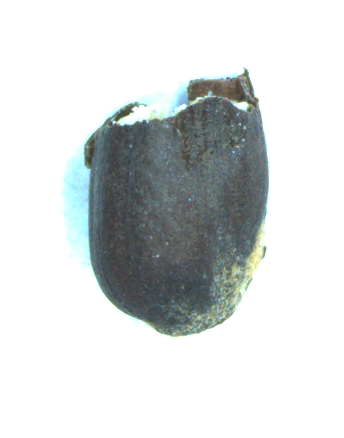

Supplement: S1 Data — (ZIP) [file pone.0273057.s001.zip › Supporting Information/Broken cotton seed/Image_135.bmp]

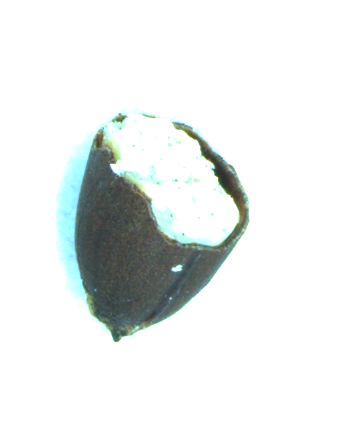

Supplement: S1 Data — (ZIP) [file pone.0273057.s001.zip › Supporting Information/Broken cotton seed/Image_136.bmp]

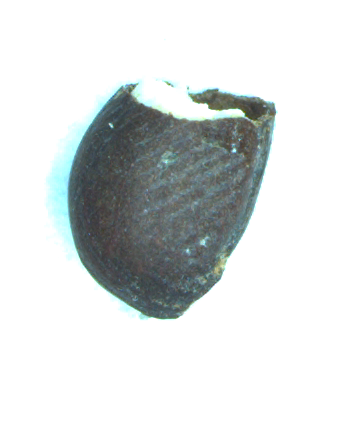

Supplement: S1 Data — (ZIP) [file pone.0273057.s001.zip › Supporting Information/Broken cotton seed/Image_137.bmp]

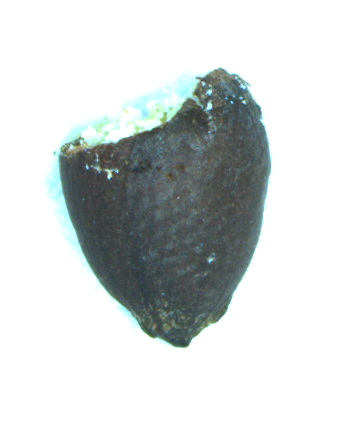

Supplement: S1 Data — (ZIP) [file pone.0273057.s001.zip › Supporting Information/Broken cotton seed/Image_138.bmp]

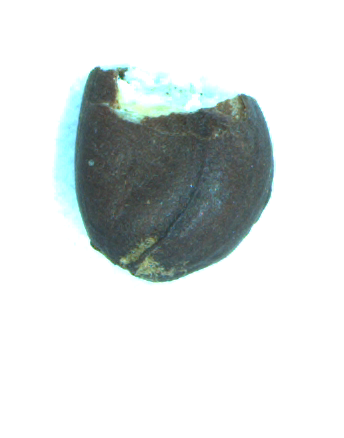

Supplement: S1 Data — (ZIP) [file pone.0273057.s001.zip › Supporting Information/Broken cotton seed/Image_139.bmp]

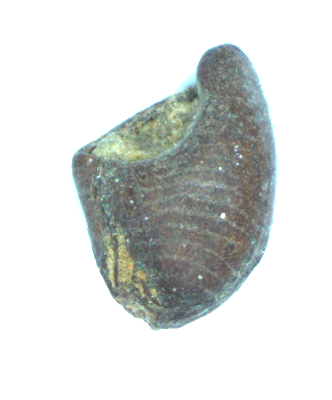

Supplement: S1 Data — (ZIP) [file pone.0273057.s001.zip › Supporting Information/Broken cotton seed/Image_14.bmp]

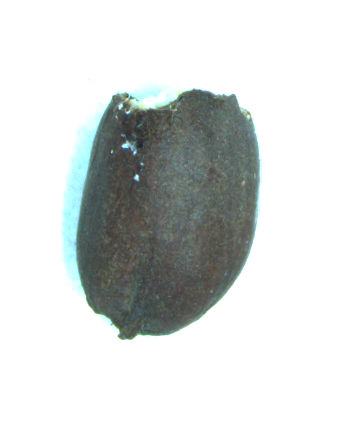

Supplement: S1 Data — (ZIP) [file pone.0273057.s001.zip › Supporting Information/Broken cotton seed/Image_140.bmp]

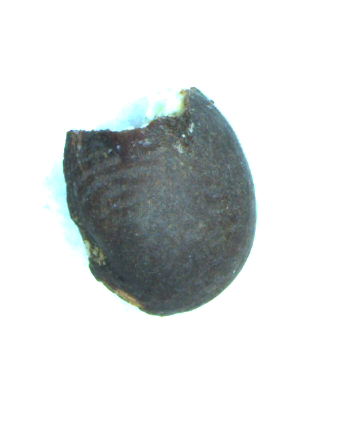

Supplement: S1 Data — (ZIP) [file pone.0273057.s001.zip › Supporting Information/Broken cotton seed/Image_141.bmp]

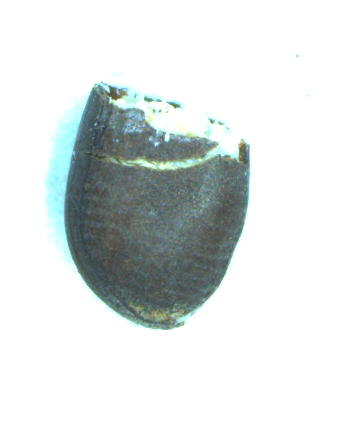

Supplement: S1 Data — (ZIP) [file pone.0273057.s001.zip › Supporting Information/Broken cotton seed/Image_142.bmp]

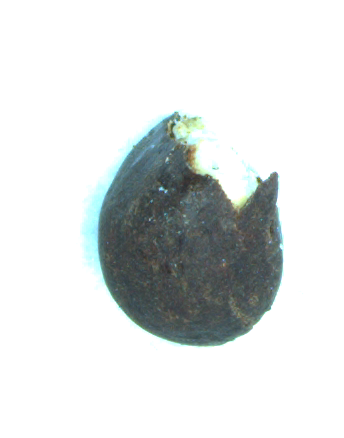

Supplement: S1 Data — (ZIP) [file pone.0273057.s001.zip › Supporting Information/Broken cotton seed/Image_143.bmp]

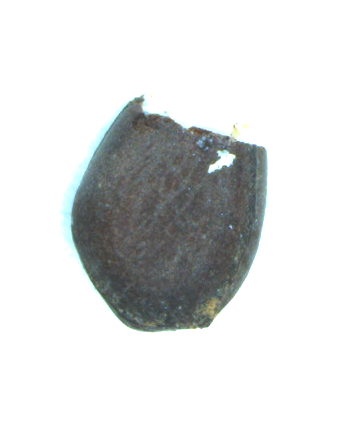

Supplement: S1 Data — (ZIP) [file pone.0273057.s001.zip › Supporting Information/Broken cotton seed/Image_144.bmp]

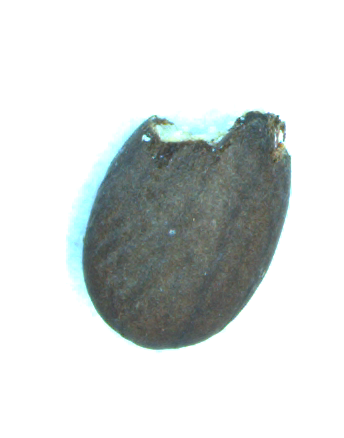

Supplement: S1 Data — (ZIP) [file pone.0273057.s001.zip › Supporting Information/Broken cotton seed/Image_145.bmp]

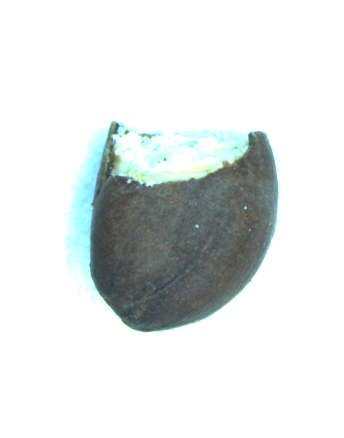

Supplement: S1 Data — (ZIP) [file pone.0273057.s001.zip › Supporting Information/Broken cotton seed/Image_146.bmp]

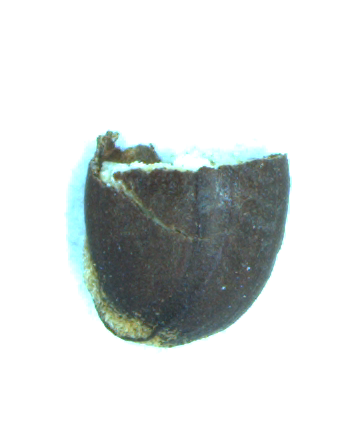

Supplement: S1 Data — (ZIP) [file pone.0273057.s001.zip › Supporting Information/Broken cotton seed/Image_147.bmp]

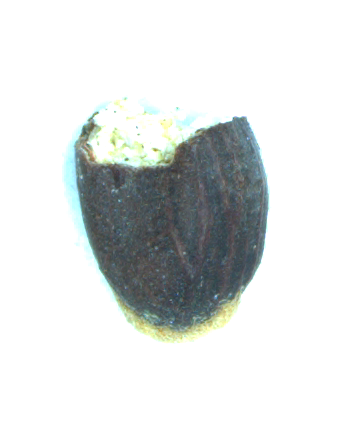

Supplement: S1 Data — (ZIP) [file pone.0273057.s001.zip › Supporting Information/Broken cotton seed/Image_148.bmp]

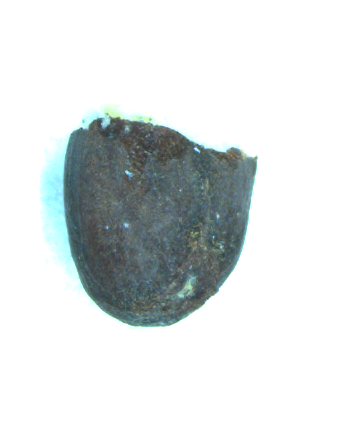

Supplement: S1 Data — (ZIP) [file pone.0273057.s001.zip › Supporting Information/Broken cotton seed/Image_149.bmp]

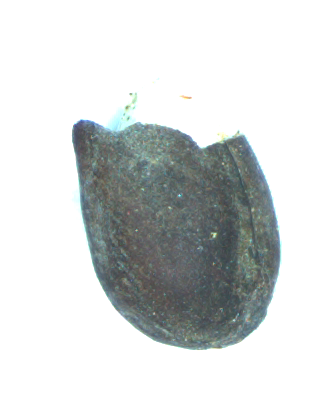

Supplement: S1 Data — (ZIP) [file pone.0273057.s001.zip › Supporting Information/Broken cotton seed/Image_15.bmp]

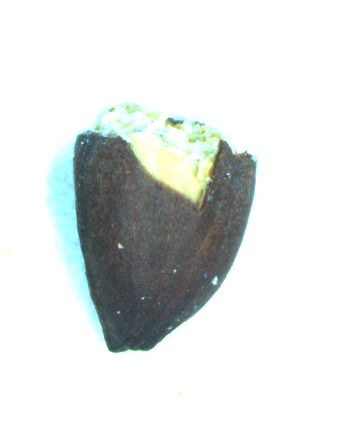

Supplement: S1 Data — (ZIP) [file pone.0273057.s001.zip › Supporting Information/Broken cotton seed/Image_150.bmp]

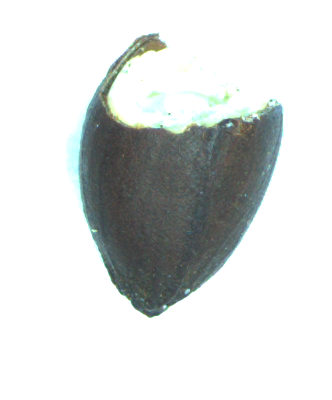

Supplement: S1 Data — (ZIP) [file pone.0273057.s001.zip › Supporting Information/Broken cotton seed/Image_16.bmp]

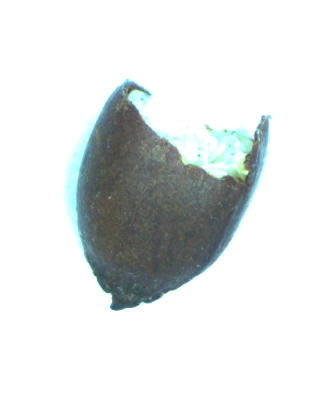

Supplement: S1 Data — (ZIP) [file pone.0273057.s001.zip › Supporting Information/Broken cotton seed/Image_17.bmp]

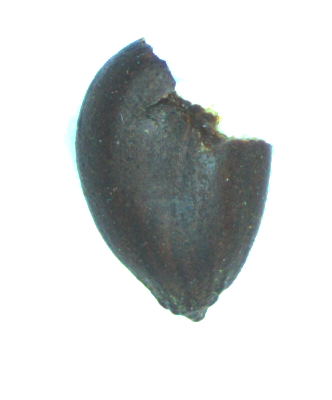

Supplement: S1 Data — (ZIP) [file pone.0273057.s001.zip › Supporting Information/Broken cotton seed/Image_18.bmp]

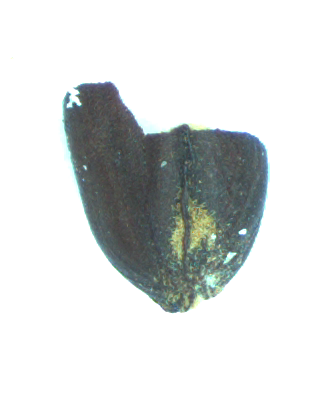

Supplement: S1 Data — (ZIP) [file pone.0273057.s001.zip › Supporting Information/Broken cotton seed/Image_19.bmp]

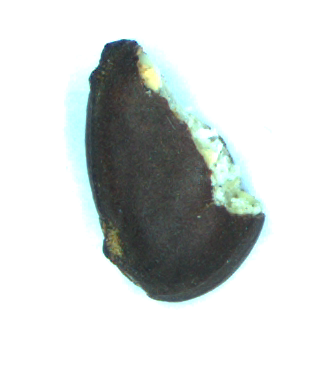

Supplement: S1 Data — (ZIP) [file pone.0273057.s001.zip › Supporting Information/Broken cotton seed/Image_2.bmp]

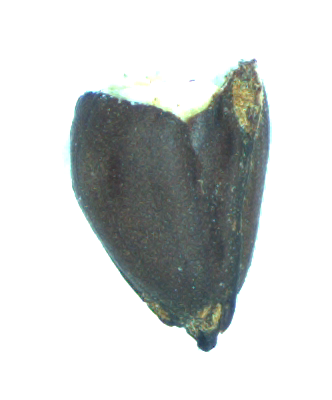

Supplement: S1 Data — (ZIP) [file pone.0273057.s001.zip › Supporting Information/Broken cotton seed/Image_20.bmp]

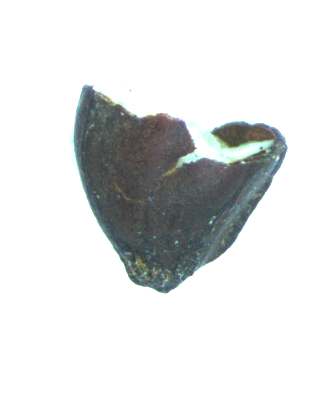

Supplement: S1 Data — (ZIP) [file pone.0273057.s001.zip › Supporting Information/Broken cotton seed/Image_21.bmp]

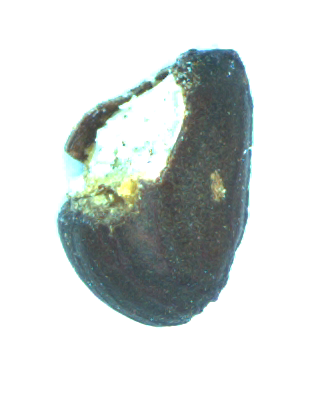

Supplement: S1 Data — (ZIP) [file pone.0273057.s001.zip › Supporting Information/Broken cotton seed/Image_22.bmp]

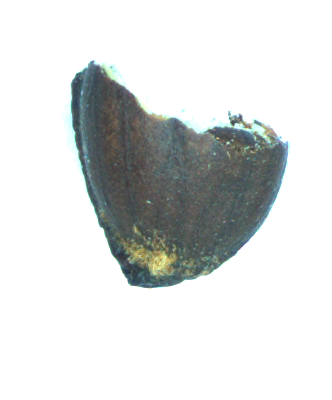

Supplement: S1 Data — (ZIP) [file pone.0273057.s001.zip › Supporting Information/Broken cotton seed/Image_23.bmp]

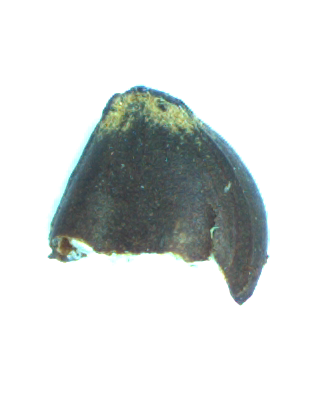

Supplement: S1 Data — (ZIP) [file pone.0273057.s001.zip › Supporting Information/Broken cotton seed/Image_24.bmp]

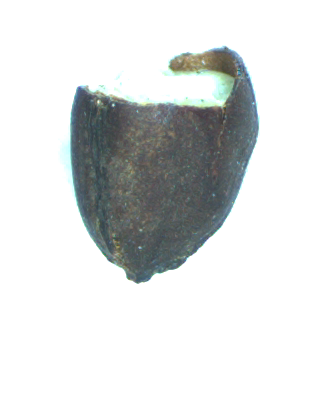

Supplement: S1 Data — (ZIP) [file pone.0273057.s001.zip › Supporting Information/Broken cotton seed/Image_25.bmp]

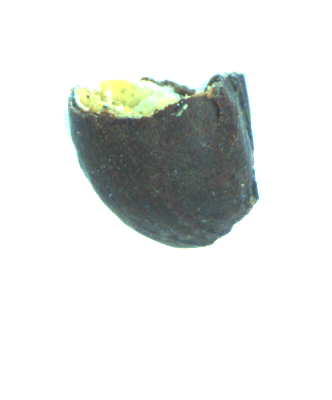

Supplement: S1 Data — (ZIP) [file pone.0273057.s001.zip › Supporting Information/Broken cotton seed/Image_26.bmp]

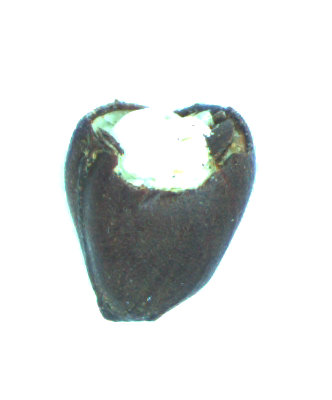

Supplement: S1 Data — (ZIP) [file pone.0273057.s001.zip › Supporting Information/Broken cotton seed/Image_27.bmp]

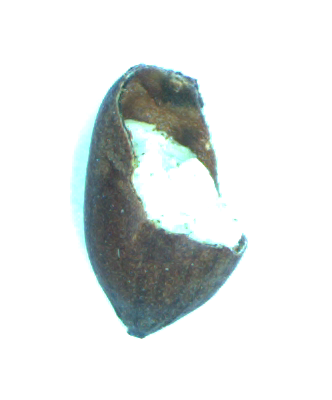

Supplement: S1 Data — (ZIP) [file pone.0273057.s001.zip › Supporting Information/Broken cotton seed/Image_28.bmp]

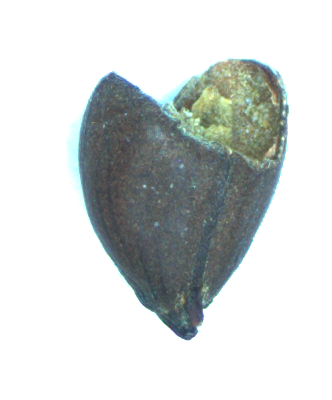

Supplement: S1 Data — (ZIP) [file pone.0273057.s001.zip › Supporting Information/Broken cotton seed/Image_29.bmp]

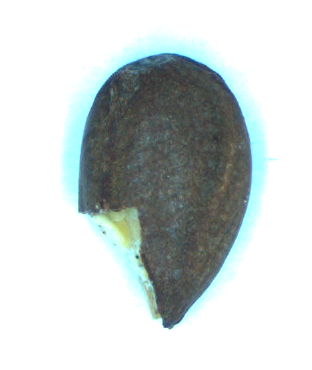

Supplement: S1 Data — (ZIP) [file pone.0273057.s001.zip › Supporting Information/Broken cotton seed/Image_3.bmp]

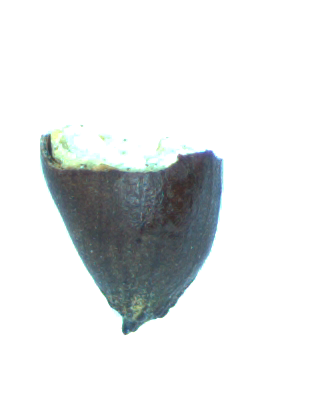

Supplement: S1 Data — (ZIP) [file pone.0273057.s001.zip › Supporting Information/Broken cotton seed/Image_30.bmp]

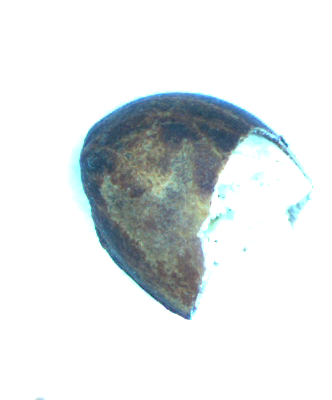

Supplement: S1 Data — (ZIP) [file pone.0273057.s001.zip › Supporting Information/Broken cotton seed/Image_31.bmp]

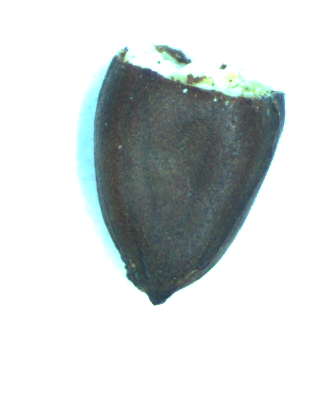

Supplement: S1 Data — (ZIP) [file pone.0273057.s001.zip › Supporting Information/Broken cotton seed/Image_32.bmp]

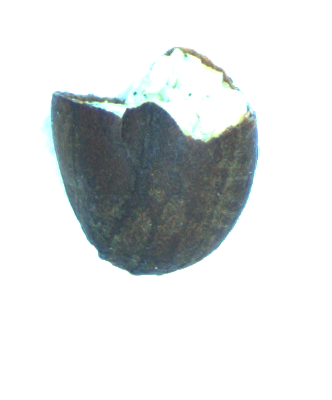

Supplement: S1 Data — (ZIP) [file pone.0273057.s001.zip › Supporting Information/Broken cotton seed/Image_33.bmp]

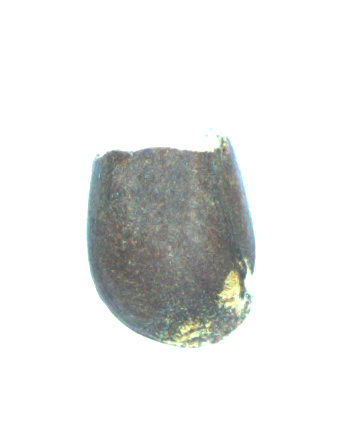

Supplement: S1 Data — (ZIP) [file pone.0273057.s001.zip › Supporting Information/Broken cotton seed/Image_34.bmp]

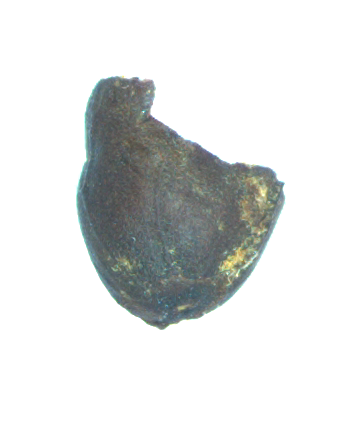

Supplement: S1 Data — (ZIP) [file pone.0273057.s001.zip › Supporting Information/Broken cotton seed/Image_35.bmp]

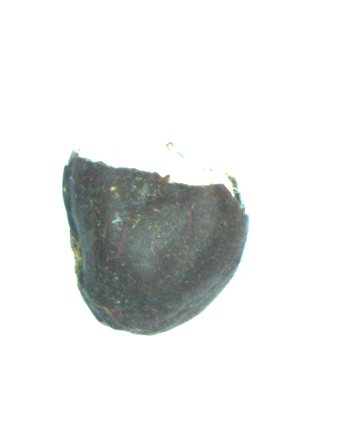

Supplement: S1 Data — (ZIP) [file pone.0273057.s001.zip › Supporting Information/Broken cotton seed/Image_36.bmp]

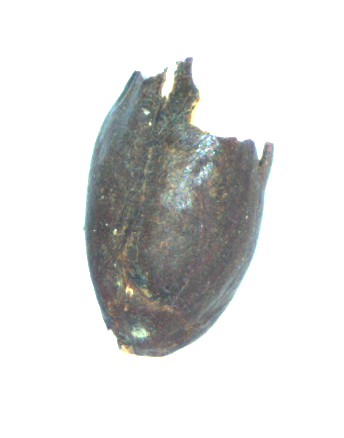

Supplement: S1 Data — (ZIP) [file pone.0273057.s001.zip › Supporting Information/Broken cotton seed/Image_37.bmp]

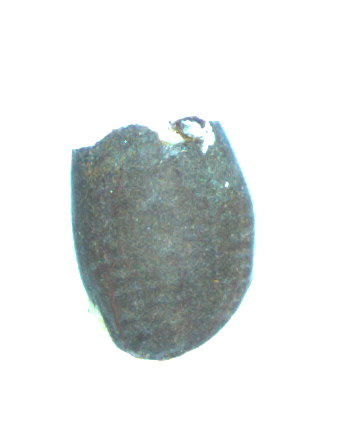

Supplement: S1 Data — (ZIP) [file pone.0273057.s001.zip › Supporting Information/Broken cotton seed/Image_38.bmp]

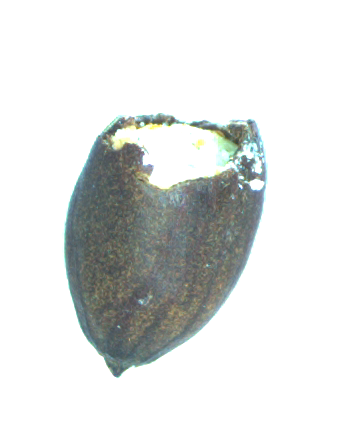

Supplement: S1 Data — (ZIP) [file pone.0273057.s001.zip › Supporting Information/Broken cotton seed/Image_39.bmp]

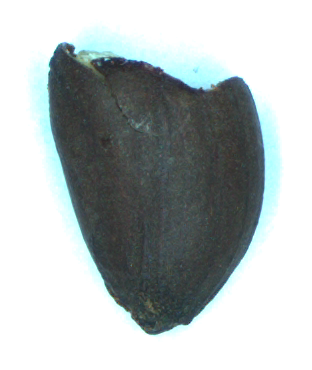

Supplement: S1 Data — (ZIP) [file pone.0273057.s001.zip › Supporting Information/Broken cotton seed/Image_4.bmp]

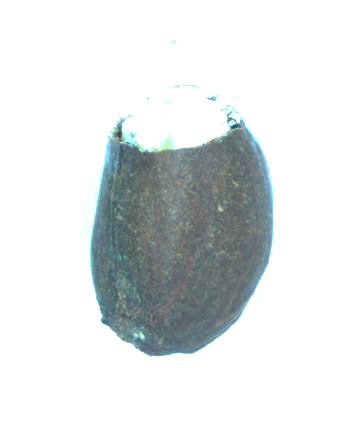

Supplement: S1 Data — (ZIP) [file pone.0273057.s001.zip › Supporting Information/Broken cotton seed/Image_40.bmp]

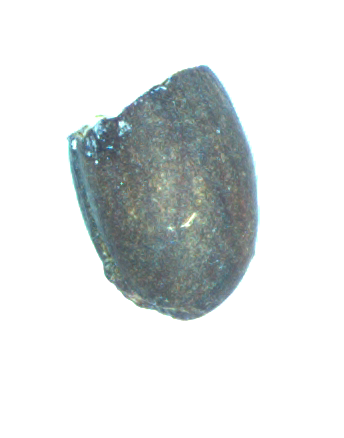

Supplement: S1 Data — (ZIP) [file pone.0273057.s001.zip › Supporting Information/Broken cotton seed/Image_41.bmp]

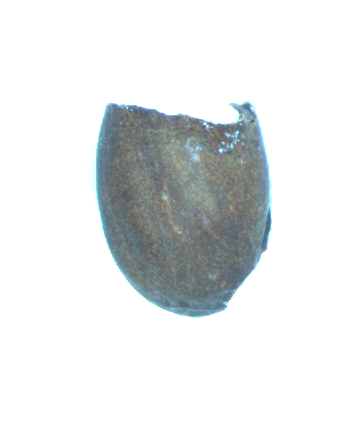

Supplement: S1 Data — (ZIP) [file pone.0273057.s001.zip › Supporting Information/Broken cotton seed/Image_42.bmp]

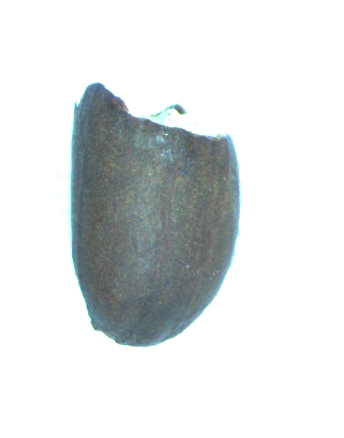

Supplement: S1 Data — (ZIP) [file pone.0273057.s001.zip › Supporting Information/Broken cotton seed/Image_43.bmp]

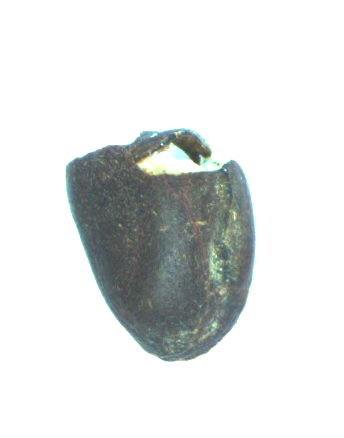

Supplement: S1 Data — (ZIP) [file pone.0273057.s001.zip › Supporting Information/Broken cotton seed/Image_44.bmp]

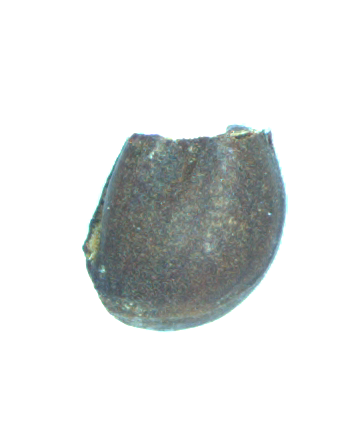

Supplement: S1 Data — (ZIP) [file pone.0273057.s001.zip › Supporting Information/Broken cotton seed/Image_45.bmp]

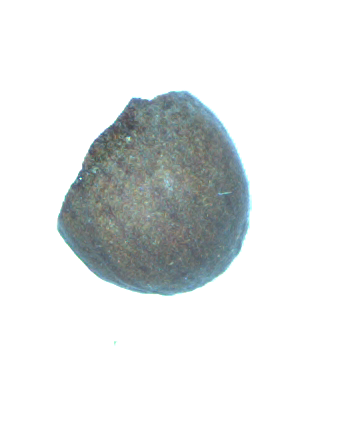

Supplement: S1 Data — (ZIP) [file pone.0273057.s001.zip › Supporting Information/Broken cotton seed/Image_46.bmp]

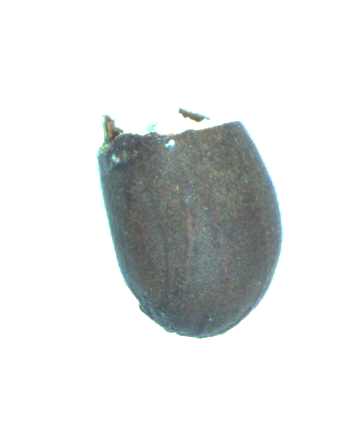

Supplement: S1 Data — (ZIP) [file pone.0273057.s001.zip › Supporting Information/Broken cotton seed/Image_47.bmp]

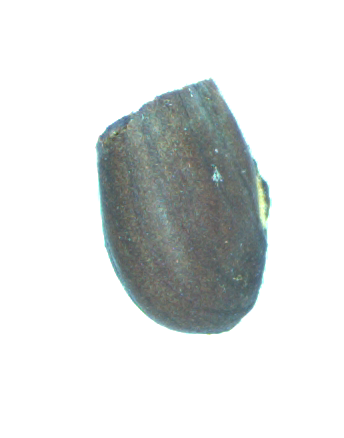

Supplement: S1 Data — (ZIP) [file pone.0273057.s001.zip › Supporting Information/Broken cotton seed/Image_48.bmp]

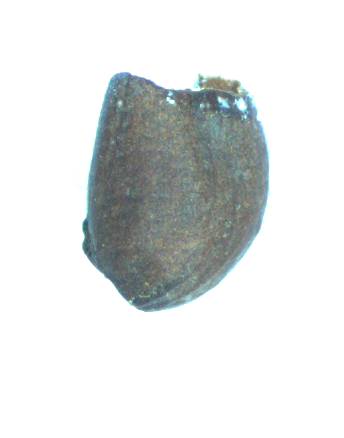

Supplement: S1 Data — (ZIP) [file pone.0273057.s001.zip › Supporting Information/Broken cotton seed/Image_49.bmp]

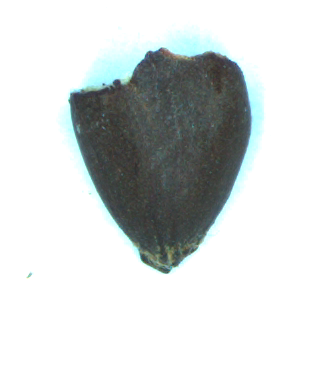

Supplement: S1 Data — (ZIP) [file pone.0273057.s001.zip › Supporting Information/Broken cotton seed/Image_5.bmp]

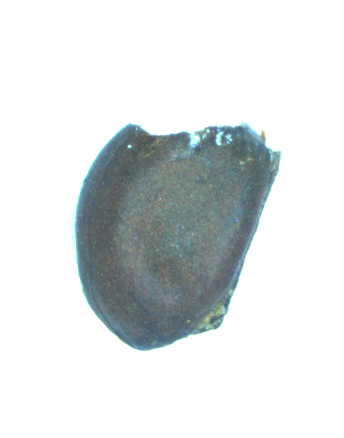

Supplement: S1 Data — (ZIP) [file pone.0273057.s001.zip › Supporting Information/Broken cotton seed/Image_50.bmp]

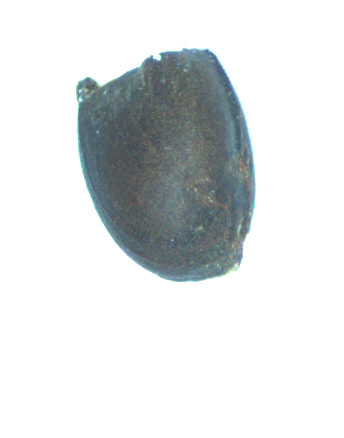

Supplement: S1 Data — (ZIP) [file pone.0273057.s001.zip › Supporting Information/Broken cotton seed/Image_51.bmp]

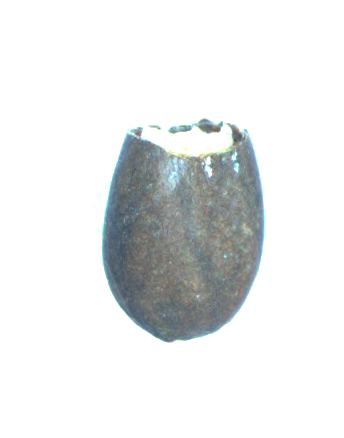

Supplement: S1 Data — (ZIP) [file pone.0273057.s001.zip › Supporting Information/Broken cotton seed/Image_52.bmp]

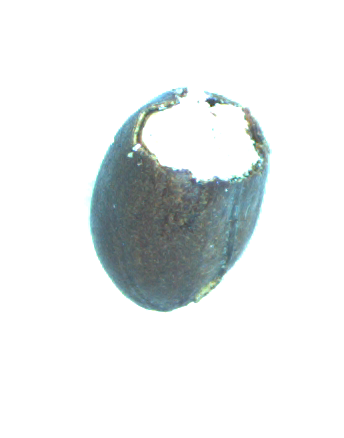

Supplement: S1 Data — (ZIP) [file pone.0273057.s001.zip › Supporting Information/Broken cotton seed/Image_53.bmp]
