# Supplementary material for: A method for detecting the quality of cotton seeds based on an improved ResNet50 model
Source: PLoS One. 2023 Feb 15;18(2):e0273057. doi: 10.1371/journal.pone.0273057 (PMC9931132; doi:10.1371/journal.pone.0273057)

PLOS ONE

Cotton seed quality identification method based on improved ResNet50 model

--Manuscript Draft--

**Manuscript Number:**	PONE-D-22-02418
**Article Type:**	Research Article
**Full Title:**	Cotton seed quality identification method based on improved ResNet50 model
**Short Title:**	Cotton seed quality identification
**Corresponding Author:**	Xinwu Du<br>Henan University of Science and Technology<br>Luoyang, CHINA
**Keywords:**	cotton seed; CBAM attention mechanism; transfer learning; improved ResNet50 model; visualization
**Abstract:**	This paper proposes a cotton seed quality detection method based on the improved ResNet50 model with automatic cotton seed quality detection as the research objective. The research takes intact cotton seed, broken cotton seed, and cracked cotton seed as the objects, and builds a cotton seed image database by making a cotton seed image acquisition system; then performs geometric transformations such as flip, rotation, random crop, and chromaticity change on cotton seed images to increase the diversity of data samples to improve the robustness of the model. The improved ResNet50 model is constructed by introducing the CBAM attention mechanism, redesigning the fully connected layer and the classification output layer, introducing the transfer learning mechanism to train the model, improving the LRelu-Softplus activation function to improve the model expressiveness, updating the gradient by the Adam optimization algorithm, and embedding the cosine annealing method to decay the learning rate. For validating the performance of the improved ReNet50 model, species identification was performed for three types of cotton species. The results show that the enhanced ResNet50 model can achieve an average recognition accuracy of 97.23%,approximately 5.05%, 3.16%, 2.39%,1.69%, and 1.02% better than several classical models of AlexNet, VGG16, GoogleNet, ResNet18, and ResNet50, respectively. Visualization results against the confusion matrix show that the improved ResNet50 model has better recognition performance and robustness, serving as a technical reference for recognizing cotton seeds.
**Order of Authors:**	Xinwu Du
	Laiqiang Si
	Pengfei Li
	Zhihao Yun
**Additional Information:**	
**Question**	**Response**
**Financial Disclosure**<br><br>Enter a financial disclosure statement that describes the sources of funding for the work included in this submission. Review the submission guidelines for detailed requirements. View published research articles from *PLOS ONE* for specific examples.<br><br>This statement is required for submission and **will appear in the published article** if the submission is accepted. Please make	Funding:National Natural Science Foundation of China (52075150), Natural Science Foundation of Henan Province (No.202300410124) and Guangdong Key R&D Program(No.2019B020222004)<br><br>Conceptualization: Xinwu Du.<br>Data curation: Zhihao Yun, Pengfei Li.<br>Funding acquisition: Xinwu Du.<br>Investigation: Laiqiang Si.<br>Methodology: Xinwu Du, Laiqiang Si, Zhihao Yun, Pengfei Li.<br>Project administration: Xinwu Du, Laiqiang Si, Zhihao Yun, Pengfei Li.<br>Resources: Xinwu Du, Laiqiang Si.<br>Software: Xinwu Du, Laiqiang Si.<br>Supervision: Xinwu Du.

Powered by Editorial Manager® and ProduXion Manager® from Aries Systems Corporation

sure it is accurate.<br><br>**Unfunded studies**<br>Enter: *The author(s) received no specific funding for this work.*<br><br>**Funded studies**<br>Enter a statement with the following details:<br>• Initials of the authors who received each award<br>• Grant numbers awarded to each author<br>• The full name of each funder<br>• URL of each funder website<br>• Did the sponsors or funders play any role in the study design, data collection and analysis, decision to publish, or preparation of the manuscript?<br>• **NO** - Include this sentence at the end of your statement: *The funders had no role in study design, data collection and analysis, decision to publish, or preparation of the manuscript.*<br>• **YES** - Specify the role(s) played.<br><br>* typeset	Validation: Xinwu Du.<br>Visualization: Xinwu Du.
**Competing Interests**<br><br>Use the instructions below to enter a competing interest statement for this submission. On behalf of all authors, disclose any competing interests that could be perceived to bias this work—acknowledging all financial support and any other relevant financial or non-financial competing interests.<br><br>This statement is required for submission and **will appear in the published article** if the submission is accepted. Please make sure it is accurate and that any funding sources listed in your Funding Information later in the submission form are also declared in your Financial Disclosure statement.<br><br>View published research articles from *PLOS ONE* for specific examples.	The authors have declared that no competing interests exist.

Powered by Editorial Manager® and ProduXion Manager® from Aries Systems Corporation

**NO authors have competing interests**<br><br>Enter: *The authors have declared that no competing interests exist.*<br><br>**Authors with competing interests**<br><br>Enter competing interest details beginning with this statement:<br><br>*I have read the journal's policy and the authors of this manuscript have the following competing interests: [insert competing interests here]*<br><br>* typeset	
**Ethics Statement**<br><br>Enter an ethics statement for this submission. This statement is required if the study involved:<br><br>• Human participants<br>• Human specimens or tissue<br>• Vertebrate animals or cephalopods<br>• Vertebrate embryos or tissues<br>• Field research<br><br>Write "N/A" if the submission does not require an ethics statement.<br><br>General guidance is provided below. Consult the submission guidelines for detailed instructions. **Make sure that all information entered here is included in the Methods section of the manuscript.**	N/A

**Format for specific study types**<br><br>**Human Subject Research (involving human participants and/or tissue)**<br>• Give the name of the institutional review board or ethics committee that approved the study<br>• Include the approval number and/or a statement indicating approval of this research<br>• Indicate the form of consent obtained (written/oral) or the reason that consent was not obtained (e.g. the data were analyzed anonymously)<br><br>**Animal Research (involving vertebrate animals, embryos or tissues)**<br>• Provide the name of the Institutional Animal Care and Use Committee (IACUC) or other relevant ethics board that reviewed the study protocol, and indicate whether they approved this research or granted a formal waiver of ethical approval<br>• Include an approval number if one was obtained<br>• If the study involved *non-human primates*, add *additional details* about animal welfare and steps taken to ameliorate suffering<br>• If anesthesia, euthanasia, or any kind of animal sacrifice is part of the study, include briefly which substances and/or methods were applied<br><br>**Field Research**<br><br>Include the following details if this study involves the collection of plant, animal, or other materials from a natural setting:<br>• Field permit number<br>• Name of the institution or relevant body that granted permission	
**Data Availability**<br><br>Authors are required to make all data underlying the findings described fully available, without restriction, and from the time of publication. PLOS allows rare exceptions to address legal and ethical concerns. See the PLOS Data Policy and FAQ for detailed information.	Yes - all data are fully available without restriction

A Data Availability Statement describing where the data can be found is required at submission. Your answers to this question constitute the Data Availability Statement and **will be published in the article**, if accepted.<br><br>**Important:** Stating 'data available on request from the author' is not sufficient. If your data are only available upon request, select 'No' for the first question and explain your exceptional situation in the text box.<br><br>Do the authors confirm that all data underlying the findings described in their manuscript are fully available without restriction?	
**Describe where the data may be found in full sentences. If you are copying our sample text, replace any instances of XXX with the appropriate details.**<br><br>• If the data are **held or will be held in a public repository**, include URLs, accession numbers or DOIs. If this information will only be available after acceptance, indicate this by ticking the box below. For example: *All XXX files are available from the XXX database (accession number(s) XXX, XXX.).*<br>• If the data are all contained **within the manuscript and/or Supporting Information files**, enter the following: *All relevant data are within the manuscript and its Supporting Information files.*<br>• If neither of these applies but you are able to provide **details of access elsewhere**, with or without limitations, please do so. For example:<br><br>*Data cannot be shared publicly because of [XXX]. Data are available from the XXX Institutional Data Access / Ethics Committee (contact via XXX) for researchers who meet the criteria for access to confidential data.*<br><br>*The data underlying the results presented in the study are available from (include the name of the third party*	All relevant data are within the manuscript and its Supporting Information files.

and contact information or URL).<br>• This text is appropriate if the data are owned by a third party and authors do not have permission to share the data.<br><br>* typeset	
Additional data availability information:	

Manuscript Click here to access/download;Manuscript;Manuscript.docx

Cotton seed quality identification method based on improved ResNet50 model

Xinwu Du[1,2,*], Laiqiang Si[1], Pengfi Li[1], Zhihao Yun[1],

[1]College of Agricultural Equipment Engineering, Henan University of Science and Technology, Luoyang, Henan 471003, China
[2]Henan Collaborative Innovation Center for Advanced Manufacturing of Machinery and Equipment, Henan Luoyang 471003, China
[*]Correspondence author：du_xinwu@sina.com.cn

Abstract:

This paper proposes a cotton seed quality detection method based on the improved ResNet50 model with automatic cotton seed quality detection as the research objective. The research takes intact cotton seed, broken cotton seed, and cracked cotton seed as the objects, and builds a cotton seed image database by making a cotton seed image acquisition system; then performs geometric transformations such as flip, rotation, random crop, and chromaticity change on cotton seed images to increase the diversity of data samples to improve the robustness of the model. The improved ResNet50 model is constructed by introducing the CBAM attention mechanism, redesigning the fully connected layer and the classification output layer, introducing the transfer learning mechanism to train the model, improving the LRelu-Softplus activation function to improve the model expressiveness, updating the gradient by the Adam optimization algorithm, and embedding the cosine annealing method to decay the learning rate. For validating the performance of the improved ReNet50 model, species identification was performed for three types of cotton species. The results show that the enhanced ResNet50 model can achieve an average recognition accuracy of 97.23%,approximately 5.05%, 3.16%, 2.39%,1.69%, and 1.02% better than several classical models of AlexNet, VGG16, GoogleNet, ResNet18, and ResNet50, respectively. Visualization results against the confusion matrix show that the improved ResNet50 model has better recognition performance and robustness, serving as a technical reference for recognizing cotton seeds.

Keywords: cotton seed; CBAM attention mechanism; transfer learning; improved ResNet50 model; visualization

Introduction

Cotton seed is the most basic production material for cotton production, and the quality of cotton seed directly determines the yield and fiber quality of cotton. Data show that the contribution of good seed to agricultural yield increased by about 45% [1-3]. In recent years, cotton precision sowing technology has received full attention, which improves the quality of sowing, saves 50% to 70% of the sowing amount of cotton seed, and reduces the production cost. This technology requires the precise realization of agronomic specifications of one hole and one grain and therefore imposes strict requirements on the quality of cotton seed [4-6]. Cotton seed is processed through the ginning machine, stripping machine, centrifugal barrel finishing, polishing machine, and other processes to cause the cotton seed to break and produce surface cracks. Such seeds are not easy to store to affect the germination rate. Therefore, the quality of lint-free cotton seed needs to be tested before sowing.

In the production of cotton seeds, by introducing the principles of air screen, magnetic screen, socket screen, gravity screen, dielectric screen, and other tenets of mechanized sorting equipment to replace the manual screening, significantly improving the screening efficiency of cotton seeds in actual production, this method can screen grain whole cotton seeds in large quantities. It can effectively remove stones or deflection grains in raw materials, but the quality and appearance of similar immature cotton seed, broken and cracked cotton seed screening effect is poor[7]. Machine vision technology in the non-destructive testing application of research a lot, broad prospects, traditional machine vision technology through the linear discriminant model, artificial neural network, support vector machine and other algorithms can remove immature cotton seeds, broken cotton seeds. However, the surface crack cotton seed screening effect is still poor, and other algorithms need to extract the method for different cotton seed characteristics. The sorting module is expensive, real-time, and stable [8-11]. Therefore, practical sorting algorithms for damaging cotton seeds need to be studied.

In recent years, the deep learning technology represented by convolutional neural networks has been widely used in the field of image recognition; compared with the traditional image detection technology, convolutional neural networks have increased the deep network structure with feature learning, which can automatically extract

the most representative image features [12,13]. Researchers at home and abroad apply convolutional neural networks t[illegible]d quality detection of corn, wheat grains, peanuts and other crops [14-17]. Du et al. constructed a 6-layer C[illegible]to extract spectral characteristics of macadamia nuts, used the activation function Relu and Dropout method to avoid overfitting, and realized the classification of three different qualities of macadamia nuts [18]. Przybyło et al. based on the local and global characteristics of CNN learning acorns, which are used to identify health and deteriorated acorns, the model recognition accuracy is 85% compared to manual predictions [19]. Altuntaş et al. were inspired by deep transfer learning. They employed transfer learning convolutional neural networks to identify ha[illegible] and diploid maize seeds, and the optimal recognition accuracy of the model reached 94.22% [20]. Ni et a[illegible]posed a maize seed inspection system based on the ResNet deep convolutional neural network architecture. The model's prediction accuracy for different quality of maize seed reached 98.20% [21]. Zhang et al. proposed a method for identifying common defects such as peanut mildew, crushing and drying based on machine vision and adaptive convolutional neural networks, with the automatic detection of peanut quality as the research goal, and the average recognition rate of common defects of peanuts reached 99.70% [22]. Zhu et al. designed a complete wheat grain and broken wheat grain identification model based on a convolutional neural network based on convolutional neural network for rapid and accurate identification of wheat grain appearance quality, with an average recognition accuracy of 96. 67% [23]. Yang et al. suggested a grape seed image classification recognition algorithm based on Gabor wavelet feature extraction and multilayered neural network to change the traditional manual empirical judgment, which effectively identified immature grape seed [24]. Gulzar et al. submitted a transfer learning convolutional neural network for a seed classification system with 99% accuracy for 14 common seeds to increase seed classification efficiency [25]. Loddo et al. developed a new CNN model, SeedNet, to classify more than twenty different kinds of seed images with an average recognition accuracy of 97.47% [26]. Kurtulmuş et al. studied three deep learning models, AlexNet, GoogleNet and ResNet, to recognise sunflower seed, where the GoogleNet algorithm achieved the highest classification accuracy of 95% [27]. Zhao et al. designed and developed a sorting system based on deep learning to identify the broad surface of soybean for accurate sorting of high-quality soybean seed and tested the classification accuracy of the improved MobileNetV2 based model for soybean defects 97.84% [28]. Vidyarthi et al. for detecting the quality of the amygdala, based on image processing and convolutional neural network for rapid and automatic classification of the amygdala, the highest value of classification accuracy for five types of adulterated amygdala reached 99% with excellent recognition results [29]. Koklu et al. compared the classification performance of artificial neural networks, multilayer perceptrons, and convolutional neural networks to distinguish rice varieties. The results showed a high discrimination accuracy for the classification of five rice varieties based on the improved VGG16 network model [30]. Previous studies have shown that convolutional neural networks have beneficial consequences for seeds with different visual appearances, such as different species torn or defective seeds. It is feasible to apply CNN to target recognition and quality detection of cotton seeds.

In this paper, three types of cotton seed, intact cotton seed, broken cotton seed and cracked cotton seed, were chosen to propose a cotton seed quality identification method to improve the ReNet50 model—addition of the fully connected layer Fc1 to the ResNet50 model and the addition of Dropout. The model training introduced the transfer learning model training mechanism, improved the LRelu-Softplus activation function, updated the gradient by Adam optimization algorithm, and embedded the cosine annealing method to attenuate the learning rate to achieve the accurate classification and recognition of cotton seed quality in the case of small cotton seed image samples.

Experimental materials and pre-processing

Image acquisition

Using GK-10 lazy cotton seed deflected photon harvested in 2021 as the test material, the cotton seed of this variety is primarily planted, high yield, insect and disease resistant, adaptable, and has certain representativeness. A random sample of 50 purchased materials, each with 100 selected cotton seeds for integrity testing. The results showed that the proportion of cracked and partially broken cotton seeds in the materials ranged from 5% to 10%. The intact cotton seeds, broken cotton seeds, and cracked cotton seeds in the material were observed, among which the intact cotton seeds were brown, the edges were entire, and there were no obvious defects on the surface; the boundaries of the broken cotton seeds were broken to expose the milky white endosperm, and there were different types of cracks on the surface of the cracked cotton seeds. The color scale changes occurred at the breaks.

In the indoor e[illegible]onment (Natural light + energy-saving lamp), cotton samples were taken vertically from 15 cm height by a C[illegible] camera. Each image contained about 18 cottonseeds with a 4024 × 3036 dpi resolution. A total of 3154 valid photos were collected, as shown in Fig 1.

(a) (b)

Fig 1. Cotton seed acquisition. (a) Image acquisition system. 1. Camera 2. Lens 3.L[illegible]4. Cotton seed 5. Platform 6. Image monitor;(b) Cotton seed image

The original images acquired cannot be used directly for network training, cropped these images into a sample of 224pixel × 224pixel that fully contain cotton seed, and Fig 2. is a single cotton seed image the corresponding

background image of the removal. A database of cotton seed images was established, with 4417 pieces, including 1367 intact cotton seeds, 1584 broken seeds, and 1 466 cracked cotton seeds.

Fig 2. Single cotton image

Data augmentation

For enhanced generalisation and robustness of the model, the data was expanded by flipping, rotating, scaling, cropping, panning and adding noise to the three types of cotton images. The amplified cotton seed image dataset consisted of 7386 images, which were divided using a python program, randomly dividing the dataset into 80% training sets and 20% validation sets, of which 5910 images were used as the training set to train the model, 1476 images are used as a validation set to tune the model parameters and validate the model, and 450 unlabeled images are used as a test set to evaluate the model's classification performance on unknown data sets. An example of dataset partitioning is given in table 1.

Table 1. Number of image data of different cotton varieties

Category	Dataset	Training set	Validation set
Instact seed	2367	1894	473
Broken seed	2465	1972	493
Cracked seed	2554	2044	510

ResNet50 model

With the deepening of the depth of convolutional neural networks, gradient degradation and disappearance will occur during the network training, resulting in difficulty in training models and low accuracy. Increasing the residual structure in convolutional neural networks can avoid this phenomenon to a large extent. Fig 3 displays the ResNet50 model and the residual design [31].

The input feature image becomes half of the original height and width through the residual structure 3a, while in the residual structure 3b, the height and width of the feature map remain unchanged. Fig 3 illustrates the Stage4 residual structure block as an example. The residual structure blocks allow for a reduction in training parameters and more effective and intuitive feature extraction, avoiding the problem of gradient dispersion and enhancing the feature learning capability of the model.

Fig 3c shows the classical ResNet50 model structure, with 50 layers, including 49 convolutional layers and one fully-connected layer. The model first passes through the stem layer, entering a convolutional layer of 7×7×64, followed by 16 residual structural blocks, each block consisting of 3 convolutional layers for a total of 48 layers, and finally through a fully connected layer.

Fig 3. The network structure of Resnet50 model

CBAM attention mechanism

CBAM is a lightweight, versatile convolutional attention module that combines channel attention and spatial attention, and the introduction of CBAM in the ResNet50 network can significantly improve classification accuracy. However, adding CBAM at each stage increases the number of parameters. It cannot highlight the key characteristics of the target object, which will mislead the next step of feature extraction. Therefore, only high-level features extracted by Stage4 of the ResNet50 network are introduced into CBAM [32,33]. The CBAM model is presented in Fig 4.

Fig 4. CBAM module

The CBAM module first enters the feature map F, and the result is F' after the channel attention weighting. After spatial attention weighting, the output feature diagram F'' is obtained, which is a "cascade connection" structure with the process formula:

$$\begin{cases} F' = M_C \otimes F \\ F'' = M_S \otimes F' \end{cases} \quad (0)$$

Where M_c is the output weights after channel attention.M_S is the output weight after spatial attention. ⊗ is the sign of the feature map weighted multiplication operation.

Improved ResNet50 network structure optimization

The cotton seed identification model of the improved residual network proposed in this paper retains the feature extraction layer based on the original ResNet50 network structure and introduces the CBAM attention

mechanism after the Stage4 residual module, redesigning the fully-connection layer and the classified output layer of the network model, and the design of the improved ResNet50 model is shown in Fig 5 [34].

Fig 5. Improved ResNet50 model

The transfer is learning training mechanism based on middle layer expression is adopted, which significantly accelerates the convergence speed of model training and obtains higher recognition accuracy on limited cotton seed samples. According to the improved ResNet50 model structure, its fully connected layer, loss function, optimizer, and learning rate attenuation need to be improved and optimized.

Activation function

The Relu activation function is widely used in convolutional neural networks because of its fast operation speed and good performance, but the Relu activation parameter cannot continue to be updated when the input is less than 0; the Leaky-Relu activation function is linear, and only a threshold is needed to get the activation value, no complex operation is required, and when the input is less than 0, the parameters can still be updated. However, in the training of the model, it is necessary to adjust the parameters to find a suitable slowly declining parameter; the Softplus activation function circumvents the disadvantage of the forced sparseness of the return activation function, but the function fails to solve the function offset phenomenon like Relu, which affects the convergence performance of the model; considering the characteristics of the three functions, combined with the LRelu and Softplus functions, an improved activation function is designed, that is, the LRelu-Softplus activation function. LReLu-Softplus function, the formula is:

$$f(x)=\begin{cases}\alpha x & (x\leqslant 0)\\ \ln(e^x+1)-\ln 2 & (x>0)\end{cases} \tag{2}$$

Where α is a constant, close to 0. In this paper, the initial value is placed at 0.15, and Fig 6 shows the schematic diagram of the function.

Add full connection layer and Dropout

A fully connected layer is in addition to the ResNet50 model structure, enabling the model to extract more feature map information while adding a Dropout mechanism to prevent overfitting. In the Dropout process, neurons inactivate some neurons with a certain probability, reduce the dependence between neurons, improve the

Fig 6. LRelu-Softplus activation function **Fig 7.** The Full connection layer and Dropout

generalization ability of the model. This paper sets the Dropout to take 0.5, in the model training randomly inactivated half of the neurons, forward propagation when inactivated disconnected neurons no longer participate in the update of model parameters. The fully connected layer and Dropout are shown in Fig 7.

Loss function

Since this paper is based on an improved ResNet50 model to identify three types of cotton seeds, intact, broken and cracked cotton seeds, Multi-category cross-entropy is used as a loss function, the expression of which is:

$$Loss=-\frac{1}{M}\sum_{m}^{i}\sum_{k}^{j}t_{ij}\lg y_{ij} \tag{3}$$

Where Loss is the loss function; M is the number of samples and k is the number of classes; t_{ij} is the probability distribution value of the jth category of the ith sample in the true value of the label; and y_{ij} represents the jth category of the ith sample in the predicted value of the network Probability distribution value.

Optimizer and learning rate decay

Network model parameters are numerous, the need for a suitable optimization algorithm to learn parameters, Adam is the most widely used optimizer, in the loss gradient update, can set the adaptive learning rate for the neural network, this paper in the training of improved ResNet50 network model to choose Adam optimization algorithm, the default learning rate is 0.001, exponential decay rate β1, β2 are 0.9, 0.999, divide-by-zero error terms 1×10^{-8}. Learning rate attenuation can significantly improve the performance of Adam's optimization algorithm. So this paper uses the cosine annealing attenuation learning rate provided by the PyTorch framework to speed up model convergence when improving ResNet50 model training.

Model training

Training Platform

The hardware environment of this experiment is memory: 16 G, CPU: Intel (R) Xeon (R) E5, GPU: NVIDIA GTX 1060 6G, the operating system is Windows 10, the selected deep learning open-source architecture is PyTorch, and the parallel operation of convolutional neural networks is realized by PyTorch calling the GPU.

The training process of the model

Under the supervised learning mode, a large amount of data is required to train the model. Still, at present, the labelled cotton seed image data is small, which is challenging to meet the needs of training deep network models, so the introduction of transfer learning mechanism training mechanism, the pre-trained weight file on the ImageNet dataset is loaded into the model of this paper, which can reduce not only the amount of image data required but also obtain faster training speed and better convergence performance [35-37]. The training process for the model is given in Fig 8.

Fig 8. Procedure for training

The specific training process is as follows:

(1) Through the data set loading method in the Pytorch deep learning framework, load the ResNet50 pre-trained model, and then train the Impro-ResNet50 network, at first only allow the training of the parameters of the last fully connected layer random initialization, and then optimize the parameters of the Impro-ResNet50 network using a small learning rate after the network converges. For each training, the batch size is 16, and the learning rate of each iteration is adjusted by the Adam optimization algorithm and cosine annealing to decrease gradually with the number of iterations. The parameters are updated once per batch of samples as the gradient descends, with the number of iterations configured in 300 and the initial learning rate set to 0.001. The model training parameter values are given in Table 2.

Table 2. Model training parameter values

Parameter	Value
epoch	300
batch size	16
learning rate	0.001
Exponential decay rates β_1, β_2	0.9、0.999
Zeroing error item ε	1×10^{-8}

(2) Model training on the training set and test results on the validation set. Model training uses the "early stop" technique to prevent overfitting. As the number of iteration rounds increases, if the validation error is found to rise, the training is stopped, and the weights after the stop are taken as the final parameters of the model;

(3) The neural network obtains the predicted value through forwarding propagation, calculates the error between the expected value and the actual value, and terminates the training iteration if the error meets the threshold set by the system; Otherwise, continue training until the upper limit of the number of training rounds set by the system is reached, and stop training;

(4) In each round of training, store the best training model and verify the model performance on the test set.

Improved ResNet50 model hyperparameter optimization

Comparative analysis of different learning rates

Learning rate as an essential hyperparameter in supervised learning has a more significant impact on the model. In the Adam optimization algorithm, when the learning rate is set too small, the model convergence process becomes very slow; setting too large can easily lead to gradient oscillation or even unable to converge. Selecting the appropriate learning rate allows the model's objective function to connect to a local minimum in a short period. In this way, the training accuracy and test accuracy rate are obtained under the same number of iterations. Select the initial batch size setting to 16, and because the default value for the learning rate in the Adam optimization algorithm is 0.001, take the parameter values of different orders of magnitude and compare the training effect of the model at a learning rate of 0.1, 0.01, 0.001, 0.0001, 0.00001. The results are presented in Fig 9.

Fig 9. Effects of learning rate on training accuracy and training loss

As can be observed in Fig.9, the learning rate is 0. 1. When the model converges slowly and does not converge at 0.00001, and the loss value is more significant; the learning rate is 0.01, 0.001, 0.0001, the model achieves a good convergence effect, of which when the learning rate is 0.0001, the model convergence speed is the fastest, and the loss value after convergence is the lowest. The test accuracy is highest when the network has a learning rate of 0.0001 after 300 rounds of training, so the learning rate is 0.0001.

The effect of the activation function on model performance

Activation function plays a vital role in the training of convolutional neural networks. For the nonlinear modelling ability of the network, there is no activation function, convolutional neural network. Even if there are more hidden layers, the network can only express linear mapping, and only after adding activation functions, deep neural networks have hierarchical nonlinear mapping learning capabilities. The learning rate is determined above to be 0.000 1. The learning rate is fixed, the model is trained with different activation functions, and the training loss value and accuracy rate change are shown in Fig 10.

As shown in Figure 10, after using the four activation functions of Relu, LRelu, Softplus and LRelu-Softplus, the model training loss value continues to decrease as the iteration round increases. The impact of the convergence speed of the model using the LRelu-Softplus activation function is greater than that of the other three functions. The model has begun to converge around the first 50 rounds and gradually approaches zero. Therefore, the LRelu-Softplus function is used as the model activation function in the improved ResNet50 model training.

Fig 10. Effects of activation function on training accuracy and training loss

Effect of the fully connected layer on model performance

The fully connected layer integrates all the local features extracted by the convolution layer into a global part to remove the spatial position information through the weight matrix. The fully connected layer contains the most parameters, which is an essential factor affecting the model's size. In order to determine the optimal number of fully connected layers in the model, the fully connected layer is increased from 1 to 3 in turn, and the results are shown in Fig 11.

As shown from Fig 11, when there are one layer and two fully connected layers, the model performance is improved than when there are three layers of fully connected layers. The three fully connected layers lead to a decrease in the convergence speed and accuracy of the model, which may be the increase of the fully connected layer leading to excessive parameter models overfitting, comparing when there is one layer. Two layers of fully connected layers, the two layers of fully connected layers make the convergence of the model more rapid and smooth. The loss value is lower when reaching stability, so the model is designed as two fully connected layers.

Fig 11. Effects of fully connected layers on training accuracy and training loss

Comparison of different neural network models

In this paper, the model performance characterization parameters (training accuracy, training time) are taken as the target parameters, and the Impro-ResNet50 model is compared with AlexNet, VGG16, GoogleNet, Res-Net18, and ResNet50, and Fig 12 and Fig 13 show the training curves, recognition accuracy and training time of several models. From Fig.12, we can see that the accuracy rate and loss value degradation process of the Impro-ResNet50 on the validation set is very smooth, the training process did not appear an extensive range of oscillation phenomenon, the model convergence rapidly began to converge at about 50 rounds, and the model loss value was close to 0 after 300 rounds of training. From Fig.13, it can be seen that the Impro-ResNet50 model in this paper has the highest recognition accuracy on the cotton seed validation set, which is 1.02~5.05% higher than that of several other network models, from the training time point of view, AlexNet model training time is the shortest, and the Impro-ResNet50 model training time in several networks is longer. However, the Impro-ResNet50 model is suitable for careful consideration of cotton seeds classification tasks within sufficient training time.

Fig 12. Performance comparison between improved-ResNet50 model and different classical models

Fig 13. Comparison of Type Identification Results of Several Models

Visual analysis of the identification results of cotton seeds by confusion matrix

It is not accurate to measure the model's performance only by training and testing accuracy in classification problems. When the sample data is small, or the distribution is unbalanced, it is easy to misclassify a few samples. Confusion matrices have good visibility when judging the merits and performance of classification models. The indicators that measure model performance in confusion matrices are usually Accuracy, Precision, Recall, and F1-score, with the following formula [38-40]:

$$Accuracy = \frac{TP + TN}{TP + TN + FP + FN} \quad (4)$$

$$Precision = \frac{TP}{TP + FP} \quad (5)$$

$$Recall = \frac{TP}{TP + FN} \tag{6}$$

$$F1\text{-}score = 2 \times \frac{Precision \times Recall}{Precision + Recall} \tag{7}$$

Where, TP = True Positive, TN = True Negative, FP = False Positive, FN= False Negative. Accuracy is the proportion of samples that predict correctly in all samples; Precision is the proportion of all prediction samples that the model predicts correctly; Recall indicates the percentage of correct model predictions in all true categories; F1-score is the average of the summation of precision and recall, which is a good balance between the two.

After 300 rounds of training, the Impro-ResNet50 model was tested, and 450 pictures were used as test data to count the Accuracy, Precision, Recall and F1-score of the model, and the results were shown in Table 3.

Table 3: Model training parameter values

Cotton seed classes		Predicate classes			Classification performance		
		Broken seed	Intact seed	Cracked seed	Precision/%	Recall/%	F1-score/%
True classes	Broken seed	146	0	4	97.33	99.31	98.32
	Intact seed	0	147	3	98.00	96.71	97.35
	Cracked seed	1	5	145	96.66	95.39	96.02

As can be seen from Table 3, the average classification accuracy of the model is 97.23%, and the classification performance (according to F1-score) depends on intact, broken, and cracked cotton seeds from highest to lowest. Amongst the 150 intact cotton seeds, 147 were correctly identified with an F1-score of 98. 00%; the 150 broken cotton seeds 146 were correctly identified with an F1-score of 97.98%. The model has a good recognition performance and robustness, as shown by 145 out of 150 broken cotton seeds were correctly identified with an F1-score of 96.02%. The model still had specific classification errors, where cracked cotton seeds had obvious classification errors and were incorrectly classified as intact cotton seeds. The main reason for this may be the dark color and low resolution of the collected cracked cottonseed images, among other reasons, making it difficult for the network to recognize the cracked features.

Conclusion

This study proposes a cotton seed identification method based on the Impro-ResNet50 model, adding the fully connected layer Fc1 and Dropout based on the classical ResNet50, introducing transfer learning for model training, using the improved LRelu-Softplus as the activation function, and using Adam optimization algorithm updates the gradient and embeds the attenuation learning rate of the cosine annealing method to identify three types of cotton seeds: intact cotton seeds, broken cotton seeds, and cracked cotton seeds. The results are as follows:

(1) In other cases where the conditions are the same when the learning rate is 0.0001 when the activation function is LRelu-Softplus and the two-layer fully connected layer is set, the model converges the fastest, the stability is the best, and the accuracy of the m[illegible] at the end of training is the highest;

(2) Compared with the performance of [illegible]Net, VGG16, GoogleNet, ResNet18, ResNet50, and the improved ResNet50 model in this paper, the enhanced model has a better recognition effect for three types of cotton seeds, with a test accuracy of 97.23%, which is 1.02~5.05% higher than the accuracy of several other network models;

(3) Through the visual analysis of the type identification results by the confusion matrix, the improved ResNet50 model has good recognition performance and robustness, and the Accuracy, Precision, Recall, and F1-score of each type of cotton seed can reach more than 95% in the test dataset, which further verifies the superiority of improving the performance of the improved ResNet50 model and can be applied to the cotton seed classification task;

(4) The model still has a specific rate of misclassification to provide model performance further: first, by expanding the dataset and increasing the sample categories to enhance the generalization ability of the model; second, the collection of images in the actual cotton seed production, which miscellaneous objects may obscure, poor light sources, etc., to increase the cotton seed images in complex backgrounds to lay the foundation for the industrial deployment of the model.

References

1. Peng JN, Xie ZM, Yang LM, et al. Quickly selection for cotton seed based on seed identification software. Transactions of the Chinese Society of Agricultural Engineering. 2013; 29(23): 147-152.

2. Soares SFC, Medeiros EP, Pasquini C, et al. Classification of individual cotton seeds with respect to variety using near-infrared hyperspectral imaging. Analytical Methods, 2016; 8(48): 8498-8505.
3. Gao P, Zhang C, Lü X, et al. Visual Identification of Slight-Damaged Cotton Seeds Based on Near Infrared Hyperspectral Imaging. Spectroscopy and Spectral Analysis. 2018; 38(6): 1712.
4. Chen XG, Zhao Y. Development of double-film mulch precision planter for cotton seeding. Transactions of the Chinese Society of Agricultural Engineering. 2010; 26(4): 106-112.
5. Zhang JL, Geng Y, Guo F, et al. Research progress on the mechanism of improving peanut yield by single-seed precision sowing. Journal of Integrative Agriculture. 2020; 19(8): 1919-1927.
6. Zhao XG, Chen LP, Gao YY, et al. Optimization method for accurate positioning seeding based on sowing decision. International Journal of Agricultural and Biological Engineering. 2021; 14(3): 171-180.
7. Kan Z, Gu CC, Wang LH, et al. Optimization of parameters for delinted cottonseeds dielectric selection. Transactions of the Chinese Society of Agricultural Engineering. 2010; 26(9): 114-119.
8. Chen BQ, Gao ZJ, Song TZ, et al. Scheme and algorithm of cottonseed selection based on image processing. Transactions of the Chinese Society for Agricultural Machinery. 2010; 41(1): 167-197.
9. Li, JB, Chen BQ, Shao LH, et al. Variety identification of delinted cottonseeds based on BP neural network. Transactions of the Chinese Society of Agricultural Engineering. 2012; 28(z2): 265-269.
10. Yu SH, Liu YL, Wu SP, et al. Design and Simulation Analysis of Dual CCD Detection System for Delinted Cottonseeds Color Sorter. Transactions of the Chinese Society for Agricultural Machinery. 2015; 46(9): 55-61.
11. Yu SH, Liu YL, Wu SP, et al. Implementation of Delinted Cottonseeds Color Sorter Based on FPGA. Transactions of the Chinese Society for Agricultural Machinery. 2015; 46(8): 20-26.
12. Xu BB, Cen KT, Huang JJ, et al. A Survey on Graph Convolutional Neural Network. Chinese Journal of Computers. 2020; 43(5): 755-780.
13. Zhang ZW, Cui P, Zhu WW. Deep Learning on Graphs: A Survey. IEEE Transactions on Knowledge and Data Engineering. 2022; 34(1): 249-270.
14. Tu KL, Wen SZ, Cheng Y, et al. A non-destructive and highly efficient model for detecting the genuineness of maize variety 'JINGKE 968' using machine vision combined with deep learning. Computers and Electronics in Agriculture. 2021; 182: 06002.
15. Javanmardi S, Ashtiani SHM, Verbeek FJ. Computer-vision classification of corn seed varieties using deep convolutional neural network. Journal of Stored Products Research. 2021; 92: 101800.
16. Yang XJ, Guo M, Lyu QS, et al. Detection and classification of damaged wheat kernels based on progressive neural architecture search. Biosystems Engineering. 2021; 208, 176-185
17. Qadri S, Furqan Qadri S, Razzaq A, et al. Classification of canola seed varieties based on multi-feature analysis using computer vision approach. International Journal of Food Properties. 2021; 24(1): 493-504.
18. Du J, Hu BL, Liu YZ, et al. Study on Quality Identification of Macadamia nut Based on Convolutional Neural Networks and Spectral Features. Spectroscopy and Spectral Analysis. 2018; 38(5): 1514-1519.
19. Przybyło J, Jabłoński M. Using Deep Convolutional Neural Network for oak acorn viability recognition based on color images of their sections. Computers and electronics in agriculture. 2019; 156: 490-499.
20. Altuntaş Y, Cömert Z, Kocamaz AF, et al. Identification of haploid and diploid maize seeds using convolutional neural networks and a transfer learning approach. Computers and electronics in agriculture. 2019; 163: 104874.
21. Ni C, Wang D, Vinson R, et al. Automatic inspection machine for maize kernels based on deep convolutional neural networks. Biosystems engineering. 2019; 178: 131-144.
22. Zhang SY, Zhang QJ, Li K, et al. Detection of peanut kernel quality based on machine vision and adaptive convolution neural network. Transactions of the Chinese Society of Agricultural Engineering. 2020; 36(4): 269-277.
23. Zhu SP, Zhuo JX, Huang H, et al. Wheat Grain Integrity Image Detection System Based on CNN. Transactions of the Chinese Society for Agricultural Machinery. 2020; 51(5): 36-42.
24. Yang WG, Huai YJ, Zhang FQ, et al. Grape Seed Classification Based on Gabor and Deep Neural Network. Journal of University of Electronic Science and Technology of China. 2020; 49(1): 131-138.
25. Gulzar Y, Hamid Y, Soomro AB, et al. A Convolution Neural Network-Based Seed Classification System. Symmetry. 2020; 12(12): 2018.
26. Loddo A, Loddo M, Di Ruberto C, et al. A novel deep learning based approach for seed image classification and retrieval. Computers and Electronics in Agriculture. 2021; 187: 106269.
27. Kurtulmuş F. Identification of sunflower seeds with deep convolutional neural networks. Journal of Food Measurement and Characterization. 2021; 15(2): 1024-1033.
28. Zhao GY,Quan LZ, Li HL, et al. Real-time recognition system of soybean seed full-surface defects based on deep learning. Computers and Electronics in Agriculture. 2021; 187: 106230.
29. Vidyarthi SK, Singh SK, Xiao HW, et al. Deep learnt grading of almond kernels. Journal of Food Process Engineering. 2021; 44(4): e13662.

30. Koklu M, Cinar I, Taspinar Y S, et al. Classification of rice varieties with deep learning methods. Computers and Electronics in Agriculture. 2021; 187: 106285.
31. Sreedhar P, Satya S, Nandhagopal N, et al. Classification Similarity Network Model for Image Fusion Using Resnet50 and GoogLeNet. Intelligent automation and soft computing. 2022; 31(3): 1331-1344.
32. Zhang WW, Ma HM, Li XH, et al. Imperfect Wheat Grain Recognition Combined with an Attention Mechanism and Residual Network. Applied Sciences. 2021; 11(11): 5139.
33. Khan RU, Wong WS, Ullah I, et al. Evaluating the Efficiency of CBAM-Resnet Using Malaysian Sign Language. Cmc-computers materials & continua. 2022; 71(2): 2755-2772.
34. Wan P, Zhao JW, Zhu M, et al. Freshwater fish species identification method based on improved ResNet50 model. Transactions of the Chinese Society of Agricultural Engineering. 2021; 37(12): 159-168.
35. Huang S, Fan X, Sun L, et al. Research on Classification Method of Maize Seed Defect Based on Machine Vi-sion. Journal of Sensors. 2019; 2019:1-9.
36. Zhu S, Zhang J, Chao M, et al. A Rapid and Highly Efficient Method for the Identification of Soybean Seed Varieties: Hyperspectral Images Combined with Transfer Learning. Molecules. 2019; 25(1):152.
37. Yang Y, Chen JP, He Y, et al. Assessment of the vigor of rice seeds by near-infrared hyperspectral imaging combined with transfer learning. 2020; 10(72): 44149-44158.
38. Zeng G. On the confusion matrix in credit scoring and its analytical properties. Communications in Statistics-Theory and Methods. 2020: 49(9): 2080-2093.
39. Düntsch I, Gediga G. Indices for rough set approximation and the application to confusion matrices[J]. International Journal of Approximate Reasoning. 2020; 118: 155-172.
40. Luque A, Mazzoleni M, Carrasco A, et al. Visualizing Classification Results: Confusion Star and Confusion Gear. IEEE Access. 2021; 10: 1659-1677.

Fig 1. Cotton seed acquisition(a)

Click here to access/download;Figure;Fig 1. Cotton seed acquisition(a).tif

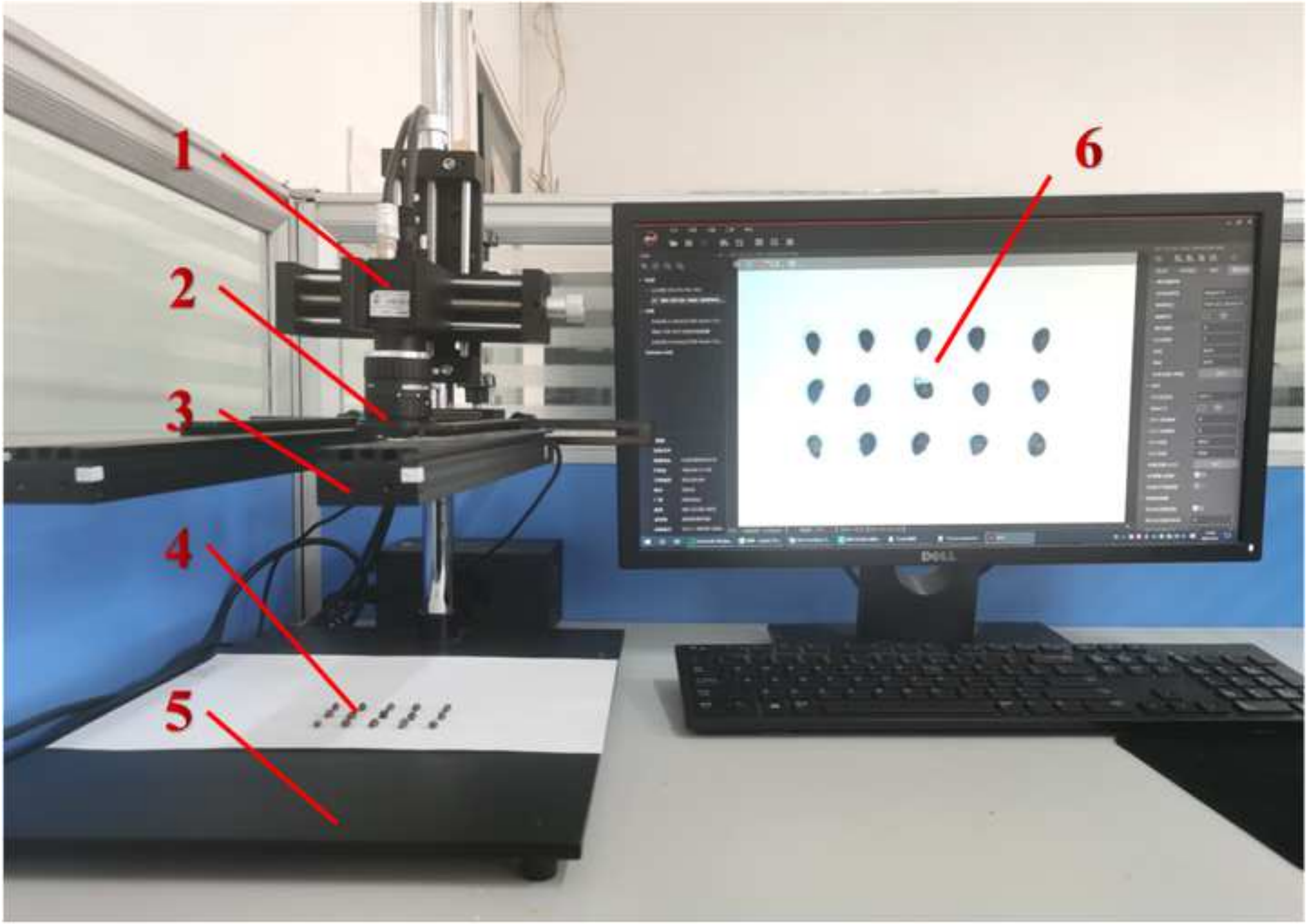

 Click here to access/download;Figure;Fig 1. Cotton seed acquisition (b).tif

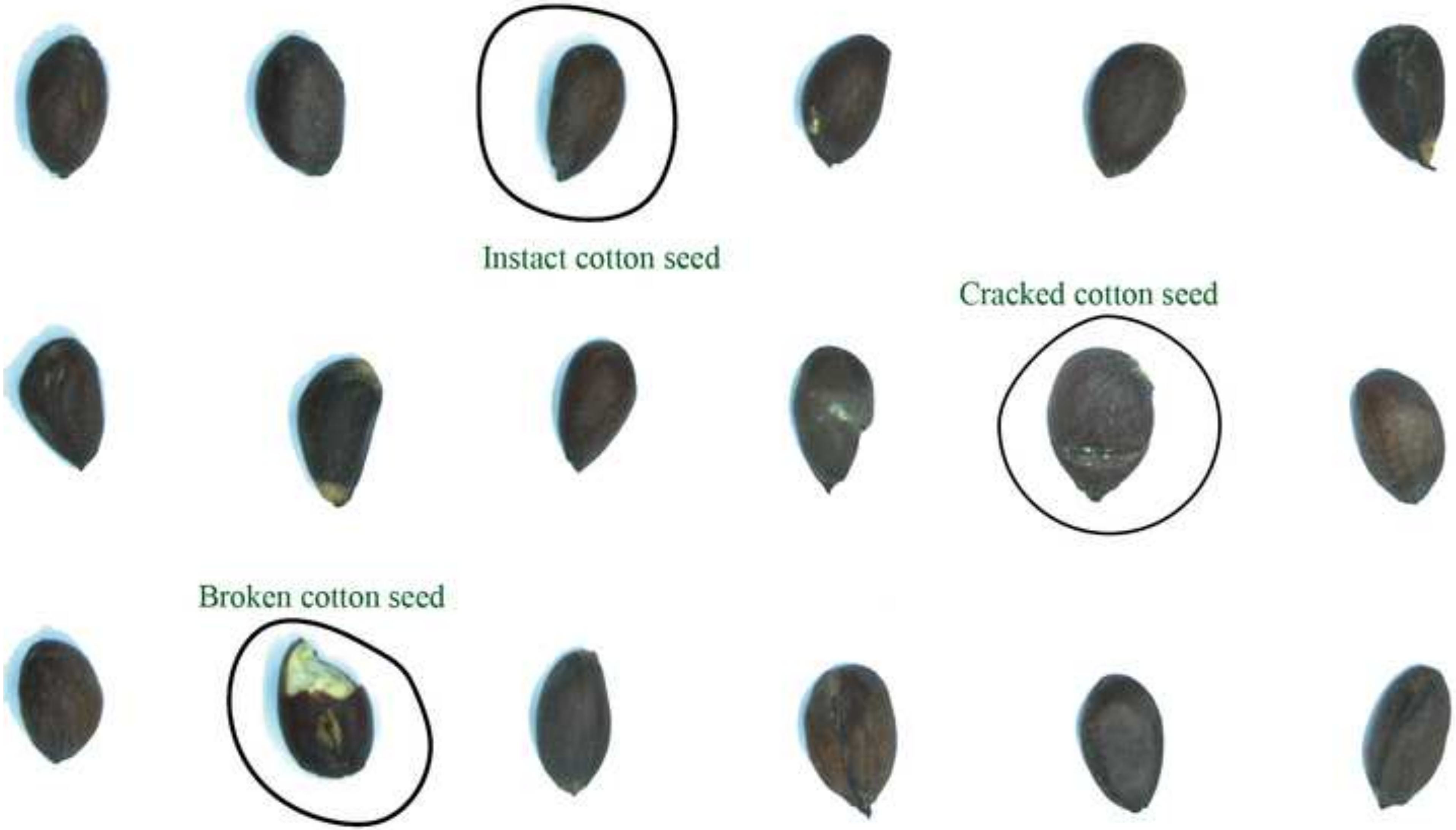

Fig 2. Single cotton image.

Click here to access/download;Figure;Fig 2. Single cotton image..tif

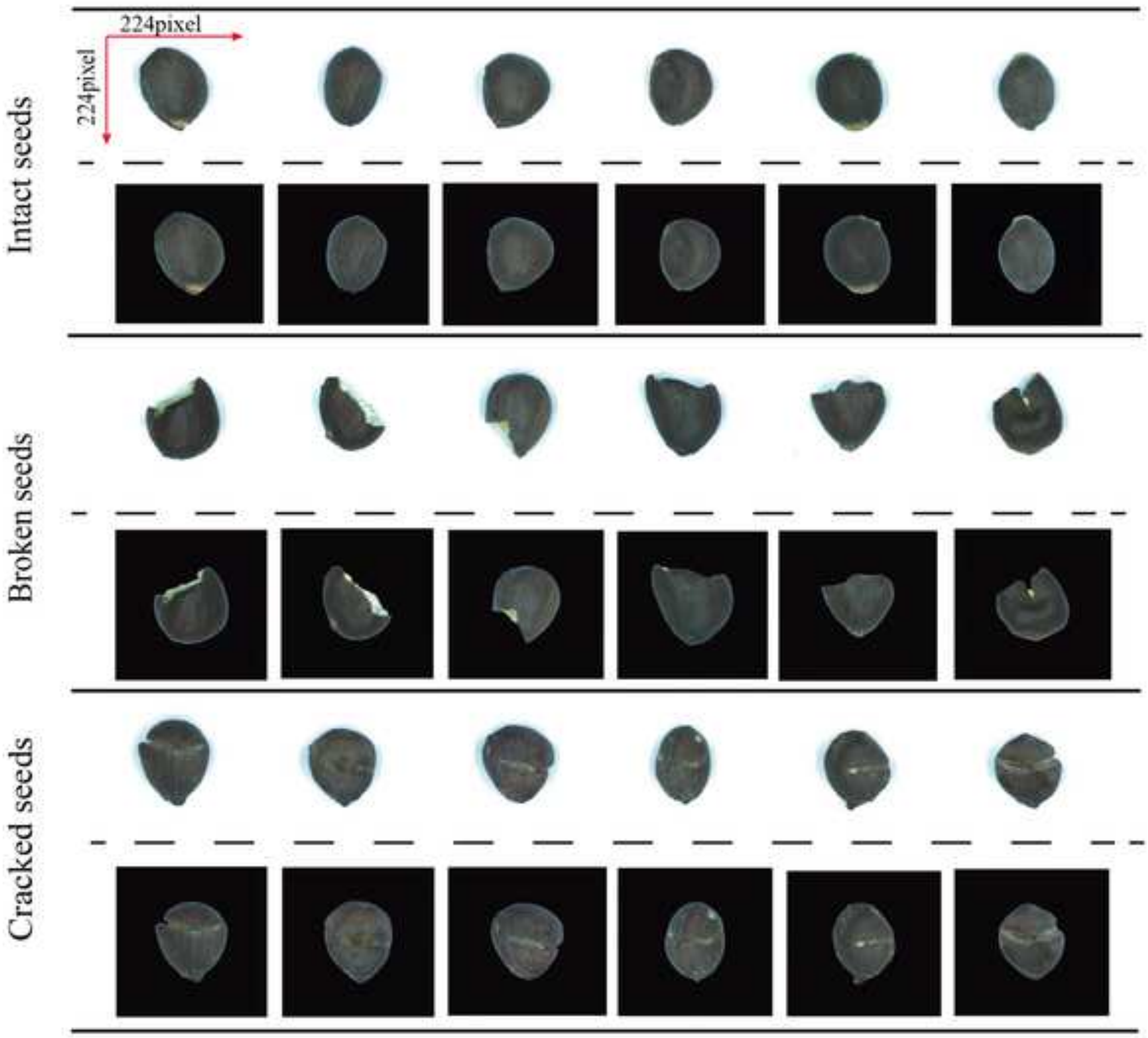

Fig 3. The network structure of Resnet50 model

Click here to access/download;Figure;Fig 3. Network structure of Resnet50 model..tif

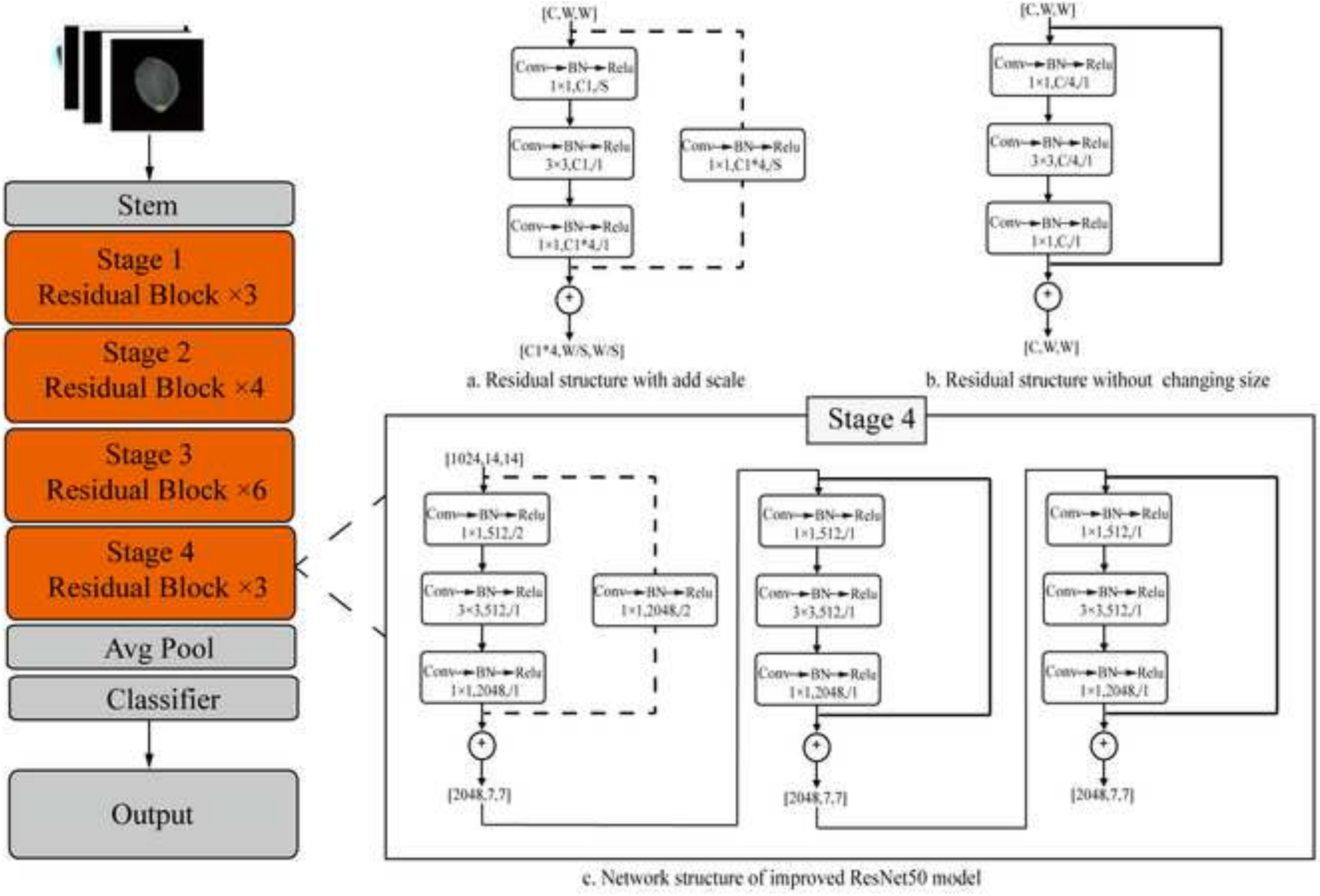

Fig 4. CBAM module

Click here to access/download;Figure;Fig 4. CBAM module..tif

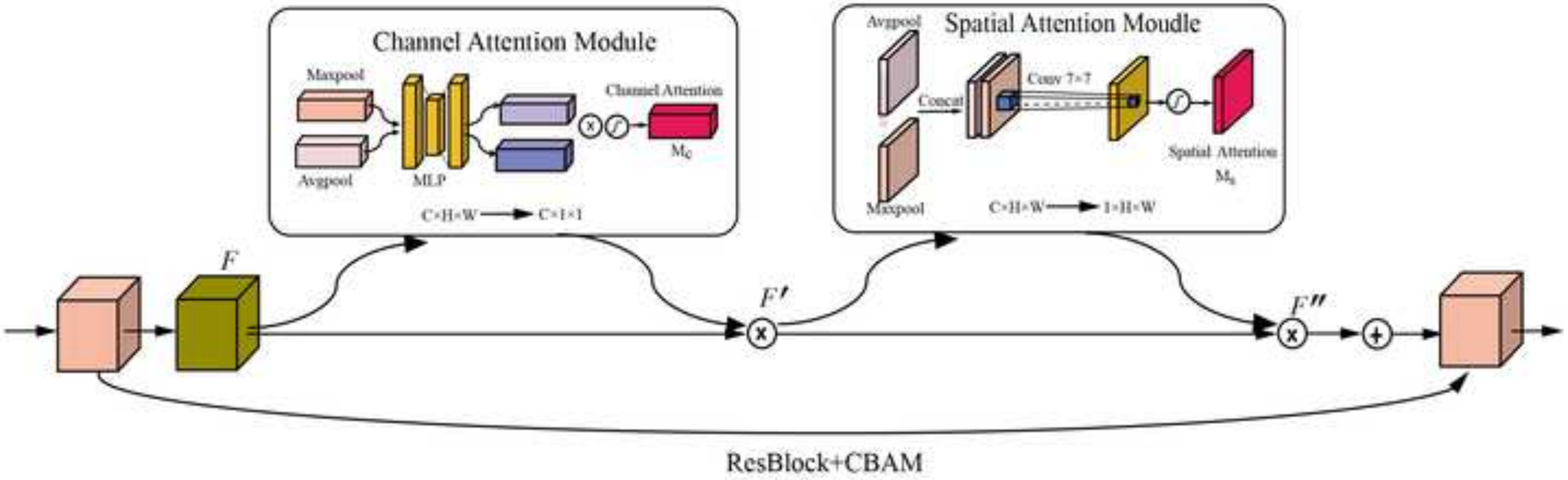

Click here to access/download;Figure;Fig 5. Improved ResNet50 model..tif

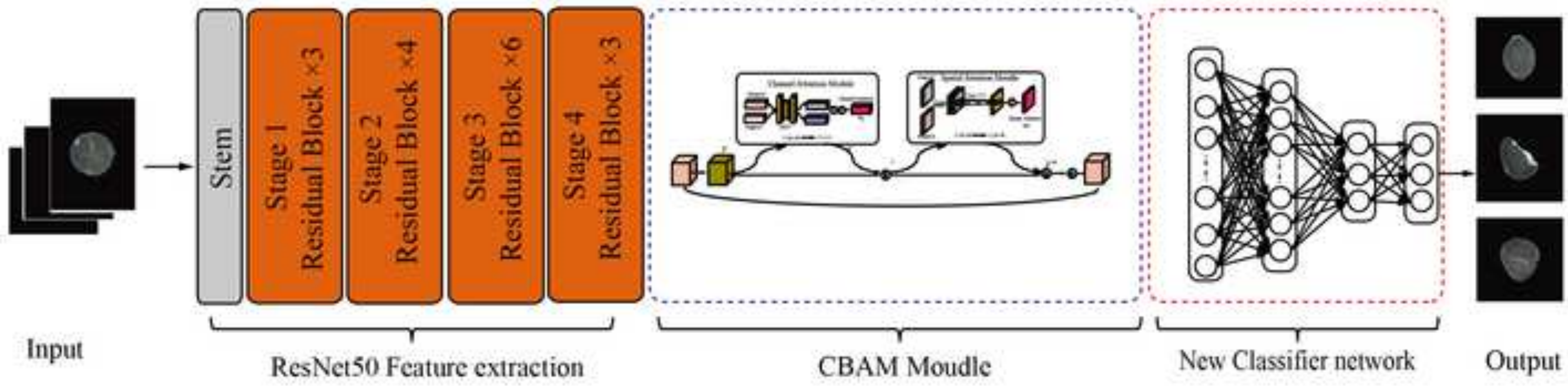

 Click here to access/download;Figure;Fig 6. LRelu-Softplus activation function..tif

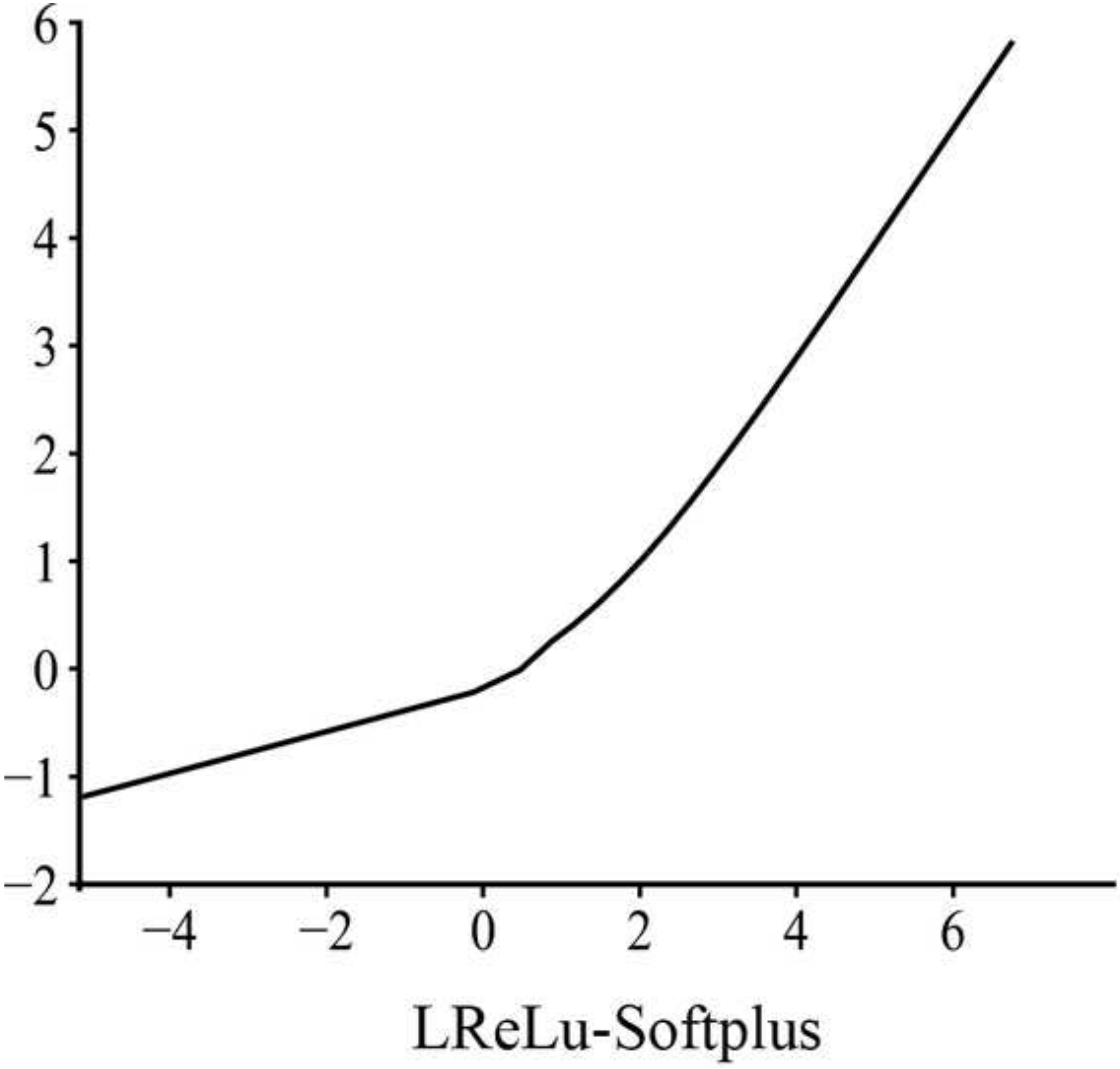

Fig 7. The Full connection layer and Dropout

Click here to access/download;Figure;Fig 7. The Full connection layer and Dropout.tif

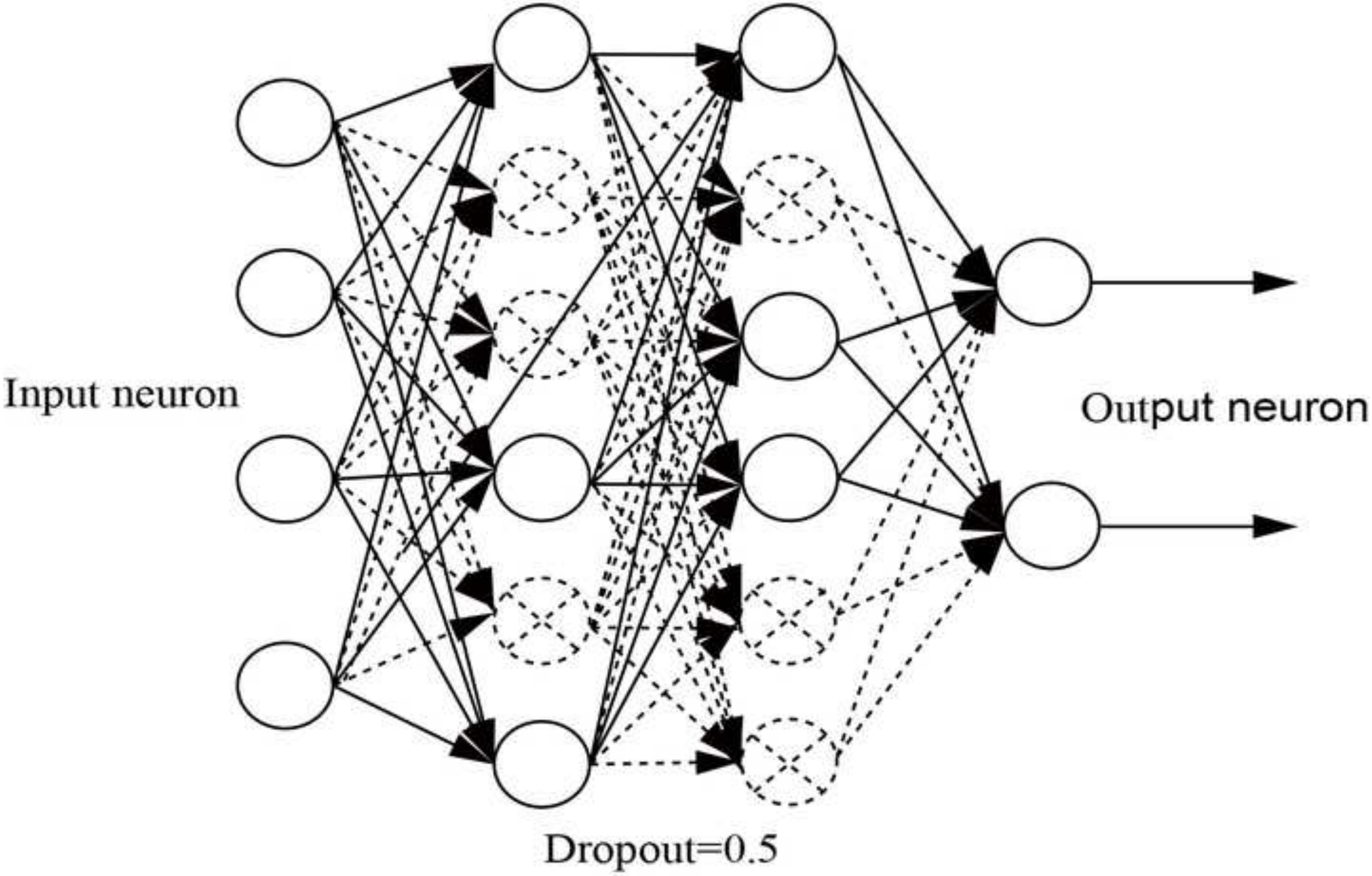

Fig 8. Procedure for training

Click here to access/download;Figure;Fig 8. Procedure for training..tif

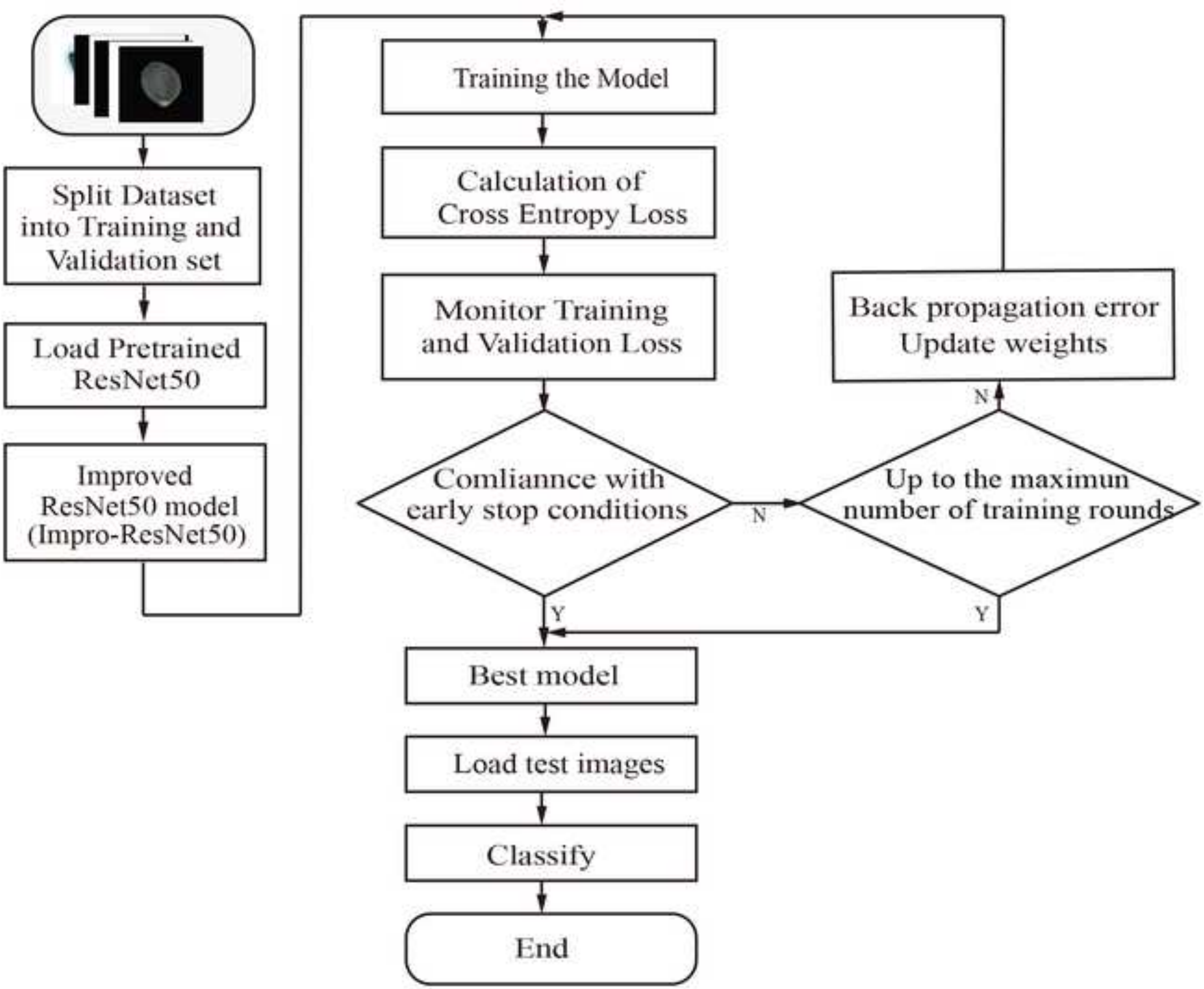

Fig 9. Effects of learning rate on training accuracy and training loss

Click here to access/download;Figure;Fig 9. learning rate.tif

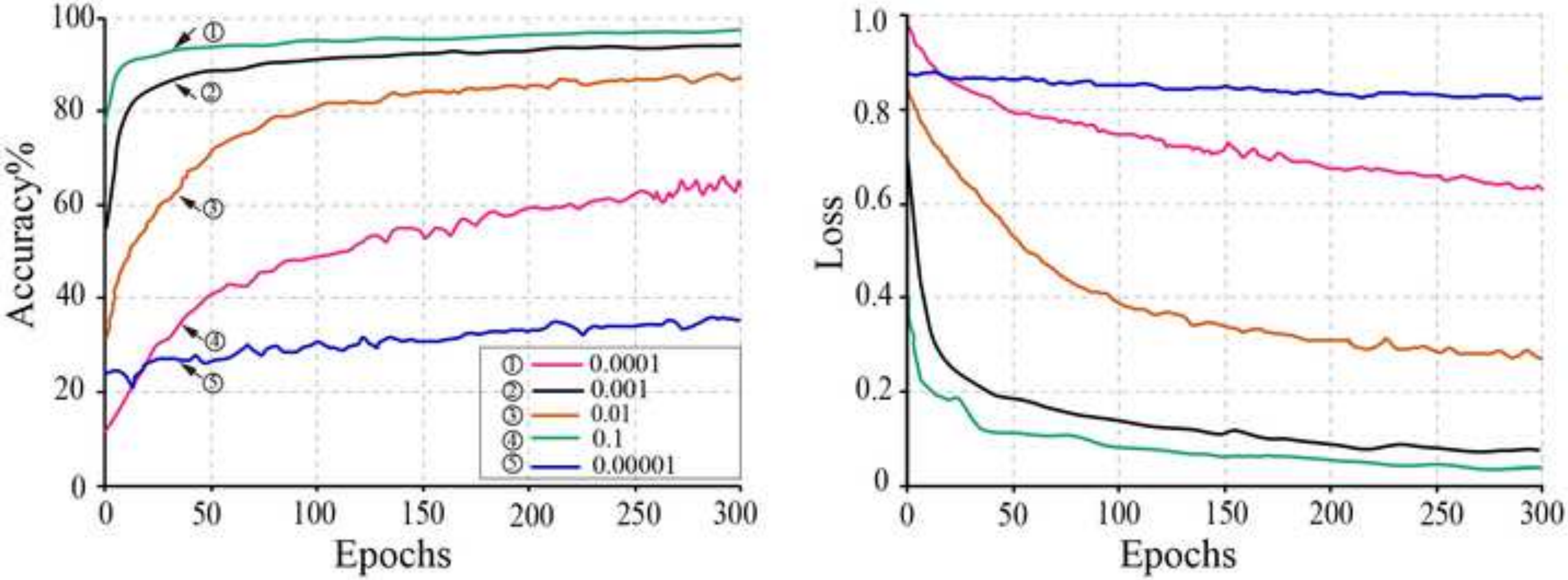

Fig 10. Effects of activation function on training accuracy and training loss

Click here to access/download;Figure;Fig 10. activation function.tif

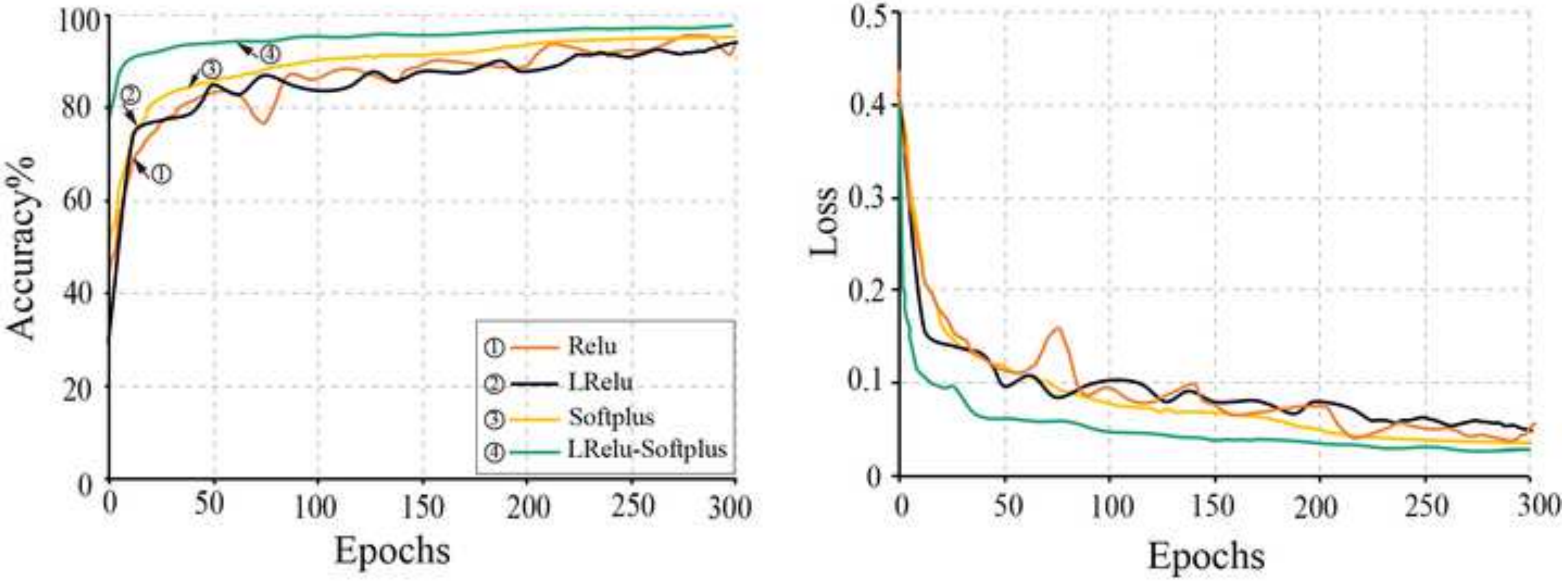

Fig 11. Effects of fully connected layers on training accuracy and training loss

Click here to access/download;Figure;Fig 11. fully connected layers.tif

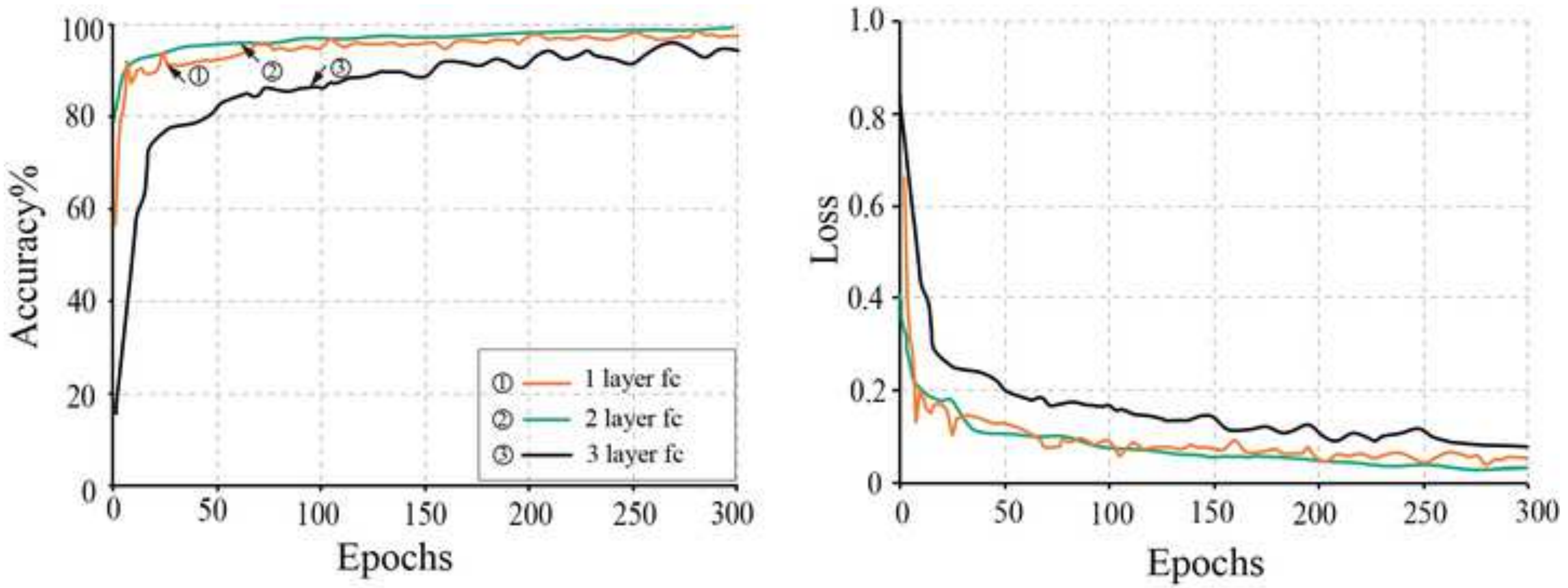

Fig 12. Performance comparison between improved-ResNet50 model and different classical models

Click here to access/download;Figure;Fig 12. Performance comparison.tif

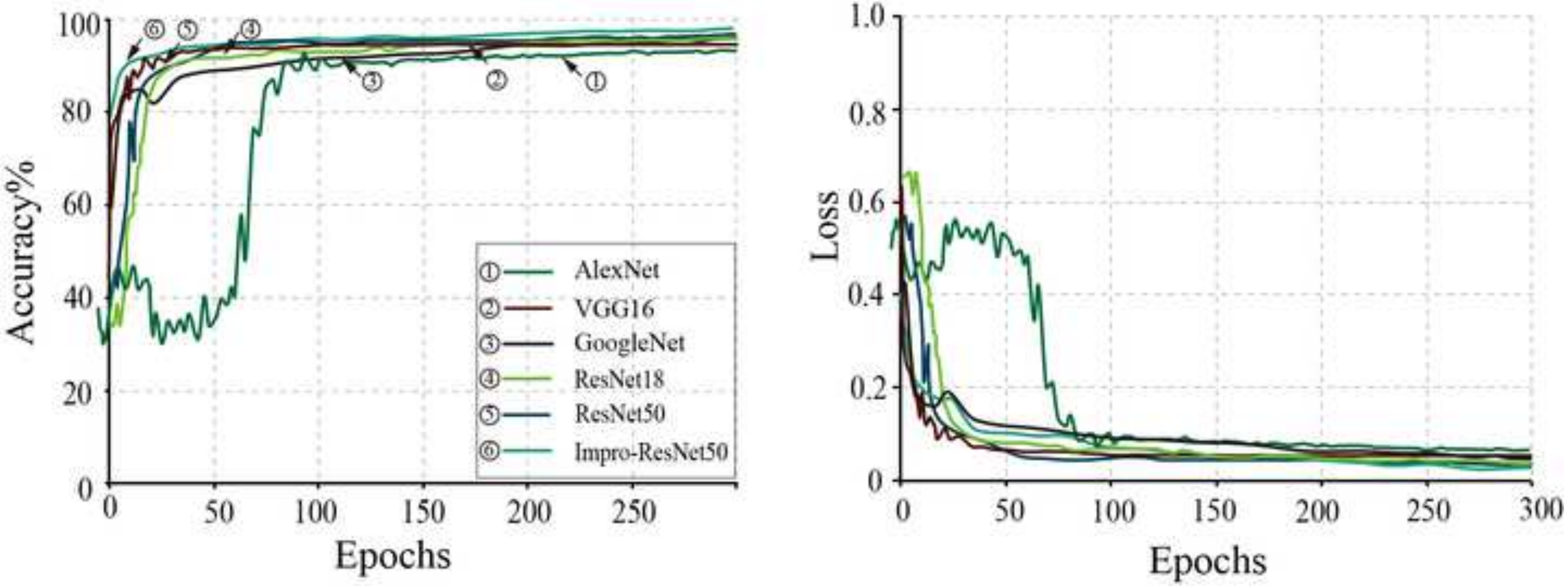

Fig 13. Comparison of Type Identification Results of Several Models

Click here to access/download;Figure;Fig 13. Identification Results.tif

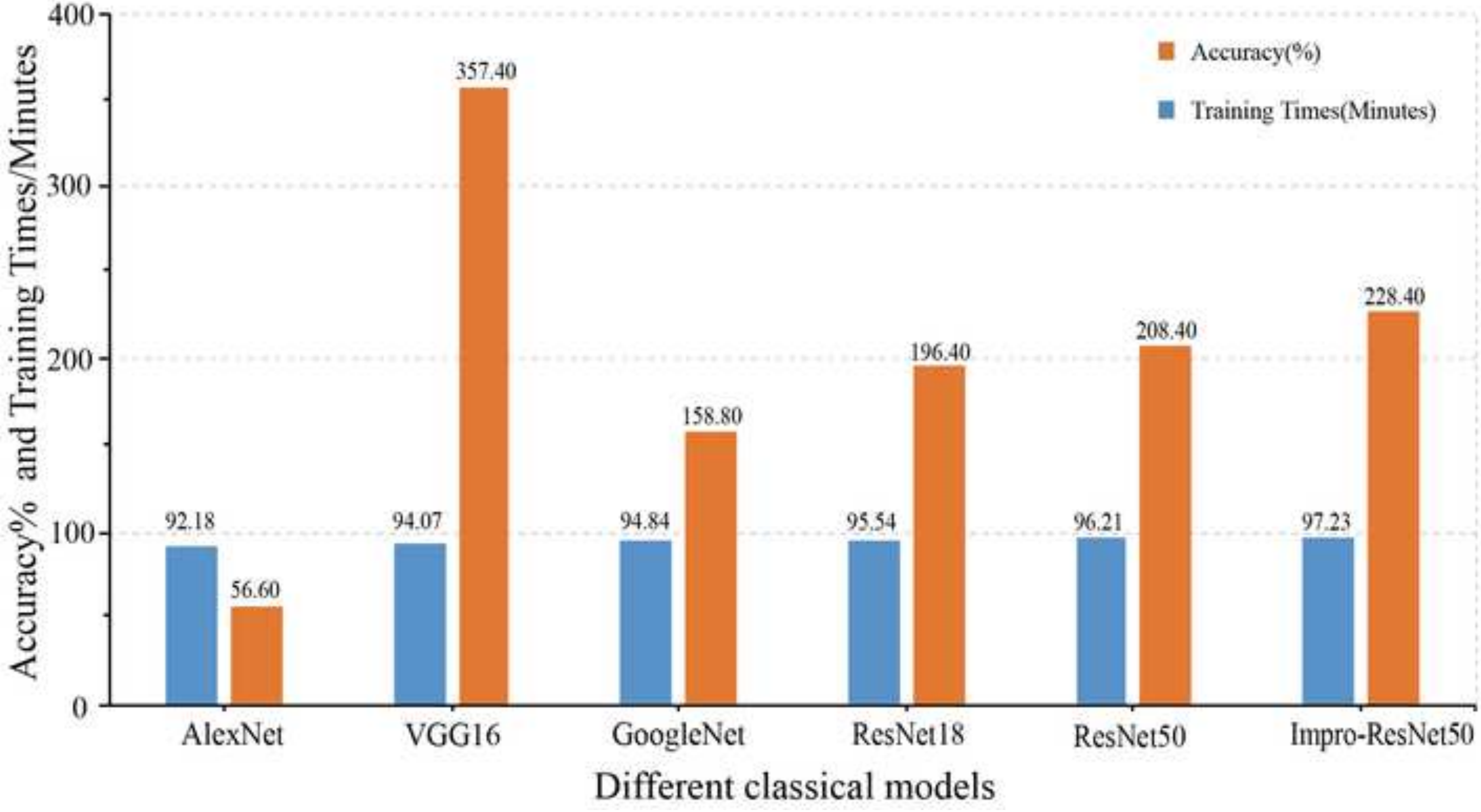

Supplement: Attachment — Submitted filename: PONE-D-22-02418_reviewer.pdf [file pone.0273057.s002.pdf]
