# Supplementary material for: A method for detecting the quality of cotton seeds based on an improved ResNet50 model
Source: PLoS One. 2023 Feb 15;18(2):e0273057. doi: 10.1371/journal.pone.0273057 (PMC9931132; doi:10.1371/journal.pone.0273057)
Supplement: Attachment — Submitted filename: Response to Reviewers.docx [file pone.0273057.s003.docx]

Dear Editor and reviewers:

Thank you so much for your letter dated April 15. We were pleased to know that our work was rated as potentially acceptable for publication in Journal, subject to adequate revision. We acknowledge the reviewers for the time and effort that they have put into reviewing the previous version of the manuscript. Their suggestions have helped us to improve our work. Based on the instructions provided in your letter, we uploaded the file of the revised manuscript. At the same time, we have uploaded a marked-up copy of the original manuscript file, highlighting the changes made to the original version.

Appended to this letter is our point-by-point response to the comments raised by the reviewers. The comments are reproduced and our responses are given directly afterwards in a different colour (red).

We would be grateful to you for allowing us to resubmit a revised copy of the manuscript.

We hope that the revised manuscript selects for publication in the PloS ONE.

Sincerely

#Reviewer 1：

Comments to the Author

**1- According to the authors: "The results show that the enhanced ResNet50 model can achieve an average recognition accuracy of 97.23%,approximately 5.05%, 3.16%, 2.39%,1.69%, and 1.02% better than several classical models of AlexNet, VGG16, GoogleNet, ResNet18, and ResNet50, respectively". What is the reference model or the control, and how did the it perform? The authors did not perform a clear comparative analysis among the models. How is it possible to make such conclusion?**

Response: We have no clear comparative analysis problem for several models according to you. Regarding this point, when I re-read the manuscript, I feel that there are indeed some problems as a teacher said, the original manuscript is only expressed by a single bar graph and there are some errors in the graphs, which do not clearly represent the desired results to be shown. Therefore, I reorganized the experimental data in the revised manuscript and added the model training curve in Fig 12, the average recognition accuracy of the model in Fig 13 and the statistical values of the model training results in Table 3 for the performance comparison of several models.

**Fig 12. Performance comparison between improved-ResNet50 model and different classical models.**

**Fig 13. Comparison of the type recognition results of several models.**

**Table 3. Comparison of Impro-ResNet50 performance with different classical models.**

| **Model names** | **Epoch** | **Error-rate %** | **Validation Loss** | **Training Time /mins: secs** | **Accuracy/%** |
| --- | --- | --- | --- | --- | --- |
| AlexNet | 300 | 8.621 | 0.3727 | 56.60 | 92.189 |
| VGG16 | 300 | 5.9280 | 0.1818 | 357.40 | 94.071 |
| GoogLeNet | 300 | 5.1580 | 0.1484 | 158.80 | 94.842 |
| ResNet18 | 300 | 4.4534 | 0.1402 | 196.40 | 95.546 |
| ResNet50 | 300 | 3.7853 | 0.1014 | 208.40 | 96.214 |
| Impro-ResNet50 | 300 | 2.7687 | 0.0827 | 228.40 | 97.231 |

**2- Clearly state explain CBAM, LED, CDD and other abbreviations/acronyms the first time there appear in the in the manuscript.**

Response: Due to personal negligence in writing the paper, the full names of the acronyms CBAM, LED, CDD, etc., were not clearly explained. CBAM: Convolutional block attention module, LED: Light-emitting diode, CCD: Charge-coupled device.

**3- Reformat tables according to PLOS ONE requirements.**

Response: By carefully reading the table arrangement requirements of ' PloS ONE ' and consulting recent periodical publications, all tables' titles and text format errors are corrected.

Thank you for taking the valuable time to review my article. Following teacher instructions, the article structure is more scientific and rigorous.

#Reviewer 2：

Comments to the Author

**1-The paper was insightful introducing the Impro-ResNet50 model for cotton seed identification of three types of cotton seeds: intact cotton seeds, broken cotton seeds, and cracked cotton seeds. But there were minor grammatical and typographical errors especially iin the introduction that needs attention. Also, the experimental process should be separated from the results and discussion.**

Response: According to your review comments, I corrected the grammatical errors and typographical errors in the introduction part and the reference citation marking problems, which I had not found carelessly. I thank you for reviewing the article carefully and pointing out the problems one by one. In addition, regarding the teacher's suggestion to separate the experimental procedure from the results and discussion, my previous paper structure was indeed not rigorous enough, and I redesigned the paper structure in the rewrite. The main structure of the thesis is shown in the figure below.

**Fig.14 Main flow chart of the article.**

Thank you very much for your guidance, the structure of the paper is more scientific and compact by adjusting the form of the article.

Thank you so much to the editors and reviewers for your comments on my paper, which made me benefit a lot. I am now resubmitting the revised draft, and hope that the revised article will meet the requirements of the teachers, of course, if there are still problems in the paper, I hope you can be kind enough to point them out, and I will continue to improve until I meet your requirements. I hope the journal will accept my article and I look forward to listening to you.

Finally, I would like to thank the editors and reviewers again for their help.
