# Supplementary material for: A method for detecting the quality of cotton seeds based on an improved ResNet50 model
Source: PLoS One. 2023 Feb 15;18(2):e0273057. doi: 10.1371/journal.pone.0273057 (PMC9931132; doi:10.1371/journal.pone.0273057)
Supplement: Attachment — Submitted filename: Response to Reviewers.docx [file pone.0273057.s004.docx]

Dear Editor and reviewers:

Thank you so much for your letter of June 10. We are pleased that our paper "A Method for Detecting the Quality of Cotton Seeds Based on an Improved ResNet50 Model (ID: PONE-D-22-02418R1)" may be published in your journal. However, sufficient revisions are needed. We are very happy to have this opportunity. We acknowledge the reviewers for the time and effort they put into reviewing the previous version of the manuscript. With your valuable comments, I can improve my paper, which will also help me in my future work. Once again, my heartfelt thanks.

We hope that the revised manuscript will be selected for publication in PloS ONE. I am looking forward to hearing from you.

Sincerely

#Reviewer 1：

Comments to the Author

1- Abstract
“This paper proposes … convolutional block attention module(CBAM)”. Please put a space between the module and CBAM. Please correct this type of error throughout the manuscript.

**Answer:** The similar error in the paper has been corrected. Thank you for your precious comments.

2-Introduction

(1) The authors said: “In the production of cotton seeds, by introducing the principles of air screen, magnetic screen, socket screen, gravity screen, dielectric screen, and other tenets of mechanized sorting equipment to replace the manual screening, significantly improving the screening efficiency of cotton seeds in actual production, this method can screen grain whole cotton seeds in large quantities.” Which method are the authors referring to? Please reformulate the whole sentence to make it clearer.

**Answer:** Thank you for your valuable comments. This revision has modified the introduction section significantly. Considering the logic of the context, this statement has been deleted in this revision.

(2) Reformulate the following sentence: “Machine vision technology for the non-destructive testing application of research a lot, broad prospects, traditional machine vision technology through the linear discriminant model, artificial neural network, support vector machine and other algorithms can remove the immature cotton seed, broken cotton seed.”

**Answer:** As you commented, this expression has a big problem. Therefore, a great deal of adjustments has been made in making changes. The partial modifications are as follows.

The revised sentence is “Machine learning-based image processing techniques have been successfully applied to detect seed quality with the advancement of computer vision technology [6-8]. The researchers conduct seed quality assessment by extracting features such as texture, color and shape of the seed images. This method is more advanced and effective in detecting seed quality than the manual method. However, the method is relatively dependent on manual feature extraction, and different features require different extraction methods. In addition, manual feature extraction is usually inadequate. Thus, it leads to the detection accuracy of the method is not high.”

Thank you for your valuable comments, and I hope the revisions will meet with your approval.

(3) The sentence: “In recent years, the deep learning technology represented by convolutional neural networks has been widely used in the field of image recognition; compared with the traditional image detection technology, convolutional neural networks have increased the deep network structure with feature learning, which can automatically extract the most representative image features” should be splitted into two sentences for more clarification. "Compared with ... image features." might become a separate sentence.

**Answer:** As in question (2), the sentence has been changed considerably. The partial modifications are as follows.

The revised sentence is, “There has been an increase in convolutional neural networks used for image recognition [9-11]. In addition to simulating the human brain's mechanism for extracting features in layers, the technique can extract features automatically from simple to complex, from bottom to top, and from concrete to abstract. Several researchers have successfully applied CNN to the detection of seed quality [12-15]. But, a disadvantage of CNN detection is that it requires a large amount of training data, is time-consuming, and computationally resource-intensive.”

Thank you again for your valuable comments.

(4) In the sentence “Researchers at home and abroad apply convolutional neural networks to seed quality detection of corn, wheat grains, peanuts and other crops,” what do the authors mean by researchers at home and abroad? Reformulate it in a scientifically sound way.

**Answer:** As you commented, the phrase is incorrectly phrased. I have made the following corrections.

The revised sentence is, “There has been an increase in convolutional neural networks used for image recognition [9-11]….. Several researchers have successfully applied CNN to the detection of seed quality [12-15]. But, a disadvantage of CNN detection is that it requires a large amount of training data, is time-consuming, and is computationally resource-intensive.”

(5) The authors systematically add a point (.) after most citations regardless of their position in the sentences. Some examples are “Przybyło et al [19].”, “Altuntaş et al [20].” and “Ni et al [21].” to list the least. This makes it difficult for a reader to understand the sentences.

**Answer:** According to your valuable comments, we have carefully revised these sentences in the paper.

The revised sentence is, “ For instance, the authors of [24] demonstrated a CNN-based transfer learning method for detecting haploid and diploid maize seeds. The model achieved optimal detection accuracy of 94.22%, providing technical support for the non-destructive, rapid, and inexpensive detection of high-quality seeds. In [25], the authors developed a peanut seed quality detection method based on machine vision and an adaptive CNN. The process achieved an average detection accuracy of 99.70% for common peanut seeds, such as mouldy, broken, or shrivelled….”

Due to a large number of changes, please read the paper for details. Thank you again for your careful instruction.

3-Methodology.

(1) Please clearly organise this section in an appropriate manner to avoid confusion.

**Answer:** According to your valuable comments, we have reorganized that part of the paper. Due to a large number of changes, please read the paper for details. Thank you again for your careful instruction.

(2) From the subsection “Model training and parameter tuning” to “Comparison of different neural network models,” there was a huge confusion between methods and results. Everything relating to a result should be moved to the results section.

**Answer:** As you mentioned, the section is somewhat confusing. Therefore, we have made a more significant change in the paper. Due to a large number of changes, please read the paper for details. Thank you again for your careful instruction.

4-Results.

(1). “Results and Discussion.” Please delete discussion from the section title.

**Answer:** Thank you for your valuable comments. The section has been carefully revised. Due to a large number of changes, please read the paper for details. Thank you again for your careful instruction.

(2). Please integrate comments provided in the methodology and reorganize the results section accordingly.

**Answer:** I have substantially revised that section to meet your expectations. This revision made me understand the research better. In addition, it has a vital role in improving the quality of the paper. Due to a large number of changes, please read the paper for details. Thank you again for your careful instruction.

5-Discussion.

This section needs significant improvement. The authors should interpret their key results and relate them to previous investigations/studies in the field.

**Answer:** Thanks to your valuable comments, significant revisions have been made to the experimental analysis, discussion of results, etc., of the paper. This revision has helped and will continue to influence my future writing. Once again, I express my sincere gratitude. Due to a large number of changes, please read the paper for details. Thank you again for your careful instruction.

#Reviewer 3：

Comments to the Author

1- Add Literature review or related works section.

**Answer:** As you suggested, add a literature review or a section on related works to the paper. In addition, this revision removes older literature and adds more recent literature to increase persuasiveness.

2- Numberings for the equations are wrong.

**Answer:** Thank you for your careful review, and the numbering of the equations in the paper has been corrected.

3-Whether hyperparameter tuning is done?

**Answer:** Detailed parameter settings are performed in section 5.1 of the paper. Comparison tests with different learning rates, activation functions and fully connected layers are performed to correct the parameters in Section 6.1. Due to a large number of changes, please read the paper for details. Thank you again for your careful instruction.

4-Add future works at the end of conclusion section.

**Answer:** As you suggested, future work has been added at the end. The main points are the following.

The Impro-ResNet50 cotton seed quality detection model, based on the attention mechanism, was trained on a large amount of data while maintaining high accuracy and requiring only a short amount of time to run. In the future, we will supplement the data with cotton seeds of different qualities and backgrounds so that the model has a wider range of applications. At the same time, the model is simplified so that it can be deployed on mobile and easily used by farmers.

5-The contribution of the works should be added as points in the introduction section.

**Answer:** As you suggested, the main contributions and innovations of the paper have been added in the introduction section as relevant. The main points are the following.

(1).	Based on the appearance of defects in cotton seed, a new cotton seed dataset is created to support the development of subsequent detection algorithms.

(2).	The Impro-ResNet50 model is proposed as a new method for detecting cotton seed quality based on an attention mechanism. The CBAM attention block is embedded in ResNet50 to integrate feature channel and spatial information attention and enhance the model's capacity to learn essential information about cotton seed regions.

(3).	The model's application serves as a reference for developing new models, demonstrating the interoperability of deep learning models and attention mechanisms.

(4).	On the basis of the cotton seed quality identification dataset, Impro-ResNet50 is subjected to extensive comparative experiments. Impro-ResNet50 is highly accurate and robust in cotton seed detection tasks, demonstrating the efficacy of the CBAM module. Provide technical support for developing cotton seed quality testing equipment in the future.

6-English language check needed

**Answer:** I appreciate your insightful feedback. According to your suggestion, I concentrate on revising long sentences, tenses, and repetitive descriptions in my paper. Subsequently, I have invited some colleagues proficient in English writing to review it. I sincerely hope that the revised paper will satisfy your needs.

Thank you so much to the editors and reviewers for your comments on my paper, which benefited me greatly. I am now resubmitting the revised draft and hope that the revised article will meet the requirements of the teachers. Of course, if there are still problems in the paper, I hope you can be kind enough to point them out, and I will continue to improve until I meet your requirements. I hope the journal will accept my article, and I look forward to listening to you.

Finally, I would like to thank the editors and reviewers again for their help.
